# Supplementary material for: Effect of lifting COVID-19 restrictions on utilisation of primary care services in Nepal: a difference-in-differences analysis
Source: BMJ Open. 2022 Nov 29;12(11):e061849. doi: 10.1136/bmjopen-2022-061849 (PMC9709811; doi:10.1136/bmjopen-2022-061849)
Supplement: Supplementary data [file bmjopen-2022-061849supp001.pdf]

Title: Effect of lifting COVID-19 restrictions on utilisation of primary care services in Nepal: a difference-in-differences analysis

Authors: Kapoor NR, Aryal A, Mehata S, Dulal M, Kruk ME, Bauhoff S, Arsenault C

Supplemental materials

Supplemental Table 1. Additional sources used for COVID-19 restrictions tracking

**Additional Sources:**
48 dists currently under DAO prohibitory order. The Rising Nepal. 2020; published online Aug 31. https://old.risingnepaldaily.com/main-news/48-dists-currently-under-dao-prohibitory-order.
Curfew Relaxed In Kaski, Public Buses Not Plying, Taxis And Private Vehicles Rampant. Arthik Awaj. 2020; published online Aug 28. https://arthikawaj.com/%e0%a4%aa%e0%a5%8b%e0%a4%96%e0%a4%b0%e0%a4%be%e0%a4%ae%e0%a4%be-%e0%a4%a8%e0%a4%bf%e0%a4%b6%e0%a5%87%e0%a4%a7%e0%a4%be%e0%a4%9c%e0%a5%8d%e0%a4%9e%e0%a4%be-%e0%a4%96%e0%a5%81%e0%a4%95%e0%a5%81%e0%a4%b2/.
Four local levels in Jhapa impose weeklong lockdown. Nepal Monitor. 2020; published online Aug 15. https://nepalmonitor.org/reports/view/30662.
Indefinite prohibitory order imposed in Bara. Khabarhub. 2020; published online Aug 17. https://english.khabarhub.com/2020/17/120466/.
INFOGRAPHICS: 48 districts have partial or complete lockdown. myRepublica. 2020; published online Aug 24. http://myrepublica.nagariknetwork.com/news/98460/.
INFOGRAPHICS: These 52 districts are under lockdown. myRepublica. 2020; published online Aug 25. https://myrepublica.nagariknetwork.com/news/infographics-these-52-districts-are-under-lockdown/.
Kathmandu Valley back to lockdown. Nepali Times. 2020; published online Aug 18. https://www.nepalitimes.com/banner/kathmandu-valley-back-to-lockdown/.
Local levels in Kavrepalanchok being sealed off amidst COVID-19 fear. Khabarhub. 2020; published online Aug 15. https://english.khabarhub.com/2020/15/120082/.
Lockdown enforced in Byas Municipality for a week. Nepal Monitor. 2020; published online Aug 17. https://nepalmonitor.org/reports/view/30696.
Lockdown enforced in Dasarathchand Municipality of Baitadi. Nepal Monitor. 2020; published online Aug 20. https://nepalmonitor.org/reports/view/30790.
Lockdown enforced in five local levels of Sindhuli. Nepal Monitor. 2020; published online Aug 15. https://nepalmonitor.org/reports/view/30667.

Koirala N. Lockdown extended for another week in Bhojpur. The Himalayan Times. 2020; published online Sept 10. https://thehimalayantimes.com/nepal/lockdown-extended-for-another-week-in-bhojpur.
Nepal: Lockdown extended in Kathmandu Valley until September 16. Crisis 24. 2020; published online Sept 10. https://crisis24.garda.com/alerts/2020/09/nepal-lockdown-extended-in-kathmandu-valley-until-september-16-update-24.
Rai MC. Prohibition in 3 cities including Udaipur headquarters. Nagarik News. 2020; published online Aug 12. https://nagariknews.nagariknetwork.com/social-affairs/304061-1597214771.html.
Prohibition in Dang from 14th. Online Khabar. 2020; published online Aug 12. https://www.onlinekhabar.com/2020/08/892463.
Prohibitory order in Dhankuta extended by one week. Khabarhub. 2020; published online Aug 27. https://english.khabarhub.com/2020/27/123095/.
Prohibitory orders in 39 districts to curb COVID-19. The Rising Nepal. 2020; published online Aug 17. https://old.risingnepaldaily.com/mustread/prohibitory-orders-in-39-districts-to-curb-covid-19.
Ten days long lockdown imposed in Sankhuwasabha. Nepal Monitor. 2020; published online Aug 18. https://nepalmonitor.org/reports/view/30747.
Gautam L. Week-long lockdown imposed to prevent spread of coronavirus in Phidim. The Himalayan Times. 2020; published online Aug 28. https://thehimalayantimes.com/nepal/week-long-lockdown-imposed-to-prevent-spread-of-coronavirus-in-phidim.

Supplemental Table 2. Nepal DHIS2 Definitions for health service outcomes

**Health service**	**Nepal DHIS2 Definition**
**Outpatient visits**	Disaggregation by Sex & Caste/Ethnicity - Outpatient Cases
**Family planning**	Family Planning Program - Temporary FP Method - Depo-Current User +<br>Family Planning Program - Temporary FP Method - Depo-New Users < 20 Years +<br>Family Planning Program - Temporary FP Method - Depo-New Users > 20 Years +<br>Family Planning Program - Temporary FP Method - Pills- Current User +<br>Family Planning Program - Temporary FP Method - Pills- < 20 Years +<br>Family Planning Program - Temporary FP Method - Pills- > 20 Years +<br>Safe Motherhood Program-Safe Abortion Service-Post Abortion FP Methods Short Term-Medical +<br>Safe Motherhood Program-Safe Abortion Service-Post Abortion FP Methods Short term-Surgical +
**Antenatal care**	Safe Motherhood Program-Antenatal Checkup-First ANC visits (any time) < 20 years +<br>Safe Motherhood Program-Antenatal Checkup-First ANC visits (any time) > 20 years
**Postnatal care**	Safe Motherhood Program- Type of Delivery - 3 PNC visits as per protocol
**Pneumonia**	CBIMCI-(2-59Months)- Classification-ARI-Pneumonia +<br>CBIMCI-(2-59Months)-ORC Classification-ARI-Severe Pneumonia/Very Severe Disease
**Measles**	Immunization program - Children Immunized - Measles/Rubella - 9-11 Months + Immunization program - Children Immunized - Measles/Rubella - 12-23 Months
**HIV tests**	Virology-HIV tests conducted
**TB detection**	Disaggregation by Sex & Caste/Ethnicity- New TB Cases
**Diabetes visits**	Outpatient Morbidity-Nutritional & Metabolic Disorder-Diabetes Mellitus (DM) Cases
**Hypertension visits**	OPD-Morbidity-Cardiovascular & Respiratory Related Problems-Hypertension

Nepal DHIS2 definitions for primary care services.
FP – family planning
ANC – antenatal care
CBIMCI - Community Based Integrated Management of Childhood Illness
HIV – Human immunodeficiency virus
TB – Tuberculosis
OPD – outpatient department

Supplemental Table 3. Level of COVID-19 Restrictions in place from March 14, to September 16, 2020 in Nepal

	Mar 14, 2020 - April 12, 2020 (Chatira 2076)	April 13, 2020 - May 13, 2020 (Baisakh 2077)	May 14, 2020 - June 14, 2020 (Jestha 2077)	June 15, 2020 - July 15, 2020 (Ashar 2077)	July 16, 2020 - August 16, 2020 (Shrawan 2077)	August 17, 2020 - September 16, 2020 (Bhadra 2077)
Stay-at-home required (except essentials)	National[1]	National	National	National	District or palika-specific[3]	District or palika-specific
Business/workplace closures required	National[1]	National	National	National	District or palika-specific[3]	District or palika-specific
Public transport closures	National[1]	National	National	National	District or palika-specific[3]	District or palika-specific
Restricted gatherings to <10	National[1]	National	National	National	National	National
Border closure	National[1]	National	National	National	National	National
School closures	National[1]	National	National	National	National	National
Restrictions on internal movement	National[1]	National	National	National	National	National

[1]These policies were put in place on or around March 22nd, 2020.
[2]These policies were lifted on or around July 22nd, 2020.

Sources:

Rayamajhee B, Pojhrel A, Syangtan G, *et al.* How Well the Government of Nepal Is Responding to COVID-19? An Experience From a Resource-Limited Country to Confront Unprecedented Pandemic. *Front Public Health* 2021; published online Feb 17. https://doi.org/10.3389/fpubh.2021.597808.

Thomas Hale, Noam Angrist, Rafael Goldszmidt, *et al.* A global panel database of pandemic policies policies (Oxford COVID-19 Government Response Tracker). *Nature Human Behaviour* 2021. DOI:10.1038/s41562-021-01079-8.

The Situation of Corona Virus (COVID-19) in Nepal: Daily Reports. INSECOnline. http://inseconline.org/en/covid-19/.

Pradhan TR. Nepal goes under lockdown for a week starting 6am Tuesday. The Kathmandu Post. 2020; published online March 23. https://kathmandupost.com/national/2020/03/23/nepal-goes-under-lockdown-for-a-week-starting-6am-tuesday.

Pradhan TR. Government decides to lift the four-month-long coronavirus lockdown, but with conditions. The Kathmandu Post. 2020; published online July 21. https://kathmandupost.com/national/2020/07/21/government-decides-to-lift-the-four-month-long-coronavirus-lockdown-but-with-conditions.

Supplemental Table 4. Joint F-tests for parallel trends assessment

	May * Restrictions lifted & June * Restrictions lifted Joint F-test (p-value)
Contraceptive Users	0.43
ANC Visits	0.16
PNC Visits	0.46
Child pneumonia visits	0.29
Measles vaccine	0.24
Outpatient visits	0.32
Diabetes visits	0.52
Hypertension visits	0.11
HIV tests	0.86
TB cases detected	0.16

P-values for joint F-test for *May * Restrictions lifted* and *June* Restrictions lifted* to test they are jointly not significantly different from zero.
May is May 14, 2020 to June 14, 2020 (Jestha 2077) and *June* is June 15, 2020 to July 15, 2020 (Ashar 2077). May and June are in the pre-period, during the national lockdown. The coefficients for *May*Restrictions lifted* and *June*Restrictions lifted* assess if trends are parallel in the pre-period, if the effect of lifting COVID-19 restrictions in palikas is significantly different from the baseline month, April 13, 2020 to May 13, 2020 (Baisakh 2077).

Supplemental Table 5. Estimated effect of lifting COVID-19 restrictions on primary care service utilization in Nepal, estimates from difference-in-differences models that exclude March 14, 2020 – April 12, 2020 (Chaitra 2076)

	Restrictions lifted	95% CI	COVID-19 cases	95% CI	N	R^2	adj. R^2
**Contraceptive users**	56.40*	[11.63,101.17]	-0.01	[-0.03,0.01]	2968	0.01	0.01
**ANC Visits**	18.32***	[8.31,28.33]	0.00	[-0.01,0.01]	2900	0.03	0.03
**PNC Visits**	-1.06	[-4.89,2.78]	0.00	[-0.00,0.00]	2076	0.07	0.07
**Child pneumonia visits**	1.45*	[0.11,2.80]	-0.00**	[-0.00,-0.00]	2220	0.03	0.03
**Measles vaccine**	17.60*	[2.69,32.50]	-0.01**	[-0.01,-0.00]	1392	0.08	0.07
**Outpatient visits**	-84.61	[-223.74,54.53]	-0.10***	[-0.13,-0.06]	2968	0.10	0.10
**Diabetes visits**	3.61	[-8.74,15.95]	0.00	[-0.01,0.02]	1332	0.02	0.02
**Hypertension visits**	11.95	[-7.68,31.58]	0.00	[-0.01,0.01]	2756	0.02	0.01
**HIV tests**	33.24	[-11.71,78.20]	0.01	[-0.01,0.03]	944	0.04	0.03
**TB cases detected**	-0.02	[-0.83,0.78]	0.00	[-0.00,0.00]	920	0.05	0.05

95% confidence intervals in brackets
* $p < 0.05$, ** $p < 0.01$, *** $p < 0.001$
The coefficient for *Restrictions lifted* is the effect of lifting COVID-19 restrictions on health service utilization. Models also included fixed effects for month and palikas.

Kapoor NR, *et al. BMJ Open* 2022; 12:e061849. doi: 10.1136/bmjopen-2022-061849

Supplemental Table 6. Number of palika and volume of services in the raw vs. final dataset

		Number of palika reporting			Sum of services		
**Health service**	**Variable name**	**Raw data**	**Final data**	**% difference**	**Raw data**	**Final data**	**% difference**
Contraceptive users	fp_sa_util	753	742	-1%	2,702,347	2,685,227	-1%
Antenatal care	anc_util	752	725	-4%	276,465	271,893	-2%
Postnatal care visits	pnc_util	717	519	-28%	46,846	43,617	-7%
Child pneumonia visits	pneum_util	745	555	-26%	27,164	25,030	-8%
Outpatient visits	opd_util	753	742	-1%	6,866,933	6,828,125	-1%
TB cases detected	tbdetect_qual	679	230	-66%	6,572	4,577	-30%
Measles Vaccine	measles_qual	753	348	-54%	361,213	210,662	-42%
Hypertension visits	hyper_util	752	689	-8%	222,659	220,033	-1%
Diabetes visits	diab_util	627	333	-47%	93,261	89,390	-4%
HIV tests	hivtest_qual	520	236	-55%	227,166	213,789	-6%

Kapoor NR, *et al. BMJ Open* 2022; 12:e061849. doi: 10.1136/bmjopen-2022-061849
