# Supplementary material for: Effect of lifting COVID-19 restrictions on utilisation of primary care services in Nepal: a difference-in-differences analysis
Source: BMJ Open. 2022 Nov 29;12(11):e061849. doi: 10.1136/bmjopen-2022-061849 (PMC9709811; doi:10.1136/bmjopen-2022-061849)
Supplement: Author's manuscript [file bmjopen-2022-061849.draft_revisions.pdf]

BMJ Open is committed to open peer review. As part of this commitment we make the peer review history of every article we publish publicly available.

When an article is published we post the peer reviewers' comments and the authors' responses online. We also post the versions of the paper that were used during peer review. These are the versions that the peer review comments apply to.

The versions of the paper that follow are the versions that were submitted during the peer review process. They are not the versions of record or the final published versions. They should not be cited or distributed as the published version of this manuscript.

BMJ Open is an open access journal and the full, final, typeset and author-corrected version of record of the manuscript is available on our site with no access controls, subscription charges or pay-per-view fees (http://bmjopen.bmj.com).

If you have any questions on BMJ Open's open peer review process please email info.bmjopen@bmj.com

BMJ Open

The effect of lifting COVID-19 restrictions on utilization of primary care services in Nepal: a difference-in-differences analysis

Journal:	*BMJ Open*
Manuscript ID	bmjopen-2022-061849
Article Type:	Original research
Date Submitted by the Author:	08-Feb-2022

Complete List of Authors:	Kapoor, Neena ; Harvard University T H Chan School of Public Health, Department of Global Health and Population<br>Aryal, Amit; Swiss Tropical and Public Health Institute<br>Mehata, Suresh; Government of Nepal Ministry of Health and Population<br>Dulal, Mahesh; Office of Member of Federal Parliament Nepal, Gagan Kumar Thapa<br>Kruk, Margaret; Harvard University T H Chan School of Public Health, Department of Global Health and Population<br>Bauhoff, Sebastian; Harvard University T H Chan School of Public Health, Department of Global Health and Population<br>Arsenault, C; Harvard University T H Chan School of Public Health, Department of Global Health and Population

Keywords:	COVID-19, Health policy < HEALTH SERVICES ADMINISTRATION & MANAGEMENT, PRIMARY CARE

SCHOLARONE™
Manuscripts

I, the Submitting Author has the right to grant and does grant on behalf of all authors of the Work (as defined in the below author licence), an exclusive licence and/or a non-exclusive licence for contributions from authors who are: i) UK Crown employees; ii) where BMJ has agreed a CC-BY licence shall apply, and/or iii) in accordance with the terms applicable for US Federal Government officers or employees acting as part of their official duties; on a worldwide, perpetual, irrevocable, royalty-free basis to BMJ Publishing Group Ltd ("BMJ") its licensees and where the relevant Journal is co-owned by BMJ to the co-owners of the Journal, to publish the Work in this journal and any other BMJ products and to exploit all rights, as set out in our licence.

The Submitting Author accepts and understands that any supply made under these terms is made by BMJ to the Submitting Author unless you are acting as an employee on behalf of your employer or a postgraduate student of an affiliated institution which is paying any applicable article publishing charge ("APC") for Open Access articles. Where the Submitting Author wishes to make the Work available on an Open Access basis (and intends to pay the relevant APC), the terms of reuse of such Open Access shall be governed by a Creative Commons licence – details of these licences and which Creative Commons licence will apply to this Work are set out in our licence referred to above.

Other than as permitted in any relevant BMJ Author's Self Archiving Policies, I confirm this Work has not been accepted for publication elsewhere, is not being considered for publication elsewhere and does not duplicate material already published. I confirm all authors consent to publication of this Work and authorise the granting of this licence.

For peer review only

The effect of lifting COVID-19 restrictions on utilization of primary care services in Nepal: a difference-in-differences analysis

Authors: Kapoor NR[1*], Aryal A[2], Mehata S[3], Dulal M[4], Kruk ME[1], Bauhoff S[1], Arsenault C[1]

1. Department of Global Health and Population, Harvard T.H. Chan School of Public Health, Boston, USA
2. Swiss Tropical and Public Health Institute, University of Basel, Basel, Switzerland
3. Ministry of Health and Population, Government of Nepal, Kathmandu, Nepal
4. Office of Member of Federal Parliament Nepal, Gagan Kumar Thapa, Kathmandu, Nepal

***Corresponding Author:**
655 Huntington Ave, Boston MA, USA
nkapoor@hsph.harvard.edu

For peer review only

For peer review only - http://bmjopen.bmj.com/site/about/guidelines.xhtml

Abstract

Introduction An increasing number of studies have reported disruptions in health service utilization due to the COVID pandemic and its associated restrictions. However, little is known about the effect of lifting COVID restrictions on health service utilization. The objective of this research was to estimate the effect of lifting COVID restrictions on primary care service utilization in Nepal.

Methods Data on utilization of 10 primary care services were extracted from the Health Management Information System (HMIS) across all of Nepal. We used a difference-in-differences design and linear fixed effects regressions to estimate the effect of lifting COVID restrictions. The treatment group included palikas that had no restrictions in August 17 to September 16, 2020 and the control group included palikas that maintained restrictions during that period. The pre-period included the four months of national lockdown in Nepal from March 24 to July 22, 2020. Models included month and palika fixed effects and controlled for COVID incidence.

Results We found that lifting COVID restrictions was associated with an average increase per palika of 57.5 contraceptive users (95% CI 14.6, 100.5), 15.6 antenatal care visits (95% CI 5.3, 25.9), and 1.6 child pneumonia visits (95% CI 0.2, 2.9). This corresponded to a 9.4% increase in contraception, 34.2% increase in antenatal care visits and 15.6% increase in child pneumonia visits. Utilization of most other primary care services also increased after lifting restrictions, but coefficients were not statistically significant.

Conclusions Despite the ongoing pandemic, lifting restrictions can lead to an increase in reproductive, maternal and child health service utilization. Our results call for policy makers in low- and middle-income countries to carefully consider the tradeoffs of strict lockdowns during future COVID waves or future pandemics and prepare health systems for potential rebounds in service utilization as restrictions are lifted.

For peer review only

Key questions

What is already known on this topic?

- Disruptions in primary care services have been experienced across several countries since the start of the COVID-19 pandemic.
- To our knowledge, there is no empirical evidence on the effect of lifting COVID-19 restrictions on primary health service utilization in low-and-middle income countries.

What are the new findings?

- On July 22, 2020, COVID restrictions were lifted in some palikas (municipalities) in Nepal but not others, giving rise to a natural experiment.
- Using health management information systems data from all of Nepal and a difference-in-differences design, we assessed the effect of lifting COVID-19 restrictions on primary healthcare services.
- Across 10 services studied, we found that lifting COVID-19 restrictions led to an increase in contraceptive use, antenatal care visits, and sick child visits for pneumonia. Utilization of most other primary care services also increased, but effect estimates were not significantly different from zero.

What do the new findings imply?

- These results call for policy makers to prepare health systems for potential rebounds in health services when COVID-19 restrictions are lifted.
- Reproductive, maternal and child health services may be more resilient than other types of services in this context.

For peer review only

Background

In a time of crisis, high quality health systems have two tasks: respond to the crisis and maintain the provision and quality of essential health services.[1] Health systems in low-income countries, that may already be under-funded, under-resourced, and over-burdened, may be particularly vulnerable during the COVID pandemic. An increasing number of studies have reported disruptions in health service utilization since the start of the pandemic in low- and middle-income countries (LMICs).[2,3,4,5,6,7,8,9,10] The ongoing COVID pandemic has directly strained health care systems around the world that are struggling to meet the physical resource, human resource (numbers and skills), and service coordination demands of the pandemic. In addition, the pandemic had indirect effects on primary health care utilization, as restrictions and lockdowns implemented by governments to reduce the spread of COVID could also affect people's ability or willingness to visit healthcare facilities for routine primary care.

Nepal is a lower-middle income country of South Asia with a population of 28.6 million.[11] The country has shown significant gains in health and healthcare utilization over the past decade that are at risk of being reversed by the pandemic. As of December 2021, more than 800,000 people have been infected with COVID across Nepal and a reported 11,594 people have died from COVID.[12] Despite the existence of effective vaccines, only 32.8% of the Nepali population is currently fully vaccinated against COVID.[12]

Following the declaration of the pandemic on March 11, 2020 by the World Health Organization, health care utilization declined substantially in Nepal, ranging from a 65% decline in tuberculosis (TB) case detection to a 4% decline in contraceptive use.[3] Declines in health service use may stem from the declaration of the pandemic itself (perceived threat), the actual number of new COVID cases reported in a given period (leading to a fear of infection when visiting facilities or to overburdened health facilities treating COVID patients) or from the restrictions imposed (i.e., lockdowns) to curb the spread of COVID. The barriers imposed by COVID restrictions (e.g., stay-at home requirements or public transport closures) may play an important role in decreasing health care utilization. In Madesh Pradesh (formerly known as Province 2) of Nepal, people reported that the national lockdown restricted accessibility to health facilities and deterred them from seeking care.[13] An increasing number of studies have described the effects of the pandemic and associated restrictions on health care utilization.[2,3,4,5,6,7,8,9,10] However, little is known about the effect of lifting COVID restrictions on health care utilization. Understanding these effects is crucial to plan for potential rebounds in demand and determining whether potentially weakened health systems can cope with surges in demand.

On March 24, 2020, the Government of Nepal implemented a country-wide lockdown with strict restrictions on movement of people and closure of non-essential businesses.[14,15] On July 22, 2020, the decision was made to lift most of these restrictions at the national level and end the four-month lockdown.[16] However, some of Nepal's 77 districts and 753 municipality governments (*palikas, a local of government in Nepal's federal system)* decided to maintain restrictions to contain the spread of COVID. This contrast in removal of restrictions gave rise to a natural experiment that allowed us to estimate the causal effect of lifting COVID restrictions on health care utilization.

For peer review only - http://bmjopen.bmj.com/site/about/guidelines.xhtml

In this study, we used a difference-in-differences design (DID) to estimate the effect of lifting COVID restrictions on primary care service utilization. Understanding the effect of lifting COVID restrictions on primary healthcare is crucial to inform policy responses during future waves of COVID or future pandemics in low- and middle-income countries.

For peer review only

For peer review only - http://bmjopen.bmj.com/site/about/guidelines.xhtml

Methods

Study groups and periods

From March 24 to July 22, 2020, the Federal Government of Nepal imposed a strict nation-wide lockdown in response to the pandemic. This included stay-at-home requirements except for essential services, businesses, public transport and school closures, and restrictions on large gatherings, international travel, and internal movement (see **supplementary material table 2**). On July 22, the major restrictions were lifted at the national level (including stay-at-home requirements, non-essential business, and public transport closures) but some districts and palikas maintained these restrictions. Following the lifting of the national lockdown, 248 palikas lifted the restrictions while 505 palikas maintained one or more of these restrictions.

For this analysis, the treatment group includes the palikas that lifted the restrictions, while the control group includes palikas that continued at least one or more restriction. The pre-intervention period includes the four months from March 14 to July 15 (which corresponds to the Nepali months of Chaitra 2076 to Ashar 2077) and the post-intervention period is August 17 to September 16, 2020 (the Nepali month of Bhadra 2077). Our analysis used Nepal calendar months as the unit of time. Palikas were included in the control group (palikas with restrictions) if a restriction was in place for at least 10 days of the month.

Figure 1 shows the timeline of COVID restrictions and cases in Nepal from January 1, 2020, to September 16, 2020. The first COVID case was reported in Nepal on January 25, 2020. Notably, the end of the national lockdown on July 22, 2020 coincided with the beginning of the first real COVID wave (**figure 1**).

Data sources and measures

We obtained the monthly number of primary care services from the Nepal Health Management Information System (HMIS) through the DHIS2 platform, following the Nepali calendar for the period of Magh 2075 to Poush 2077 (equivalent to January 15, 2019, to January 13, 2021). These data were available at the palika level (a local level of government in Nepal, currently 753 palikas in Nepal). A total of 7,605 health facilities are expected to report to the DHIS2 across these 753 palikas.

We aimed to include 12 primary care services: contraceptive users, antenatal care (ANC) visits, postnatal care (PNC) visits, visits for children under five with pneumonia, visits for children under five with diarrhea, pentavalent vaccinations, measles vaccinations, visits for diabetes, visits for hypertension, number of human immunodeficiency virus (HIV) tests conducted, number of tuberculosis (TB) cases detected and total outpatient visits. Detailed definitions are in **supplementary material table 1**. Because DHIS2 data are self-reported by health facilities, these data may contain errors. Our data cleaning procedures entailed identifying positive outliers (greater than 3.5 standard deviations from the mean trend) and setting any outliers as missing.[17] We did not assess negative outliers since decreases in utilization were expected during the lockdown period. For each health service, we also excluded palikas that were missing any data during the five-month study period (a complete case analysis). We compared the resulting dataset to the raw data prior to cleaning to ensure the final dataset was still representative.

For peer review only - http://bmjopen.bmj.com/site/about/guidelines.xhtml

To determine which palika had maintained restrictions after July 22, 2020, we used data from INSECOnline, a human rights news portal in Nepal providing daily COVID updates.[18] These data were validated using the Nepal COVID Crisis Management Coordination Center (CCMCC) government websites, additional online news sources and they were reviewed by local researchers. The analysis also included data on the total number of COVID cases at district level in Nepal (COVID case count was not available at the palika level). Monthly COVID cases in each of the 77 districts were obtained from the Nepal Health Emergency Operation Centre, Ministry of Health and Population.[19]

Statistical analysis

The analysis was conducted at the palika level, using Nepali calendar months as the unit of time. We used a DID design and fixed effects ordinary least square regression models. The following model was used and repeated for each of the 10 health service analyzed:

$$S_{pt} = \alpha + \beta[lifted\ lockdown_{pt}] + \gamma_t + \ \delta_p + X_{dt} + \varepsilon_{pt}$$

Where S_{pt} is the number of health services (number of visits or users) provided in palika p in month t, γ_t and δ_p are vectors of month and palika fixed effects, respectively, and X_{dt} is the number of new COVID cases in district d and month t. The coefficient of interest is β, which represents the difference in service utilization among palikas that lifted restrictions compared to those that maintained restrictions. The palika fixed effects controls for time-invariant differences between palikas and avoids the need to control for time-fixed confounders. For example, the palika fixed effects will control for unmeasured differences between palikas (urbanicity, population size, wealth) that can affect service utilization. The DID design also controls for all factors commonly affecting the outcomes in all palikas over time, through month fixed effects. We included the number of COVID cases as a potential time-varying confounder as COVID incidence would be associated with both the exposure (restrictions), and the outcome (health service utilization) and may vary between the treatment and control groups. Thus, we included monthly COVID cases in the regression models to control for potential confounding. Models also included clustered standard errors at the palika level.

A main assumption of DID models is that the outcome trend in the control group represents a good approximation of what the outcome trend would have been in the treatment group in the absence of the policy change (i.e., the counterfactual trend). Thus, to probe the assumption that the control palika trends were a good counterfactual for the treatment group (palikas that lifted restrictions), we implemented a series of tests. First, we conducted a pre-trend placebo test by comparing the difference in service utilization between the treated and control palikas in May and June 2020 (Jestha and Ashar 2077) compared to April 2020 (Baisakh 2077). We performed a joint F test of whether these coefficients were jointly zero (**supplementary materials**). Since all palikas were under the same lockdown in March to June 2020 (Ashar 2077), there should be no effect, providing evidence for parallel trends in the pre-period. Second, we assessed the parallel trend assumption graphically (**figure 2**). We also conducted a sensitivity analysis, excluding March 14 to April 12, 2020 (Chaitra 2076) from the analysis, since the national lockdown was put in place in the middle of this month, to see if the results differed. This research was approved by the Nepal Health Research Council (NHRC), reference number 650, and determined to be

For peer review only - http://bmjopen.bmj.com/site/about/guidelines.xhtml

exempt from a full review by the Institutional Review Board (IRB) of the Harvard T.H. Chan School of Public Health.

Patient and public involvement

Patients will be involved in dissemination of this research. There was no patient or public involvement in the design, reporting, or interpretation of results.

For peer review only

For peer review only - http://bmjopen.bmj.com/site/about/guidelines.xhtml

Results

Table 1 shows the average number of services provided during the national lockdown (pre-intervention period) and after the national lockdown was lifted (post-intervention period) for treated and control palikas. It also shows the number of palikas reporting each health service. While service utilization tended to be lower in the treatment group for a majority of services, these differences are accounted for by the palika fixed effects in the DID design.

Table 1: Average number of health care visits per month and number of palikas reporting each service by treatment group and period

	COVID restrictions in place Nationally (pre-period)		COVID restrictions lifted in some districts/palikas (post-period)	
	Treatment group Average per month (N)	Control group Average per month (N)	Treatment group Average per month (N)	Control group Average per month (N)
**Contraceptive users**	614.09 (245)	780.52 (497)	646.10 (245)	751.41 (497)
**Antenatal care visits**	45.66 (235)	90.57 (490)	48.97 (235)	81.52 (490)
**Postnatal care visits**	12.49 (182)	16.93 (337)	18.65 (182)	24.63 (337)
**Child pneumonia visits**	9.92 (186)	8.59 (369)	10.98 (186)	7.95 (369)
**Measles Vaccine**	84.47 (102)	140.83 (246)	75.28 (102)	121.74 (246)
**Outpatient visits**	1489.23 (243)	1896.25 (499)	1870.75 (243)	2286.77 (499)
**Diabetes visits**	46.66 (100)	54.27 (233)	57.04 (100)	62.00 (233)
**Hypertension visits**	53.43 (220)	65.50 (469)	72.44 (220)	72.93 (469)
**HIV tests**	87.55 (68)	212.92 (168)	125.88 (68)	228.17 (168)
**TB cases detected**	3.07 (52)	4.25 (178)	3.13 (52)	4.20 (178)

The average number of health visits per month. The treatment group includes the palikas that lifted lockdowns in Bhadra 2077 (August 17 to September 16, 2020). The control group includes those that maintained lockdowns during that month. The (N) is the number of palikas reporting each month in the period for that service. National lockdown period (pre-period) includes Chaitra 2076 to Ashar 2077 (March 13 to July 16, 2020). The post-period includes Bhadra 2077 (August 17 to September 16, 2020).

Figure 2 shows the trend in primary care service utilization from January 2019 to September 2020 (equivalent to Magh 2075 to Bhadra 2077) and reveal parallel trends before and during the national lockdown period (our pre-period) for all services included. The control palikas thus appear to provide appropriate counterfactual trends in the post-period. The joint F-test (available in the **supplementary materials**) also did not reject the null hypothesis that the outcomes evolved differently in treated vs. control palikas in the pre-period for the 10 services included. The parallel trend assumption was violated for two health services: visits for children under five with diarrhea and pentavalent vaccinations, which were excluded from the analysis.

Table 2. Estimated effect of lifting COVID restrictions on primary care service utilization in Nepal, estimates from difference-in-differences models

For peer review only - http://bmjopen.bmj.com/site/about/guidelines.xhtml

	Restrictions lifted	95% CI	COVID cases	95% CI	N	R^2	adj. R^2
**Contraceptive users**	57.51**	[14.55,100.48]	-0.01	[-0.02,0.01]	3710	0.01	0.01
**ANC visits**	15.60**	[5.34,25.86]	0.01	[-0.01,0.02]	3625	0.08	0.07
**PNC visits**	-1.50	[-4.94,1.94]	0.00	[-0.00,0.00]	2595	0.07	0.07
**Child pneumonia visits**	1.55*	[0.24,2.86]	-0.00**	[-0.00,-0.00]	2775	0.19	0.19
**Measles vaccine**	7.35	[-6.49,21.19]	0.00	[-0.01,0.00]	1740	0.20	0.20
**Outpatient visits**	-56.81	[-193.77,80.16]	-0.10***	[-0.13,-0.06]	3710	0.09	0.09
**Diabetes visits**	5.01	[-8.12,18.14]	0.00	[-0.01,0.02]	1665	0.03	0.02
**Hypertension visits**	12.70	[-6.74,32.14]	0.00	[-0.01,0.01]	3445	0.02	0.02
**HIV tests**	34.83	[-12.57,82.22]	0.02	[-0.02,0.05]	1180	0.04	0.04
**TB cases detected**	0.06	[-0.73,0.85]	0.00	[-0.00,0.00]	1150	0.04	0.04

95% confidence intervals in brackets
* $p < 0.05$, ** $p < 0.01$, *** $p < 0.001$
The coefficient for *Restrictions lifted* is the effect of lifting COVID restrictions on health service utilization. Models also included fixed effects for month and palika.

Estimates from DID regressions are reported in **Table 2** for the 10 health services. The coefficient for *restrictions lifted* is the DID estimate and can be interpreted as the difference in adjusted service utilization between the treatment (lifted restrictions) and control (maintained restrictions) palikas in the post-period.

Lifting COVID restrictions led to a positive increase in all services except total outpatient visits and postnatal care visits. These effects were statistically significant for three services. Lifting restrictions led to an average increase per palika of 57.5 contraceptive users (95% Confidence Interval (CI) 14.6-100.5), 15.6 antenatal care visits (95% CI 5.3-25.9), and 1.6 child pneumonia visits (95% CI 0.2-2.9). Compared to the pre-COVID average utilization, this represented a 9.4% increase in contraceptive use, 34.2% increase in antenatal care visits and a 15.6% increase in child pneumonia visits.

Similarly, although not statistically significant, lifting restrictions led to 7.4 more children vaccinated against measles (95% CI -6.5-21.2), 5.0 more diabetes visits (95% CI -8.1-18.1), 12.7 more hypertension visits (95% CI -6.7-32.1), 34.8 additional HIV tests (95% CI -12.6-82.2) and 0.1 additional TB cases detected (95% -0.7-0.9) on average per palika. These increases were equivalent to increases of 8.7% for measles vaccinations, 10.7% for diabetes visits, 23.8% for hypertension visits, 39.8% for HIV tests, and 2.0% for TB case detection in palikas that lifted restrictions compared to those that maintained it. In contrast, the coefficient for PNC and total outpatient visits were negative but these were not statistically significant. They were equivalent to declines of 12.0% fewer PNC visits and 3.8% fewer outpatient visits in palikas that lifted restrictions compared to those that didn't. Results from the sensitivity analysis that excluded March 14 to April 12, 2020 (Chaitra 2076) were largely consistent with the main model, for the exception of measles vaccinations which had a statistically significant increase in palikas that lifted restrictions (see **supplementary material table 3**).

For peer review only - http://bmjopen.bmj.com/site/about/guidelines.xhtml

Discussion

In this analysis, we used national-level HMIS data and a differences-in-differences design to estimate the causal effect of lifting COVID restrictions on primary care health service utilization in Nepal. We found that lifting restrictions increased contraceptive use, antenatal care, and sick child visits by 9.4% to 34.2% on average across palikas. Utilization of most other primary care services also increased by 2.0% to 39.8% but were not statistically significant. These results provide evidence that COVID restrictions are causally linked to primary care service utilization in Nepal and that lifting these restrictions can lead to a rebound in service provision. To our knowledge, this is the first paper to estimate the effect of lifting COVID restrictions on health service utilization.

There are many mechanisms through which COVID-related restrictions (stay-at-home requirements, business/workplace closures and public transport closures) might affect primary healthcare utilization. People in Nepal have reported that public transport closures during the national lockdown prevented them from reaching healthcare facilities.[13] In addition, stay-at-home requirements meant that people were only permitted to leave their home for essential services. Though essential services included healthcare, stay-at-home requirements were firmly enforced by law enforcement officials, and individuals were arrested and jailed or fined if they defied them.[14,20] Knowing this risk, people may have been deterred from seeking healthcare, despite being allowed. In addition, the strict lockdown may have increased anxieties around COVID which deterred people from seeking care.[13,21] It is also possible that many people did not know that visiting health facilities was allowed during the lockdown. Once COVID restrictions were lifted, these barriers would be subdued, and an increase in utilization would be expected.

Lockdown policies, primarily stay-at-home requirements and business or workplace closures, can also have detrimental economic effects, potentially pushing low-income individuals and families further into poverty.[14,22] Most people in Nepal work in the informal sector, including a large number in the tourism industry, which was severely impacted by the pandemic. Although the government developed economic support packages, informal sector workers or other marginalized groups were often missed due to difficulties in implementation.[14] Essential health services are supposed to be free of charge in public facilities in Nepal. [23] However, Nepalis often incur costs at point of service, with more than half of health expenditures being made out-of-pocket.[23,24] In addition, due to stockouts and staff shortages in public facilities, among other reasons, between 20% and 61% of people choose private sector facilities for primary care in Nepal, depending on the type of service.[23,25,26] Lifting COVID restrictions could increase households' ability to generate income, especially for those working in the informal sector and may have allowed them to pay for health care costs.

Lifting COVID restrictions had statistically significant effects only for reproductive, maternal and child health services (RMNCH): contraceptives, antenatal care, and child pneumonia visits. It is possible that these services could be more resilient than other types of primary care services and that the population understands the importance of these services better. The government of Nepal has focused on improving and maintaining sexual, reproductive, maternal, newborn, child and adolescent health (SRMNCAH), both historically and during the pandemic, which could

For peer review only - http://bmjopen.bmj.com/site/about/guidelines.xhtml

explain why they significantly increased after restrictions were lifted, in comparison to other primary care services.[27] We found no impact of lifting COVID restrictions on PNC visits. This could be due to a large program for postnatal care home visits and outreach which was launched shortly before the pandemic and continued during the lockdown in some districts. This could explain why there were no differences between palikas that lifted restrictions and those that maintained them.

Other studies have found declines in health service utilization of various magnitude and duration following the declaration of the pandemic and the implementation of restrictions in many countries.[2,3,4,5,6,7,8,9,10] Studies from Nepal found declines in primary care and hospital-based care during the pandemic including fewer deliveries and a potential increase in neonatal mortality and institutional stillbirths.[3,8] In contrast, we assessed the effect of lifting restrictions, and the resulting increases in service use. Our DID design estimates the causal effect of lifting COVID restrictions. DID compares trends between the treatment and comparison groups and compares each unit to itself, estimating an average of the counterfactual DID contrasts.

Nonetheless, our study has limitations. The DID design controls for time fixed differences between palikas and for secular trends affecting all groups. However, it is possible that remaining time-varying confounders affected the two groups differently. For example, although we adjusted for COVID caseloads at the district level, it is possible that palika-specific outbreaks influenced the decision to maintain restrictions. Another potential concern relates to measurement error for both the policies and the health service utilization outcomes. The policy data (whether a palika had restrictions in place or not) may have been misclassified due to missing data, although multiple sources were reviewed to collect and confirm them. Any misclassification due to missing data would most likely lead to the palika being included in the treatment group (lifted restrictions) when they actually maintained restrictions. This would bias the results towards the null. DHIS2 data may also contain errors, and reporting quality may have been affected by the pandemic. However, positive outliers were removed and only facilities that reported each indicator completely every month over the study period were included. It is unclear whether DHIS2 data quality issues would affect our analysis since misreporting should be similar in both the treatment and control groups. Finally, the outcome data was only available monthly, and the beginning and end of restrictions did not always match DHIS2 data precisely. Thus, policy dates and outcomes were not perfectly matched. In our dataset, the pre-period begins 10 days before the national lockdown. However, sensitivity analyses that excludes the first month of the lockdown shows similar results (**supplementary material table 3**).

Our results have important implications for policy. We found that despite the ongoing COVID pandemic, lifting restrictions can lead to an increase in RMNCH service utilization. Universal utilization of these services is crucial to improve health outcomes. Antenatal care visits are essential to identify conditions that might threaten the mother or newborn's health.[28] It is estimated that a 10% decrease in coverage of pregnancy related and newborn health care during COVID-19 could result in an additional 28,000 maternal deaths and 168,000 neonatal deaths globally.[29] In addition, reduced contraceptive use could results in more unintended pregnancies which can also place both the pregnant person and child at risk.[30] Finally, pneumonia is one of the leading causes of death for children under five, and missed care could further exacerbate this burden.[31] Nonetheless, it is important to note that an increase in child pneumonia visits after

For peer review only - http://bmjopen.bmj.com/site/about/guidelines.xhtml

restrictions were lifted could be linked to an increase in health needs from further spread of respiratory illnesses rather than a pent up demand.

Although effective vaccines are now available, few people in LMICs are fully immunized against COVID due to widespread inequities in access to vaccines.[32] Future waves of COVID infections and emerging variants are likely to push governments to consider re-implementing temporary restrictions and lockdowns. At the start of the pandemic, many countries took a one-size-fits-all approach with COVID containment policies, as there was understandably much uncertainty surrounding COVID and its effects. As we gain insight into the indirect effects of these restrictions, it is important that policy-makers tailor these policies to their own demographic, disease, and sociocultural contexts, and prepare health systems to respond accordingly.[22] Policy makers should consider strategies to promote and maintain all types of primary care services during future waves of COVID and future pandemics. Such strategies may include better risk communication on the importance of essential health care and alternative service delivery modes such as telemedicine or differentiated service delivery strategies.[33] Health facilities should also be prepared to face surges in demand for health care when restrictions are eased.

Contribution statement
NRK, CA, SB and MEK designed the study. NRK, CA, SM, AA and MD compiled and verified the data. NRK and CA led the data analysis. NRK wrote the first draft and all other co-authors, contributed to the interpretation of findings and read, improved and approved the final manuscript.

Funding
This work is part of the Grand Challenges ICODA pilot initiative, delivered by Health Data Research UK and funded by the Bill & Melinda Gates Foundation and the Minderoo Foundation (reference number 2021.0091). We also acknowledge funding from the Bill and Melinda Gates Foundation (grant INV-005254).

Data availability
Data may be obtained from a third party and are not publicly available.

Competing interest
There are no competing interests for any authors.

For peer review only

References:

1 Kruk, M. E., Gage, A. D., Arsenault, C., *et al.* High-quality health systems in the Sustainable Development Goals era: time for a revolution. *Lancet Glob Health* 2018; **6**: e1196–252.

2 Moynihan, R., Sanders, S., Michaleff, Z. A., *et al.* Impact of COVID-19 pandemic on utilisation of healthcare services: a systematic review. *BMJ Open* 2021; **11**. DOI:10.1136/bmjopen-2020-045343.

3 Arsenault C, Gage A, Kim M, *et al.* Effects of the COVID-19 pandemic on health care utilization; initial health system shock in 10 countries. *Manuscript submitted for publication*.

4 Hategeka, C., Carter, S. E., Chenge, F. M., *et al.* Impact of the COVID-19 pandemic and response on the utilisation of health services in public facilities during the first wave in Kinshasa, the Democratic Republic of the Congo. *BMJ Glob Health* 2021; **6**. https://www.ncbi.nlm.nih.gov/pubmed/34315776.

5 Doubova, S. V., Leslie, H. H., Kruk, M. E., Perez-Cuevas, R., Arsenault, C. Disruption in essential health services in Mexico during COVID-19: an interrupted time series analysis of health information system data. *BMJ Glob Health*; **6**. DOI:10.1136/bmjgh-2021-006204.

6 Burt, J. F., Ouma, J., Lubyayi, L., *et al.* Indirect effects of COVID-19 on maternal, neonatal, child, sexual and reproductive health services in Kampala, Uganda. *BMJ Glob Health* 2021; **6**. DOI:10.1136/bmjgh-2021-006102.

7 Aranda Z, Binde T, Tashman K, *et al.* Disruptions in maternal health service use during the COVID-19 pandemic in 2020: experiences from 37 health facilities in low-income and middle-income countries. *BMJ Global Health* 2022; **7**: e007247.

8 KC A, Gurung R, Kinney MV, *et al.* Effect of the COVID-19 pandemic response on intrapartum care, stillbirth, and neonatal mortality outcomes in Nepal: a prospective observational study. *The Lancet Global Health* 2020; **8**: e1273–81.

9 Shapira G, Ahmed T, Drouard SHP, *et al.* Disruptions in maternal and child health service utilization during COVID-19: analysis from eight sub-Saharan African countries. *Health Policy Plan* 2021; **36**: 1140–51.

10 Ahmed T, Roberton T, Team M of EHS, *et al.* Indirect Effects on Maternal and Child Mortality from the COVID-19 Pandemic: Evidence from Disruptions in Healthcare Utilization in 18 Low- and Middle-Income Countries. Rochester, NY: Social Science Research Network, 2021 DOI:10.2139/ssrn.3916767.

11 The World Bank Data. 2019 https://data.worldbank.org/country/nepal.

12 Our World in Data. 2021 https://ourworldindata.org/coronavirus/country/nepal?country=~NPL.

For peer review only - http://bmjopen.bmj.com/site/about/guidelines.xhtml

13 Singh, D. R., Sunuwar, D. R., Shah, S. K., *et al.* Impact of COVID-19 on health services utilization in Province-2 of Nepal: a qualitative study among community members and stakeholders. *BMC Health Serv Res* 2021; **21**: 174.

14 Rayamajhee B, Pojhrel A, Syangtan G, *et al.* How Well the Government of Nepal Is Responding to COVID-19? An Experience From a Resource-Limited Country to Confront Unprecedented Pandemic. *Front Public Health* 2021; published online Feb 17. https://doi.org/10.3389/fpubh.2021.597808.

15 Pradhan TR. Nepal goes under lockdown for a week starting 6am Tuesday. The Kathmandu Post. 2020; published online March 23. https://kathmandupost.com/national/2020/03/23/nepal-goes-under-lockdown-for-a-week-starting-6am-tuesday.

16 Pradhan TR. Government decides to lift the four-month-long coronavirus lockdown, but with conditions. The Kathmandu Post. 2020; published online July 21. https://kathmandupost.com/national/2020/07/21/government-decides-to-lift-the-four-month-long-coronavirus-lockdown-but-with-conditions.

17 Module 2: Desk review of data quality. World Health Organization, 2017.

18 The Situation of Corona Virus (COVID-19) in Nepal: Daily Reports. INSECOnline. http://inseconline.org/en/covid-19/.

19 CoVid-19 Dasboard. Government of Nepal, Ministry of Health and Population https://covid19.mohp.gov.np/ (accessed Feb 9, 2021).

20 Nepal Police crack down on 749 Kathmandu residents for violating Covid-19 lockdown. India Today. 2020; published online March 30. https://www.indiatoday.in/world/story/nepal-police-crackdown-on-749-kathmandu-residents-for-violating-covid-19-lockdown-1661343-2020-03-30.

21 Yadav UN, Yadav OP, Singh DR, *et al.* Perceived fear of COVID-19 and its associated factors among Nepalese older adults in eastern Nepal: A cross-sectional study. *PLOS ONE* 2021; **16**: e0254825.

22 Cash R, Patel V. Has COVID-19 subverted global health? *Lancet* 2020; **395**: 1687–8.

23 Free Health Care in Nepal: Findings of a Rapid Assessment. GFA Consulting Group https://files.givewell.org/files/DWDA%202009/Nyaya/en-free-health-care-nepal.pdf.

24 Kalaunee SP. Is free health care possible in Nepal? Nepali Times. 2019; published online Dec 12. https://www.nepalitimes.com/opinion/is-free-health-care-possible-in-nepal/ (accessed Dec 3, 2021).

25 Arsenault C, Kim MK, Aryal A, *et al.* Hospital-provision of essential primary care in 56 countries: determinants and quality. *Bull World Health Organ* 2020; **98**: 735-746D.

For peer review only - http://bmjopen.bmj.com/site/about/guidelines.xhtml

26 Acharya S, Ghimire S, Jeffers EM, Shrestha N. Health Care Utilization and Health Care Expenditure of Nepali Older Adults. *Front Public Health* 2019; **7**: 24.

27 Responding to COVID-19: Health sector preparedness, response and lessons learnt. Kathmandu, Nepal: Government of Nepal, Ministry of Health and Population, 2021.

28 Banta D. What is the efficacy/effectiveness of antenatal care and the financial and organizational implications? Copenhagen: WHO Regional Office for Europe, 2003 https://www.euro.who.int/__data/assets/pdf_file/0007/74662/E82996.pdf.

29 Riley, Sully, Ahmed, Biddlecom. Estimates of the Potential Impact of the COVID-19 Pandemic on Sexual and Reproductive Health In Low- and Middle-Income Countries. *International Perspectives on Sexual and Reproductive Health* 2020; **46**: 73.

30 Weigel G, Salganicoff A, Ranji U. Potential Impacts of Delaying "Non-Essential" Reproductive Health Care. Kaiser Family Foundation, 2020.

31 Bernadeta Dadonaite. Pneumonia — no child should die from a disease we can prevent. 2019. https://ourworldindata.org/child-deaths-from-pneumonia.

32 Rouw A, Wexler A, Kates J, Michaud J. Global COVID-19 Vaccine Access: A Snapshot of Inequality. Kaiser Family Foundation, 2021 https://www.kff.org/policy-watch/global-covid-19-vaccine-access-snapshot-of-inequality/ (accessed Dec 3, 2021).

33 Grimsrud A, Wilkinson L. Acceleration of differentiated service delivery for HIV treatment in sub-Saharan Africa during COVID-19. *J Int AIDS Soc* 2021; **24**: e25704.

34 Ensheng Dong, Du H, Lauren Gardner. An interactive web-based dashboard to track COVID-19 in real time. *The Lancet Infectious Diseases* 2020; **20**: P533-534.

For peer review only

For peer review only - http://bmjopen.bmj.com/site/about/guidelines.xhtml

Figure 1. Total COVID cases and policy responses in Nepal from January 1, 2020, to September 16, 2020

The first recorded COVID case was reported on January 25th, 2020. The federal government implemented a nation-wide lockdown on March 24, 2020, including: stay-at-home requirements, closure of non-essential businesses, schools and all public transport, and restrictions on gathering and internal movements.[14] The National lockdown was lifted four months later on July 22, 2020, with major restrictions lifted, including stay-at-home requirements, workplace and public transport closures after which palika-specific response was allowed.
Source: COVID Data Repository by the Center for Systems Science and Engineering (CSSE) at Johns Hopkins University[34]

Figure 2. Primary care service utilization from Magh 2075 to Ashwin 2077 in Nepal (January 15, 2019 to September 16, 2020)

Months 1 to 20 are January 15, 2019 to September 16, 2020 (Magh 2075 to Bhadra 2077). For the purpose of our analysis, the national lockdown period (pre-period) includes months 15 to 18 (March 13 to July 16, 2020, Chaitra 2076 to Ashar 2077) and the post-period is Month 20 (August 17 to September 16, 2020, equivalent to Bhadra 2077). The orange lines represent average health care utilization in the control group: palikas that maintained COVID restrictions in the post period (e.g., stay-at-home requirements, business and public transport closures). The green lines represent health care utilization in the treatment group: palikas that lifted COVID restrictions in the post-period. Detailed definitions of health service indicators are in supplemental materials.

For peer review only

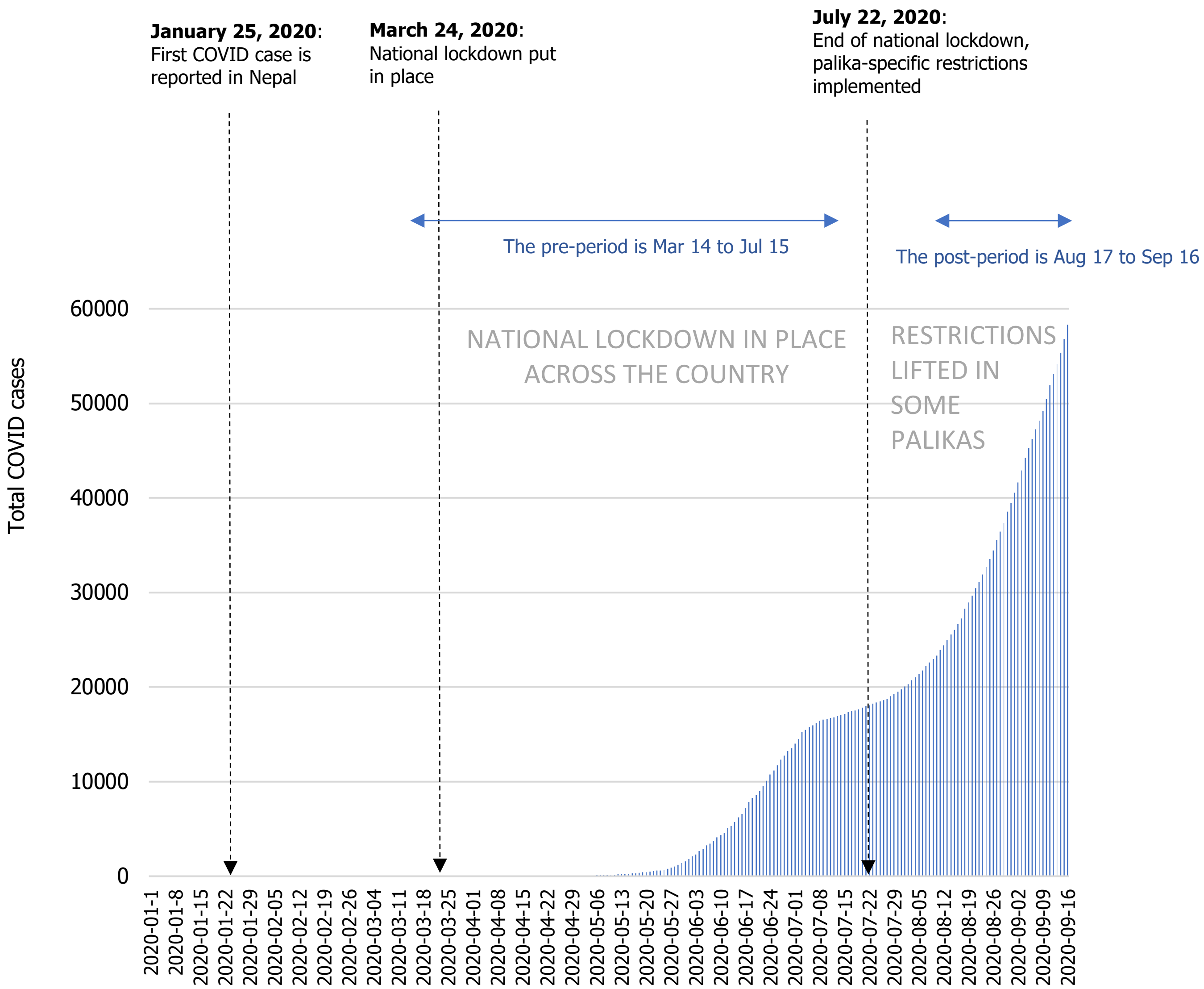
January 25, 2020: First COVID case is reported in Nepal
March 24, 2020: National lockdown put in place
July 22, 2020: End of national lockdown, palika-specific restrictions implemented
The pre-period is Mar 14 to Jul 15
The post-period is Aug 17 to Sep 16
NATIONAL LOCKDOWN IN PLACE ACROSS THE COUNTRY
RESTRICTIONS LIFTED IN SOME PALIKAS
Total COVID cases
60000
50000
40000
30000
20000
10000
0
2020-01-1
2020-01-8
2020-01-15
2020-01-22
2020-01-29
2020-02-05
2020-02-12
2020-02-19
2020-02-26
2020-03-04
2020-03-11
2020-03-18
2020-03-25
2020-04-01
2020-04-08
2020-04-15
2020-04-22
2020-04-29
2020-05-06
2020-05-13
2020-05-20
2020-05-27
2020-06-03
2020-06-10
2020-06-17
2020-06-24
2020-07-01
2020-07-08
2020-07-15
2020-07-22
2020-07-29
2020-08-05
2020-08-12
2020-08-19
2020-08-26
2020-09-02
2020-09-09
2020-09-16

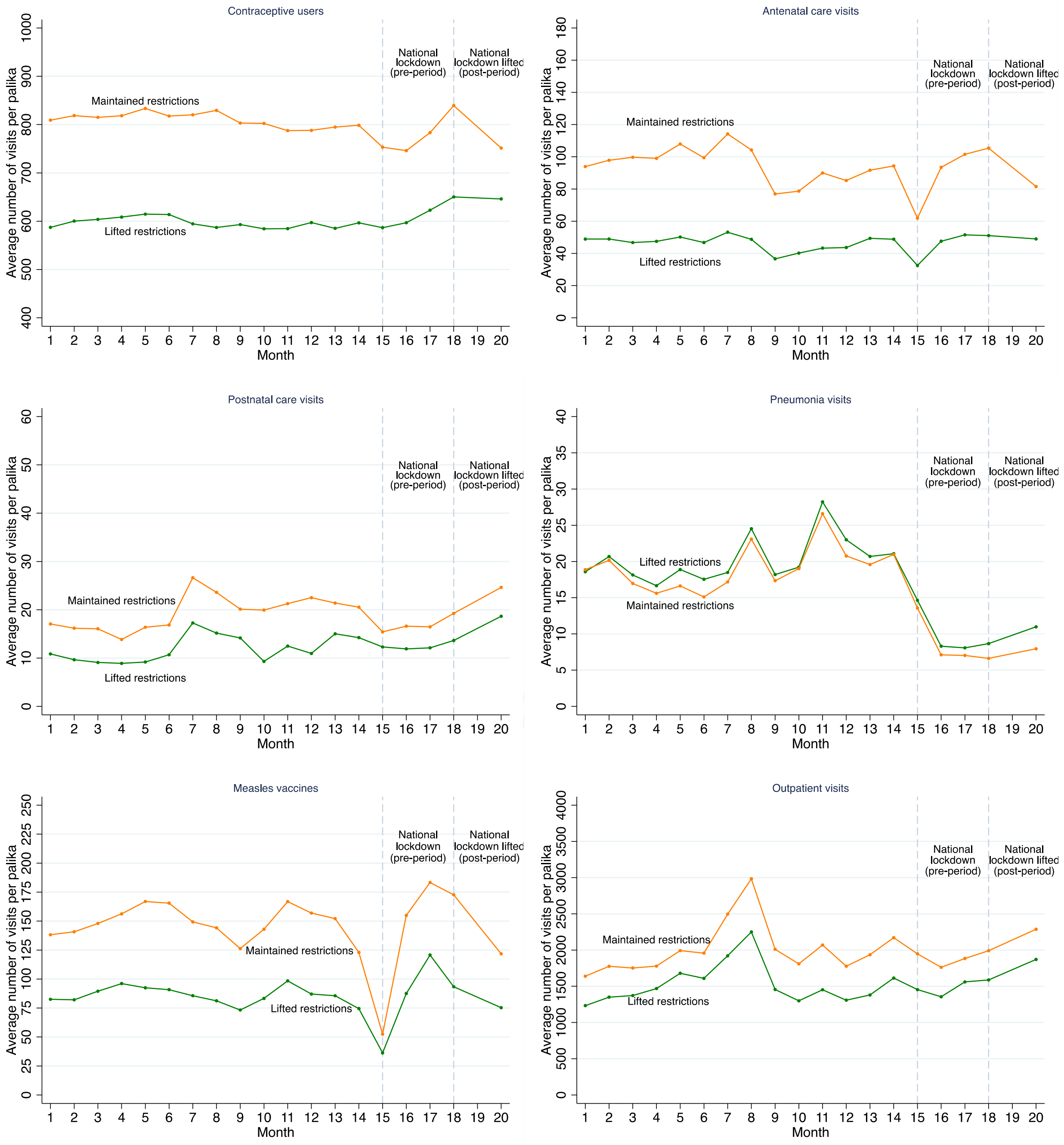
Contraceptive users
Antenatal care visits
Postnatal care visits
Pneumonia visits
Measles vaccines
Outpatient visits
Average number of visits per palika
Month
National lockdown (pre-period)
National lockdown lifted (post-period)
Maintained restrictions
Lifted restrictions

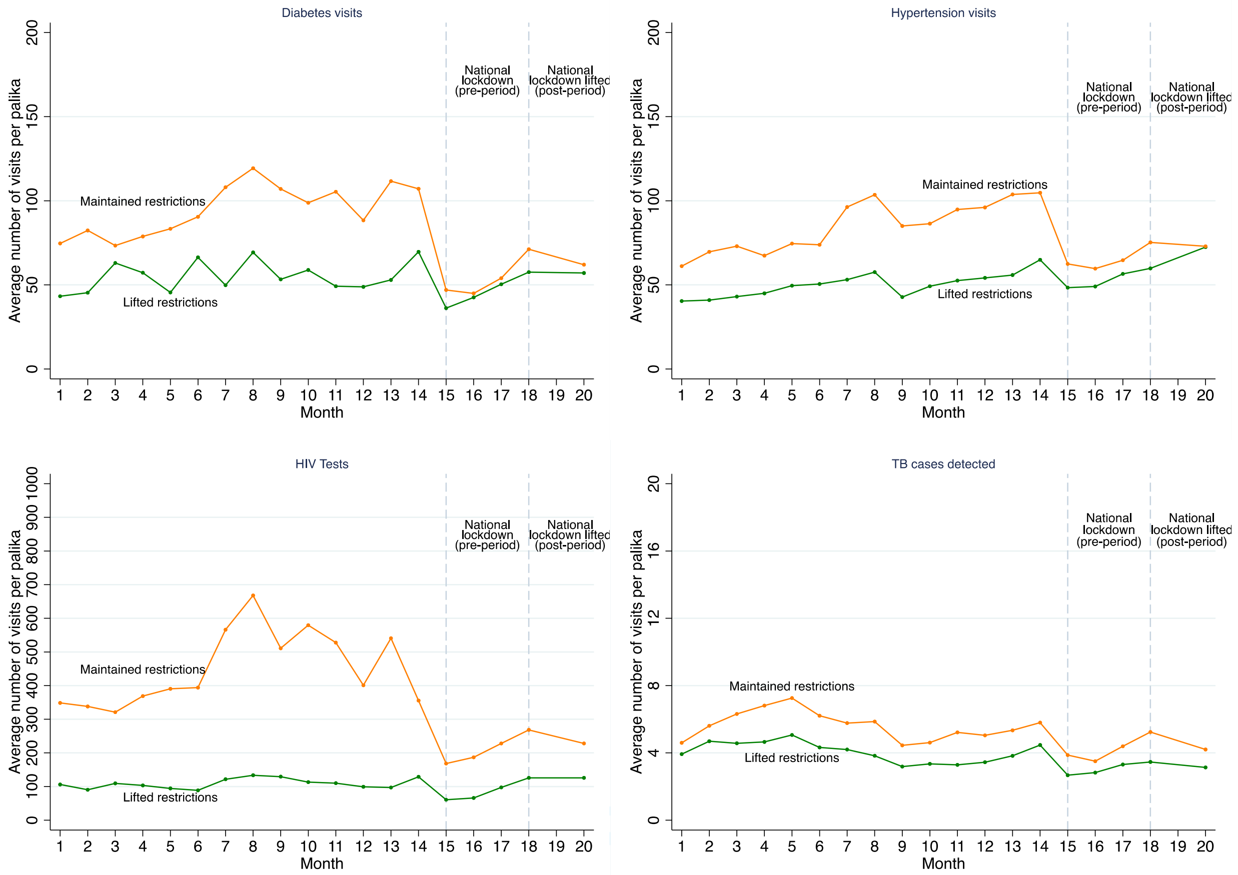

For peer review only

Title: The effect of lifting COVID-19 restrictions on utilization of primary care services in Nepal: a difference-in-differences analysis

Authors: Kapoor NR, Aryal A, Mehata S, Dulal M, Kruk ME, Bauhoff S, Arsenault C

Supplemental materials

Supplemental Table 1. Nepal DHIS2 Definitions for health service outcomes

Health service	Nepal DHIS2 Definition
**Outpatient visits**	Disaggregation by Sex & Caste/Ethnicity - Outpatient Cases
**Family planning**	Family Planning Program - Temporary FP Method - Depo-Current User +<br>Family Planning Program - Temporary FP Method - Depo-New Users < 20 Years +<br>Family Planning Program - Temporary FP Method - Depo-New Users > 20 Years +<br>Family Planning Program - Temporary FP Method - Pills- Current User +<br>Family Planning Program - Temporary FP Method - Pills- < 20 Years +<br>Family Planning Program - Temporary FP Method - Pills- > 20 Years +<br>Safe Motherhood Program-Safe Abortion Service-Post Abortion FP Methods Short Term-Medical +<br>Safe Motherhood Program-Safe Abortion Service-Post Abortion FP Methods Short term-Surgical +
**Antenatal care**	Safe Motherhood Program-Antenatal Checkup-First ANC visits (any time) < 20 years +<br>Safe Motherhood Program-Antenatal Checkup-First ANC visits (any time) > 20 years
**Postnatal care**	Safe Motherhood Program- Type of Delivery - 3 PNC visits as per protocol
**Pneumonia**	CBIMCI-(2-59Months)- Classification-ARI-Pneumonia +<br>CBIMCI-(2-59Months)-ORC Classification-ARI-Severe Pneumonia/Very Severe Disease
**Measles**	Immunization program - Children Immunized - Measles/Rubella - 9-11 Months + Immunization program - Children Immunized - Measles/Rubella - 12-23 Months
**HIV tests**	Virology-HIV tests conducted
**TB detection**	Disaggregation by Sex & Caste/Ethnicity- New TB Cases
**Diabetes visits**	Outpatient Morbidity-Nutritional & Metabolic Disorder-Diabetes Mellitus (DM) Cases
**Hypertension visits**	OPD-Morbidity-Cardiovascular & Respiratory Related Problems-Hypertension

Nepal DHIS2 definitions for primary care services.

For peer review only

Supplemental Table 2. Level of COVID-19 Restrictions in place from March 14, to September 16, 2020 in Nepal

	Mar 14, 2020 - April 12, 2020 (Chatira 2076)	April 13, 2020 - May 13, 2020 (Baisakh 2077)	May 14, 2020 - June 14, 2020 (Jestha 2077)	June 15, 2020 - July 15, 2020 (Ashar 2077)	July 16, 2020 - August 16, 2020 (Shrawan 2077)	August 17, 2020 - September 16, 2020 (Bhadra 2077)
Stay-at-home required (except essentials)	National[1]	National	National	National	District or palika-specific[3]	District or palika-specific
Business/workplace closures required	National[1]	National	National	National	District or palika-specific[3]	District or palika-specific
Public transport closures	National[1]	National	National	National	District or palika-specific[3]	District or palika-specific
Restricted gatherings to <10	National[1]	National	National	National	National	National
Border closure	National[1]	National	National	National	National	National
School closures	National1	National	National	National	National	National
Restrictions on internal movement	National[1]	National	National	National	National	National

[1]These policies were put in place on or around March 22nd, 2020.
[2]These policies were lifted on or around July 22nd, 2020.

Sources:
Rayamajhee B, Pojhrel A, Syangtan G, *et al.* How Well the Government of Nepal Is Responding to COVID-19? An Experience From a Resource-Limited Country to Confront Unprecedented Pandemic. *Front Public Health* 2021; published online Feb 17. https://doi.org/10.3389/fpubh.2021.597808.

Thomas Hale, Noam Angrist, Rafael Goldszmidt, *et al.* A global panel database of pandemic policies policies (Oxford COVID-19 Government Response Tracker). *Nature Human Behaviour* 2021. DOI:10.1038/s41562-021-01079-8.

The Situation of Corona Virus (COVID-19) in Nepal: Daily Reports. INSECOnline. http://inseconline.org/en/covid-19/.

Pradhan TR. Nepal goes under lockdown for a week starting 6am Tuesday. The Kathmandu Post. 2020; published online March 23. https://kathmandupost.com/national/2020/03/23/nepal-goes-under-lockdown-for-a-week-starting-6am-tuesday.

Pradhan TR. Government decides to lift the four-month-long coronavirus lockdown, but with conditions. The Kathmandu Post. 2020; published online July 21. https://kathmandupost.com/national/2020/07/21/government-decides-to-lift-the-four-month-long-coronavirus-lockdown-but-with-conditions.

For peer review only

Supplemental Table 3. Estimated effect of lifting COVID-19 restrictions on primary care service utilization in Nepal, estimates from difference-in-differences models that exclude March 14, 2020 – April 12, 2020 (Chaitra 2076)

	Restrictions lifted	95% CI	COVID-19 cases	95% CI	N	R^2	adj. R^2
**Contraceptive users**	56.40*	[11.63,101.17]	-0.01	[-0.03,0.01]	2968	0.01	0.01
**ANC Visits**	18.32***	[8.31,28.33]	0.00	[-0.01,0.01]	2900	0.03	0.03
**PNC Visits**	-1.06	[-4.89,2.78]	0.00	[-0.00,0.00]	2076	0.07	0.07
**Child pneumonia visits**	1.45*	[0.11,2.80]	-0.00**	[-0.00,-0.00]	2220	0.03	0.03
**Measles vaccine**	17.60*	[2.69,32.50]	-0.01**	[-0.01,-0.00]	1392	0.08	0.07
**Outpatient visits**	-84.61	[-223.74,54.53]	-0.10***	[-0.13,-0.06]	2968	0.10	0.10
**Diabetes visits**	3.61	[-8.74,15.95]	0.00	[-0.01,0.02]	1332	0.02	0.02
**Hypertension visits**	11.95	[-7.68,31.58]	0.00	[-0.01,0.01]	2756	0.02	0.01
**HIV tests**	33.24	[-11.71,78.20]	0.01	[-0.01,0.03]	944	0.04	0.03
**TB cases detected**	-0.02	[-0.83,0.78]	0.00	[-0.00,0.00]	920	0.05	0.05

95% confidence intervals in brackets
* $p < 0.05$, ** $p < 0.01$, *** $p < 0.001$
The coefficient for *Restrictions lifted* is the effect of lifting COVID-19 restrictions on health service utilization. Models also included fixed effects for month and palikas.

For peer review only - http://bmjopen.bmj.com/site/about/guidelines.xhtml

Supplemental Table 4. Joint F-tests for parallel trends assessment

	May * Restrictions lifted & June * Restrictions lifted Joint F-test (p-value)
Contraceptive Users	0.43
ANC Visits	0.16
PNC Visits	0.46
Child pneumonia visits	0.29
Measles vaccine	0.24
Outpatient visits	0.32
Diabetes visits	0.52
Hypertension visits	0.11
HIV tests	0.86
TB cases detected	0.16

P-values for joint F-test for *May * Restrictions lifted* and *June* Restrictions lifted* to test they are jointly not significantly different from zero.
May is May 14, 2020 to June 14, 2020 (Jestha 2077) and *June* is June 15, 2020 to July 15, 2020 (Ashar 2077). May and June are in the pre-period, during the national lockdown. The coefficients for *May*Restrictions lifted* and *June*Restrictions lifted* assess if trends are parallel in the pre-period, if the effect of lifting COVID-19 restrictions in palikas is significantly different from the baseline month, April 13 2020 to May 13 2020 (Baisakh 2076).

For peer review only

STROBE Statement—Checklist of items that should be included in reports of ***cross-sectional studies***

	Item No	Recommendation	Page No
**Title and abstract**	1	(*a*) Indicate the study's design with a commonly used term in the title or the abstract	1, 2
		(*b*) Provide in the abstract an informative and balanced summary of what was done and what was found	2
**Introduction**			
Background/rationale	2	Explain the scientific background and rationale for the investigation being reported	4
Objectives	3	State specific objectives, including any prespecified hypotheses	4,5
**Methods**			
Study design	4	Present key elements of study design early in the paper	6
Setting	5	Describe the setting, locations, and relevant dates, including periods of recruitment, exposure, follow-up, and data collection	6
Participants	6	(*a*) Give the eligibility criteria, and the sources and methods of selection of participants	6,7
Variables	7	Clearly define all outcomes, exposures, predictors, potential confounders, and effect modifiers. Give diagnostic criteria, if applicable	7
Data sources/ measurement	8*	For each variable of interest, give sources of data and details of methods of assessment (measurement). Describe comparability of assessment methods if there is more than one group	6,7
Bias	9	Describe any efforts to address potential sources of bias	7
Study size	10	Explain how the study size was arrived at	6,7
Quantitative variables	11	Explain how quantitative variables were handled in the analyses. If applicable, describe which groupings were chosen and why	6,7
Statistical methods	12	(*a*) Describe all statistical methods, including those used to control for confounding	7
		(*b*) Describe any methods used to examine subgroups and interactions	7
		(*c*) Explain how missing data were addressed	7
		(*d*) If applicable, describe analytical methods taking account of sampling strategy	7
		(*e*) Describe any sensitivity analyses	7
**Results**			
Participants	13*	(a) Report numbers of individuals at each stage of study—eg numbers potentially eligible, examined for eligibility, confirmed eligible, included in the study, completing follow-up, and analysed	9, 17
		(b) Give reasons for non-participation at each stage	9
		(c) Consider use of a flow diagram	NA
Descriptive data	14*	(a) Give characteristics of study participants (eg demographic, clinical, social) and information on exposures and potential confounders	9, 17, 18
		(b) Indicate number of participants with missing data for each variable of interest	9,17
Outcome data	15*	Report numbers of outcome events or summary measures	9,17
Main results	16	(*a*) Give unadjusted estimates and, if applicable, confounder-adjusted estimates and their precision (eg, 95% confidence interval). Make clear which confounders were adjusted for and why they were included	9, 17, 20

For peer review only - http://bmjopen.bmj.com/site/about/guidelines.xhtml

For peer review only

		(*b*) Report category boundaries when continuous variables were categorized	NA
		(*c*) If relevant, consider translating estimates of relative risk into absolute risk for a meaningful time period	9
Other analyses	17	Report other analyses done—eg analyses of subgroups and interactions, and sensitivity analyses	9
**Discussion**			
Key results	18	Summarise key results with reference to study objectives	10
Limitations	19	Discuss limitations of the study, taking into account sources of potential bias or imprecision. Discuss both direction and magnitude of any potential bias	11
Interpretation	20	Give a cautious overall interpretation of results considering objectives, limitations, multiplicity of analyses, results from similar studies, and other relevant evidence	10, 11
Generalisability	21	Discuss the generalisability (external validity) of the study results	11, 12
**Other information**			
Funding	22	Give the source of funding and the role of the funders for the present study and, if applicable, for the original study on which the present article is based	12

*Give information separately for exposed and unexposed groups.

Note: An Explanation and Elaboration article discusses each checklist item and gives methodological background and published examples of transparent reporting. The STROBE checklist is best used in conjunction with this article (freely available on the Web sites of PLoS Medicine at http://www.plosmedicine.org/, Annals of Internal Medicine at http://www.annals.org/, and Epidemiology at http://www.epidem.com/). Information on the STROBE Initiative is available at www.strobe-statement.org.

For peer review only

For peer review only - http://bmjopen.bmj.com/site/about/guidelines.xhtml

BMJ Open

The effect of lifting COVID-19 restrictions on utilization of primary care services in Nepal: a difference-in-differences analysis

Journal:	*BMJ Open*
Manuscript ID	bmjopen-2022-061849.R1
Article Type:	Original research
Date Submitted by the Author:	10-Aug-2022
Complete List of Authors:	Kapoor, Neena ; Harvard University T H Chan School of Public Health, Department of Global Health and Population<br>Aryal, Amit; Swiss Tropical and Public Health Institute<br>Mehata, Suresh; Government of Nepal Ministry of Health and Population<br>Dulal, Mahesh; Office of Member of Federal Parliament Nepal, Gagan Kumar Thapa<br>Kruk, Margaret; Harvard University T H Chan School of Public Health, Department of Global Health and Population<br>Bauhoff, Sebastian; Harvard University T H Chan School of Public Health, Department of Global Health and Population<br>Arsenault, C; Harvard University T H Chan School of Public Health, Department of Global Health and Population
**Primary Subject Heading**:	Health services research
Secondary Subject Heading:	Health policy, Public health, Global health
Keywords:	COVID-19, Health policy < HEALTH SERVICES ADMINISTRATION & MANAGEMENT, PRIMARY CARE

SCHOLARONE™
Manuscripts

I, the Submitting Author has the right to grant and does grant on behalf of all authors of the Work (as defined in the below author licence), an exclusive licence and/or a non-exclusive licence for contributions from authors who are: i) UK Crown employees; ii) where BMJ has agreed a CC-BY licence shall apply, and/or iii) in accordance with the terms applicable for US Federal Government officers or employees acting as part of their official duties; on a worldwide, perpetual, irrevocable, royalty-free basis to BMJ Publishing Group Ltd ("BMJ") its licensees and where the relevant Journal is co-owned by BMJ to the co-owners of the Journal, to publish the Work in this journal and any other BMJ products and to exploit all rights, as set out in our licence.

The Submitting Author accepts and understands that any supply made under these terms is made by BMJ to the Submitting Author unless you are acting as an employee on behalf of your employer or a postgraduate student of an affiliated institution which is paying any applicable article publishing charge ("APC") for Open Access articles. Where the Submitting Author wishes to make the Work available on an Open Access basis (and intends to pay the relevant APC), the terms of reuse of such Open Access shall be governed by a Creative Commons licence – details of these licences and which Creative Commons licence will apply to this Work are set out in our licence referred to above.

Other than as permitted in any relevant BMJ Author's Self Archiving Policies, I confirm this Work has not been accepted for publication elsewhere, is not being considered for publication elsewhere and does not duplicate material already published. I confirm all authors consent to publication of this Work and authorise the granting of this licence.

For peer review only

The effect of lifting COVID-19 restrictions on utilization of primary care services in Nepal: a difference-in-differences analysis

Authors: Kapoor NR[1*], Aryal A[2], Mehata S[3], Dulal M[4], Kruk ME[1], Bauhoff S[1], Arsenault C[1]

1. Department of Global Health and Population, Harvard T.H. Chan School of Public Health, Boston, USA
2. Swiss Tropical and Public Health Institute, University of Basel, Basel, Switzerland
3. Ministry of Health and Population, Government of Nepal, Kathmandu, Nepal
4. Office of Member of Federal Parliament Nepal, Gagan Kumar Thapa, Kathmandu, Nepal

***Corresponding Author:**
655 Huntington Ave, Boston MA, USA
nkapoor@hsph.harvard.edu

For peer review only

Abstract

Introduction An increasing number of studies have reported disruptions in health service utilization due to the COVID pandemic and its associated restrictions. However, little is known about the effect of lifting COVID restrictions on health service utilization. The objective of this study was to estimate the effect of lifting COVID restrictions on primary care service utilization in Nepal.

Methods Data on utilization of 10 primary care services were extracted from the Health Management Information System (HMIS) across all health facilities in Nepal. We used a difference-in-differences design and linear fixed effects regressions to estimate the effect of lifting COVID restrictions. The treatment group included palikas that had no restrictions in place from August 17 to September 16, 2020 (Bhadra 2077) and the control group included palikas that maintained restrictions during that period. The pre-period included the four months of national lockdown from March 24 to July 22, 2020 (Chaitra 2076-Ashar 2077). Models included month and palika fixed effects and controlled for COVID incidence.

Results We found that lifting COVID restrictions was associated with an average increase per palika of 57.5 contraceptive users (95% CI 14.6, 100.5), 15.6 antenatal care visits (95% CI 5.3, 25.9), and 1.6 child pneumonia visits (95% CI 0.2, 2.9). This corresponded to a 9.4% increase in contraceptive users, 34.2% increase in antenatal care visits and 15.6% increase in child pneumonia visits. Utilization of most other primary care services also increased after lifting restrictions, but coefficients were not statistically significant.

Conclusions Despite the ongoing pandemic, lifting restrictions can lead to an increase in some primary care services. Our results call for policy makers in low- and middle-income countries to carefully consider the tradeoffs of strict lockdowns during future COVID waves or future pandemics and prepare health systems for potential increases in service utilization as restrictions are lifted.

For peer review only

Strengths and limitations of this study

- We assessed the effect of lifting COVID-19 restrictions on primary care health service use in Nepal, which to our knowledge, has not been studied previously.
- We included data on 10 primary care services extracted from the Nepal Health Management Information System (HMIS).
- We used a differences-in-differences (DID) design to compare service use in palikas that lifted restrictions compared to those that maintained them.
- The DID design controls for time-fixed differences between palikas and temporal trends common to both groups. We controlled for new COVID cases at the district level but other time-varying confounders could affect the two groups differently.
- HMIS data provide real-time information on patterns in service use however, despite the data cleaning conducted, data quality issues and underreporting by some facilities could bias our results.

For peer review only

Background

In a time of crisis, high quality health systems have two tasks: respond to the crisis and maintain the provision and quality of essential health services.[1] Health systems in low-income countries, that may already be under-funded, under-resourced, and over-burdened, may be particularly vulnerable during the COVID pandemic. An increasing number of studies have reported disruptions in health service utilization since the start of the pandemic in low- and middle-income countries (LMICs).[2]–[10] The ongoing COVID pandemic has directly strained health care systems around the world that are struggling to meet the physical resource, human resource (numbers and skills), and service coordination demands of the pandemic. In addition, the pandemic had indirect effects on primary health care utilization, as restrictions and lockdowns implemented by governments to reduce the spread of COVID could also affect people's ability or willingness to visit healthcare facilities for routine primary care.

Nepal is a lower-middle income country of South Asia with a population of 28.6 million.[11] The country has shown significant gains in health and healthcare utilization over the past decade that are at risk of being reversed by the pandemic. As of December 2021, more than 800,000 people have been infected with COVID across Nepal and a reported 11,594 people have died from COVID.[12] Despite the existence of effective vaccines, only 32.8% of the Nepali population is currently fully vaccinated against COVID.[12]

Following the declaration of the pandemic on March 11, 2020 by the World Health Organization, health care utilization declined substantially in Nepal, ranging from a 65% decline in tuberculosis (TB) case detection to a 4% decline in contraceptive use.[3] Declines in health service use may stem from the declaration of the pandemic itself (perceived threat), the actual number of new COVID cases reported in a given period (leading to a fear of infection when visiting facilities or to overburdened health facilities treating COVID patients) or from the restrictions imposed (i.e., lockdowns) to curb the spread of COVID. The barriers imposed by COVID restrictions (e.g., stay-at home requirements or public transport closures) may play an important role in decreasing health care utilization. In Madesh Pradesh (formerly known as Province 2), Nepal, people reported that the national lockdown restricted accessibility to health facilities and deterred them from seeking care.[13] An increasing number of studies have described the effects of the pandemic and associated restrictions on health care utilization.[2]–[10] However, little is known about the effect of lifting COVID restrictions on health care utilization. Understanding these effects is crucial to plan for potential rebounds in demand and determining whether potentially weakened health systems can cope with surges in demand.

The Government of Nepal implemented a country-wide lockdown with strict restrictions on movement of people and closure of non-essential businesses on March 24, 2020.[14], [15] After almost four months of strict restrictions, the decision was made to lift most of these restrictions at the national level and end the four-month lockdown on July 22, 2020.[16] However, some of Nepal's 77 districts and 753 urban and rural municipality governments (*palikas, a local of government in Nepal's federal system)* maintained restrictions to contain the spread of COVID. This contrast in removal of restrictions gave rise to a natural experiment that allowed us to estimate the causal effect of lifting COVID restrictions on health care utilization.

For peer review only - http://bmjopen.bmj.com/site/about/guidelines.xhtml

In this study, we used a difference-in-differences design (DID) to estimate the effect of lifting COVID restrictions on primary care service utilization in Nepal. Understanding the effect of lifting COVID restrictions on primary healthcare is crucial to inform policy responses during future waves of COVID or future pandemics in low- and middle-income countries.

For peer review only

Methods

Data sources

We used data from the Nepal Health Management Information System (HMIS) obtained through the DHIS2 platform. The HMIS in Nepal includes information from both public and private facilities across all levels of the health system in Nepal.[17] A total of 7,605 health facilities are expected to report to the DHIS2 across 753 palikas.

Information on the types of COVID-19 restrictions in place was obtained from various sources including: INSECOnline, a human rights news portal in Nepal providing daily COVID updates, the Nepal COVID Crisis Management Coordination Center (CCMCC) government sites, District Administration Office (DAO) sites and additional online news sources (**supplementary material table 1**).[18]–[20]

We also included data on the total number of COVID cases at district level in Nepal (COVID case count was not available at the palika level). Monthly COVID cases in each of the 77 districts were obtained from the Nepal Health Emergency Operation Centre, Ministry of Health and Population.[21]

Measures

Primary care service utilization

We aimed to include 12 primary care services: contraceptive users, antenatal care (ANC) visits, postnatal care (PNC) visits, visits for children under five with pneumonia, visits for children under five with diarrhea, pentavalent vaccinations, measles vaccinations, visits for diabetes, visits for hypertension, number of human immunodeficiency virus (HIV) tests conducted, number of tuberculosis (TB) cases detected and total outpatient visits. Detailed definitions are in **supplementary material table 2.**

We obtained the monthly number of each of these services provided from January 15, 2019, to January 13, 2021 (Nepali calendar Magh 2077 to Poush 2077). These data were available at the palika level (currently 753 palikas in Nepal).

Because DHIS2 data are self-reported by health facilities, these data may contain errors. Our data cleaning procedures entailed identifying positive outliers (greater than 3.5 standard deviations from the mean trend) and setting any outliers as missing.[22] We did not assess negative outliers since decreases in utilization were expected during the lockdown period. For each health service, we also excluded palikas that were missing any data during the five-month study period (a complete case analysis).

COVID-19 restrictions

From March 24 to July 22, 2020, the Federal Government of Nepal imposed a strict nation-wide lockdown in response to the pandemic. This included stay-at-home requirements except for essential services, businesses, public transport and school closures, and restrictions on large

For peer review only

gatherings, international travel, and internal movement (see **supplementary material table 3**). On July 22, the major restrictions were lifted at the national level (including stay-at-home requirements, non-essential business, and public transport closures) but some districts and palikas maintained these restrictions. Following the lifting of the national lockdown, 248 palikas lifted the restrictions while 505 palikas maintained one or more of these restrictions.

For this analysis, the treatment group includes the palikas that lifted the restrictions, while the control group includes palikas that continued at least one or more restriction. The pre-intervention period includes the four months from March 14 to July 15 (which corresponds to the Nepali months of Chaitra 2076 to Ashar 2077) and the post-intervention period is August 17 to September 16, 2020 (the Nepali month of Bhadra 2077). July 16th through August 16th, 2020 (Shrawan 2077) was excluded from the analysis since lifting of the National lockdown occurred mid-month. Our analysis used Nepal calendar months as the unit of time.

The classification of palikas into treated and control groups was done using primarily INSEConline, an online news portal that provided daily updates on the COVID-19 situation in Nepal.[18] Four of the co-authors extracted information on the types of restrictions in place in each palika from the INSEConline news reports and verified and complemented the information with CCMCC government sites, DAO sites, and additional news sources (**supplementary material table 1**).[19], [20] Any disagreements were resolved through discussion. We used a 10-day threshold as a general rule of thumb. If restrictions were in place for less than 10 days during the month, the palika was considered as having lifted the restrictions and was included in the treatment group. If the restrictions covered more than 10 days, the palika remained in the control group (maintained restrictions). However, given imprecision in some of the policy reports, it was not always possible to apply this threshold with precision in some palikas.

Figure 1 shows the timeline of COVID restrictions and cases in Nepal from January 1, 2020, to September 16, 2020. The first COVID case was reported in Nepal on January 25, 2020. Notably, the end of the national lockdown on July 22, 2020 coincided with the beginning of the first real COVID wave (**figure 1**).

Statistical analysis

The analysis was conducted at the palika level, using Nepali calendar months as the unit of time. We used a DID design and fixed effects ordinary least square regression models. The following model was used and repeated for each of the health services analyzed:

$$S_{pt} = \alpha + \beta[lifted\ lockdown_{pt}] + \gamma_t + \delta_p + X_{dt} + \varepsilon_{pt}$$

Where S_{pt} is the number of health services (number of visits or users) provided in palika p in month t, γ_t and δ_p are vectors of month and palika fixed effects, respectively, and X_{dt} is the number of new COVID cases in district d and month t. The coefficient of interest is β, which represents the difference in service utilization among palikas that lifted restrictions compared to those that maintained restrictions. The palika fixed effects controls for time-invariant differences between palikas and avoids the need to control for time-fixed confounders. For example, the

For peer review only - http://bmjopen.bmj.com/site/about/guidelines.xhtml

palika fixed effects will control for unmeasured differences between palikas (urbanicity, population size, wealth) that can affect service utilization. The DID design also controls for all factors commonly affecting the outcomes in all palikas over time, through month fixed effects. COVID incidence would be associated with both the exposure (restrictions), and the outcome (health service utilization) and may vary between the treatment and control groups. Thus, we included monthly COVID cases in the regression models to control for potential confounding. Models also included clustered standard errors at the palika level.

A main assumption of DID models is that the outcome trend in the control group represents a good approximation of what the outcome trend would have been in the treatment group in the absence of the policy change (i.e., the counterfactual trend). Thus, to probe the assumption that the control palika trends were a good counterfactual for the treatment group (palikas that lifted restrictions), we implemented a series of tests. First, we conducted a pre-trend placebo test by comparing the difference in service utilization between the treated and control palikas in May and June 2020 (Jestha and Ashar 2077) compared to April 2020 (Baisakh 2077). We performed a joint F test of whether these coefficients were jointly zero (**supplementary materials**). Since all palikas were under the same lockdown in March to June 2020 (Chaitra 2076 to Ashar 2077), there should be no effect, providing evidence for parallel trends in the pre-period. Second, we assessed the parallel trend assumption graphically (**figure 2**). We also conducted a sensitivity analysis, excluding March 14 to April 12, 2020 (Chaitra 2076) from the analysis, since the national lockdown was put in place in the middle of this month, to see if the results differed. This research was approved by the Nepal Health Research Council (NHRC), reference number 650, and determined to be exempt from a full review by the Institutional Review Board (IRB) of the Harvard T.H. Chan School of Public Health.

Patient and public involvement

Patients will be involved in dissemination of this research. There was no patient or public involvement in the design, reporting, or interpretation of results.

For peer review only - http://bmjopen.bmj.com/site/about/guidelines.xhtml

Results

The average number of services provided during the national lockdown (pre-intervention period) and after the national lockdown was lifted (post-intervention period) for treated and control palikas are in **Table 1**. It also shows the number of palikas analyzed for each health service. Service utilization tended to be lower in the treatment group for a majority of services. The palikas in the treatment group, those that lifted restrictions, had less COVID cases and smaller populations on average compared to the palikas in the control group. These differences are accounted for by the palika fixed effects in the DID design.

Table 1: Number and characteristics of palikas by treatment group and period

	**Treatment group (lifted restrictions)**		**Control group (maintained restrictions)**	
	**Pre-period**	**Post-period**	**Pre-period**	**Post-period**
	Average per month (N)	Average per month (N)	Average per month (N)	Average per month (N)
**Contraceptive users**	614.09 (245)	646.10 (245)	780.52 (497)	751.41 (497)
**Antenatal care visits**	45.66 (235)	48.97 (235)	90.57 (490)	81.52 (490)
**Postnatal care visits**	12.49 (182)	18.65 (182)	16.93 (337)	24.63 (337)
**Child pneumonia visits**	9.92 (186)	10.98 (186)	8.59 (369)	7.95 (369)
**Measles Vaccine**	84.47 (102)	75.28 (102)	140.83 (246)	121.74 (246)
**Outpatient visits**	1489.23 (243)	1870.75 (243)	1896.25 (499)	2286.77 (499)
**Diabetes visits**	46.66 (100)	57.04 (100)	54.27 (233)	62.00 (233)
**Hypertension visits**	53.43 (220)	72.44 (220)	65.50 (469)	72.93 (469)
**HIV tests**	87.55 (68)	125.88 (68)	212.92 (168)	228.17 (168)
**TB cases detected**	3.07 (52)	3.13 (52)	4.25 (178)	4.20 (178)
**Average number of new COVID cases over the study period per palika**	287		1,004	
**Average palika population size**	23,649		46,007	

The average number of health visits per palika and month. The treatment group includes the palikas that lifted lockdowns in August 17 to September 16, 2020 (Bhadra 2077). The control group includes those that maintained lockdowns during that month. The (N) is the number of palikas reporting each month in the period for that service. National lockdown period (pre-period) includes March 13 to July 16, 2020 (Chaitra 2076 to Ashar 2077). The post-period includes August 17 to September 16, 2020 (Bhadra 2077). The average number of new COVID cases in each period is COVID cases at the district level since these data were not available at the palika level. The palika population size data was obtained from the Preliminary Data of National Population and Housing Census 2021.[23]

Figure 2 shows the trend in primary care service utilization from January 2019 to September 2020 (equivalent to Magh 2075 to Bhadra 2077) and reveals parallel trends before and during the national lockdown period (our pre-period) for all services included. A sharp decrease in utilization is observed in both groups of palikas at the start of the pandemic when the national lockdown was put in place (months 14 to 15 in **figure 2**). For most services, this decline was

For peer review only - http://bmjopen.bmj.com/site/about/guidelines.xhtml

followed by a gradual resumption in the pre-period. Given these similar trends in both groups, the control palikas appear to provide appropriate counterfactual trends in the post-period. The joint F-test (**supplementary material table 4**) also did not reject the null hypothesis that the outcomes evolved differently in treated vs. control palikas in the pre-period for the 10 services included. The parallel trend assumption was violated for two health services: visits for children under five with diarrhea and pentavalent vaccinations, which were excluded from the analysis.

Table 2. Estimated effect of lifting COVID restrictions on primary care service utilization in Nepal, estimates from difference-in-differences models

	Restrictions lifted	95% CI	COVID cases	95% CI	*N*	R^2	adj. R^2
**Contraceptive users**	57.51**	[14.55,100.48]	-0.01	[-0.02,0.01]	3710	0.01	0.01
**ANC visits**	15.60**	[5.34,25.86]	0.01	[-0.01,0.02]	3625	0.08	0.07
**PNC visits**	-1.50	[-4.94,1.94]	0.00	[-0.00,0.00]	2595	0.07	0.07
**Child pneumonia visits**	1.55*	[0.24,2.86]	-0.00**	[-0.00,-0.00]	2775	0.19	0.19
**Measles vaccine**	7.35	[-6.49,21.19]	0.00	[-0.01,0.00]	1740	0.20	0.20
**Outpatient visits**	-56.81	[-193.77,80.16]	-0.10***	[-0.13,-0.06]	3710	0.09	0.09
**Diabetes visits**	5.01	[-8.12,18.14]	0.00	[-0.01,0.02]	1665	0.03	0.02
**Hypertension visits**	12.70	[-6.74,32.14]	0.00	[-0.01,0.01]	3445	0.02	0.02
**HIV tests**	34.83	[-12.57,82.22]	0.02	[-0.02,0.05]	1180	0.04	0.04
**TB cases detected**	0.06	[-0.73,0.85]	0.00	[-0.00,0.00]	1150	0.04	0.04

95% confidence intervals in brackets
* $p < 0.05$, ** $p < 0.01$, *** $p < 0.001$
The coefficient for *Restrictions lifted* is the effect of lifting COVID restrictions on health service utilization. Models also included fixed effects for month and palika.

Estimates from DID regressions are reported in **Table 2** for the 10 health services. The coefficient for *restrictions lifted* is the DID estimate and can be interpreted as the difference in adjusted service utilization between the treatment (lifted restrictions) and control (maintained restrictions) palikas in the post-period.

Lifting COVID restrictions led to a positive increase in all services except total outpatient visits and postnatal care visits. These effects were statistically significant for three services. Lifting restrictions led to an average increase per palika of 57.5 contraceptive users (95% Confidence Interval (CI) 14.6-100.5), 15.6 antenatal care visits (95% CI 5.3-25.9), and 1.6 child pneumonia visits (95% CI 0.2-2.9). Compared to the pre-COVID average utilization, this represented a 9.4% increase in contraceptive use, 34.2% increase in antenatal care visits and a 15.6% increase in child pneumonia visits.

Similarly, although not statistically significant, lifting restrictions led to 7.4 more children vaccinated against measles (95% CI -6.5-21.2), 5.0 more diabetes visits (95% CI -8.1-18.1), 12.7 more hypertension visits (95% CI -6.7-32.1), 34.8 additional HIV tests (95% CI -12.6-82.2) and 0.1 additional TB cases detected (95% -0.7-0.9) on average per palika. These increases were

For peer review only - http://bmjopen.bmj.com/site/about/guidelines.xhtml

equivalent to increases of 8.7% for measles vaccinations, 10.7% for diabetes visits, 23.8% for hypertension visits, 39.8% for HIV tests, and 2.0% for TB case detection in palikas that lifted restrictions compared to those that maintained it. In contrast, the coefficient for PNC and total outpatient visits were negative but these were not statistically significant. They were equivalent to declines of 12.0% fewer PNC visits and 3.8% fewer outpatient visits in palikas that lifted restrictions compared to those that didn't. Results from the sensitivity analysis that excluded March 14 to April 12, 2020 (Chaitra 2076) were largely consistent with the main model, for the exception of measles vaccinations which had a statistically significant increase in palikas that lifted restrictions (see **supplementary material table 5**).

For peer review only

Discussion

In this analysis, we used HMIS data and a differences-in-differences design to estimate the effect of lifting COVID restrictions on primary care health service utilization in Nepal. We found that lifting restrictions increased contraceptive use, antenatal care, and sick child visits by 9.4% to 34.2% on average across palikas. Utilization of most other primary care services also increased by 2.0% to 39.8% but were not statistically significant. These results provide evidence that COVID restrictions are linked to primary care service utilization in Nepal and that lifting these restrictions can lead to an increase in service uptake. To our knowledge, this is the first paper to estimate the effect of lifting COVID restrictions on health service utilization using a quasi-experimental method.

There are many mechanisms through which COVID-related restrictions (stay-at-home requirements, business/workplace closures and public transport closures) might affect primary healthcare utilization. People in Nepal have reported that public transport closures during the national lockdown prevented them from reaching healthcare facilities.[13] In addition, stay-at-home requirements meant that people were only permitted to leave their home for essential services. Though essential services included healthcare, stay-at-home requirements were firmly enforced by law enforcement officials, and individuals were arrested and jailed or fined if they defied them.[14], [24] Knowing this risk, people may have been deterred from seeking healthcare, despite being allowed. It is also possible that many people did not know that visiting health facilities was allowed during the lockdown. In addition, the strict lockdown may have increased anxieties around COVID which deterred people from seeking care.[13], [25] Once COVID restrictions were lifted, these barriers would be subdued, and an increase in utilization would be expected.

Lockdown policies, primarily stay-at-home requirements and business or workplace closures, can also have detrimental economic effects, potentially pushing low-income individuals and families further into poverty.[14], [26] Most of the Nepali population works in the informal sector, including a large number in the tourism industry, which was severely impacted by the pandemic. Although the government developed economic support packages, informal sector workers or other marginalized groups were often missed due to difficulties in implementation.[14] Essential health services are supposed to be free of charge in public facilities in Nepal.[27] However, Nepalis often incur costs at point of service, with more than half of health expenditures being made out-of-pocket.[27], [28] In addition, due to stockouts and staff shortages in public facilities, among other reasons, between 20% and 61% of people choose private sector facilities for primary care in Nepal, depending on the type of service.[27], [29], [30] Lifting COVID restrictions could increase households' ability to generate income, especially for those working in the informal sector, and may have allowed them to pay for health care costs and thus seek health services again.

In a prior study analyzing these same health services in Nepal, we found that following the declaration of the pandemic on March 11, 2020, primary health care utilization declined substantially.[3] Lifting COVID restrictions had statistically significant effects only for reproductive, maternal and child health services (RMNCH): contraceptives, antenatal care, and

For peer review only - http://bmjopen.bmj.com/site/about/guidelines.xhtml

child pneumonia visits. The government of Nepal has focused on improving and maintaining sexual, reproductive, maternal, newborn, child and adolescent health (SRMNCAH), both historically and during the pandemic, which could explain why these services significantly increased after restrictions were lifted, in comparison to other primary care services.[31] In Nepal, policymakers suspended Measles-Rubella vaccinations campaigns, but aware of the risk of outbreaks, decided to continue the campaign after only one month. Furthermore, SRMNCAH services were monitored and mapped by Nepal's government during the COVID pandemic. The Country Preparedness and Response Plan focused on SRMNCAH services and interim guidelines for SRMNCAH were endorsed.[31] We found no impact of lifting COVID restrictions on PNC visits. This could be due to a large program for postnatal care home visits and outreach which was launched shortly before the pandemic and continued during the lockdown in some districts. This could explain why there were no differences between palikas that lifted restrictions and those that maintained them.

Other studies have found declines in health service utilization of various magnitude and duration following the declaration of the pandemic and the implementation of restrictions in many countries.[2]–[10] Studies from Nepal found declines in primary care and hospital-based care during the pandemic including fewer deliveries and a potential increase in neonatal mortality and institutional stillbirths.[3], [8] In contrast, we assessed the effect of lifting restrictions, and the resulting increases in service use. Our DID design estimates the causal effect of lifting COVID restrictions. DID compares trends between the treatment and comparison groups and compares each unit to itself, estimating an average of the counterfactual DID contrasts.

Nonetheless, our study has limitations. The DID design controls for time fixed differences, such as population size, between palikas and for secular trends affecting all groups. However, it is possible that remaining time-varying confounders affected the two groups differently. For example, although we adjusted for COVID caseloads at the district level, it is possible that palika-specific outbreaks influenced the decision to maintain restrictions. Another potential concern relates to measurement error for both the policies and the health service utilization outcomes. The exposure variable may have also been misclassified due to missing information on the restrictions in place in palikas. Although multiple sources were reviewed to collect and confirm the implementation of these restrictions, these sources sometimes lacked precision. Any misclassification due to missing information would likely lead the palika to be included in the treatment group (lifted restrictions) when they actually maintained restrictions. This would bias the results towards the null. DHIS2 data may also contain errors, and reporting quality may have been affected by the pandemic. However, positive outliers were removed and only facilities that reported each indicator completely every month over the study period were included. It is unclear whether DHIS2 data quality issues would affect our analysis since misreporting should be similar in both the treatment and control groups. The estimates for TB case detection and measles vaccination must also be interpreted with caution as an important number of observations were excluded by the complete case analysis (see **supplementary material table 6**). In addition, we were limited by the type of data available in the Nepal HMIS. For example, we did not have access to other primary care services, like mental health visits, that might also be impacted by COVID restrictions. Finally, the outcome data was only available monthly, and the beginning and end of restrictions did not always match DHIS2 data precisely. Thus, policy dates and outcomes were not perfectly matched. In our dataset, the pre-period begins 10 days before

For peer review only - http://bmjopen.bmj.com/site/about/guidelines.xhtml

the national lockdown. However, sensitivity analyses that excludes the first month of the lockdown shows similar results (**supplementary material table 5**).

Our results have important implications for policy. We found that despite the ongoing COVID pandemic, lifting restrictions can lead to an increase in RMNCH service utilization. Universal utilization of these services is crucial to improve health outcomes. Antenatal care visits are essential to identify conditions that might threaten the mother or newborn's health.[32] It is estimated that a 10% decrease in coverage of pregnancy related and newborn health care during COVID-19 could result in an additional 28,000 maternal deaths and 168,000 neonatal deaths globally.[33] In addition, reduced contraceptive use could results in more unintended pregnancies which can also place both the pregnant person and child at risk.[34] Delayed care for respiratory illnesses during COVID restrictions could increase the incidence of pneumonia. Pneumonia is one of the leading causes of death for children under five, and missed care could further exacerbate this burden.[35] Nonetheless, it is important to note that an increase in child pneumonia visits after restrictions were lifted could be linked to an increase in health needs from further spread of respiratory illnesses rather than a pent up demand.

Our study contributes to the literature on the indirect effects of COVID-19 restrictions on health systems. Although effective vaccines are now available, few people in LMICs are fully immunized against COVID due to widespread inequities in access to vaccines.[36] Future waves of COVID infections and emerging variants are likely to push governments to consider re-implementing temporary restrictions and lockdowns. At the start of the pandemic, many countries took a one-size-fits-all approach with COVID containment policies, as there was understandably much uncertainty surrounding COVID and its effects. As we gain insight into the indirect effects of these restrictions, it is important that policy-makers tailor these policies to their own demographic, disease, and sociocultural contexts, and prepare health systems to respond accordingly.[26] Policy makers should consider strategies to promote and maintain all types of primary care services during future waves of COVID and future pandemics. Such strategies may include better risk communication on the importance of essential health care and alternative service delivery modes such as telemedicine or differentiated service delivery strategies.[37] Health facilities should also be prepared to face potential increases in demand for health care when restrictions are eased.

Contribution statement

NRK, CA, SB and MEK designed the study. NRK, CA, SM, AA and MD compiled and verified the data. NRK and CA led the data analysis. NRK wrote the first draft and all other co-authors, contributed to the interpretation of findings and read, improved and approved the final manuscript.

Funding

This work is part of the Grand Challenges ICODA pilot initiative, delivered by Health Data Research UK and funded by the Bill & Melinda Gates Foundation and the Minderoo Foundation (reference number 2021.0091). We also acknowledge funding from the Bill and Melinda Gates Foundation (grant INV-005254).

Competing interest

For peer review only - http://bmjopen.bmj.com/site/about/guidelines.xhtml

There are no competing interests for any authors.

Ethics approval

This study does not involve human participants.

Data availability

Data may be obtained from a third party and are not publicly available.

For peer review only

For peer review only - http://bmjopen.bmj.com/site/about/guidelines.xhtml

References:

[1] Kruk, M. E. *et al.*, “High-quality health systems in the Sustainable Development Goals era: time for a revolution,” *Lancet Glob Health*, vol. 6, no. 11, pp. e1196–e1252, Nov. 2018, doi: 10.1016/S2214-109X(18)30386-3.

[2] Moynihan, R *et al.*, “Impact of COVID-19 pandemic on utilisation of healthcare services: a systematic review,” *BMJ Open*, vol. 11, no. 3, Mar. 2021, doi: 10.1136/bmjopen-2020-045343.

[3] C. Arsenault *et al.*, “COVID-19 and resilience of healthcare systems in 10 countries,” *Nat Med*, vol. 28, pp. 1314–1324, Mar. 2022, doi: 10.1038/s41591-022-01750-1.

[4] Hategeka, C *et al.*, “Impact of the COVID-19 pandemic and response on the utilisation of health services in public facilities during the first wave in Kinshasa, the Democratic Republic of the Congo,” *BMJ Glob Health*, vol. 6, no. 7, Jul. 2021, [Online]. Available: https://www.ncbi.nlm.nih.gov/pubmed/34315776

[5] Doubova, SV, Leslie, HH, Kruk, ME, Perez-Cuevas, R, and Arsenault, C, “Disruption in essential health services in Mexico during COVID-19: an interrupted time series analysis of health information system data,” *BMJ Glob Health*, vol. 6, no. 9, doi: 10.1136/bmjgh-2021-006204.

[6] Burt, JF *et al.*, “Indirect effects of COVID-19 on maternal, neonatal, child, sexual and reproductive health services in Kampala, Uganda,” *BMJ Glob Health*, vol. 6, no. 8, Aug. 2021, doi: 10.1136/bmjgh-2021-006102.

[7] Z. Aranda *et al.*, “Disruptions in maternal health service use during the COVID-19 pandemic in 2020: experiences from 37 health facilities in low-income and middle-income countries,” *BMJ Global Health*, vol. 7, no. 1, p. e007247, Jan. 2022, doi: 10.1136/bmjgh-2021-007247.

[8] A. KC *et al.*, “Effect of the COVID-19 pandemic response on intrapartum care, stillbirth, and neonatal mortality outcomes in Nepal: a prospective observational study,” *The Lancet Global Health*, vol. 8, no. 10, pp. e1273–e1281, Oct. 2020, doi: 10.1016/S2214-109X(20)30345-4.

[9] G. Shapira *et al.*, “Disruptions in maternal and child health service utilization during COVID-19: analysis from eight sub-Saharan African countries,” *Health Policy Plan*, vol. 36, no. 7, pp. 1140–1151, Aug. 2021, doi: 10.1093/heapol/czab064.

[10] T. Ahmed *et al.*, “Indirect Effects on Maternal and Child Mortality from the COVID-19 Pandemic: Evidence from Disruptions in Healthcare Utilization in 18 Low- and Middle-Income Countries.” Rochester, NY, Sep. 09, 2021. doi: 10.2139/ssrn.3916767.

[11] “The World Bank Data,” 2019. [Online]. Available: https://data.worldbank.org/country/nepal

[12] “Our World in Data,” 2021. [Online]. Available: https://ourworldindata.org/coronavirus/

[13] Singh, DR *et al.*, “Impact of COVID-19 on health services utilization in Province-2 of Nepal: a qualitative study among community members and stakeholders,” *BMC Health Serv Res*, vol. 21, no. 1, p. 174, Feb. 2021, doi: 10.1186/s12913-021-06176-y.

[14] B. Rayamajhee *et al.*, “How Well the Government of Nepal Is Responding to COVID-19? An Experience From a Resource-Limited Country to Confront Unprecedented Pandemic,” *Front. Public Health*, Feb. 2021, [Online]. Available: https://doi.org/10.3389/fpubh.2021.597808

[15] T. R. Pradhan, “Nepal goes under lockdown for a week starting 6am Tuesday,” *The Kathmandu Post*, Mar. 23, 2020. [Online]. Available:

For peer review only

For peer review only - http://bmjopen.bmj.com/site/about/guidelines.xhtml

https://kathmandupost.com/national/2020/03/23/nepal-goes-under-lockdown-for-a-week-starting-6am-tuesday

[16] T. R. Pradhan, "Government decides to lift the four-month-long coronavirus lockdown, but with conditions," *The Kathmandu Post*, Jul. 21, 2020. [Online]. Available: https://kathmandupost.com/national/2020/07/21/government-decides-to-lift-the-four-month-long-coronavirus-lockdown-but-with-conditions

[17] "Annual report 2076/77 (2019/20)," Department of Health Services, Ministry of Health and Population, Government of Nepal, Jul. 2021. [Online]. Available: https://dohs.gov.np/annual-report-2076-77-2019-20/

[18] "The Situation of Corona Virus (COVID-19) in Nepal: Daily Reports," *INSECOnline*. [Online]. Available: http://inseconline.org/en/covid-19/

[19] "Government of Nepal, Office of the Prime Minister and Council of Ministers, Covid-19 Crisis Management Coordination Center (CCMCC)," *CCMCC*. https://ccmc.gov.np/

[20] "Government of Nepal, Ministry of Home Affairs," *Offices*. https://www.moha.gov.np/pro_admin/api/offices (accessed Aug. 03, 2022).

[21] "CoVid-19 Dasboard," Government of Nepal, Ministry of Health and Population. Accessed: Feb. 09, 2021. [Online]. Available: https://covid19.mohp.gov.np/

[22] "Module 2: Desk review of data quality," World Health Organization, 2, 2017.

[23] "Preliminary Data of National Population and Housing Census 2021," Government of Nepal, National Planning Commission Central Bureau of Statistics, Jan. 2022. [Online]. Available: https://opendatanepal.com/dataset/preliminary-data-of-national-population-and-housing-census-2021

[24] "Nepal Police crack down on 749 Kathmandu residents for violating Covid-19 lockdown," *India Today*, Mar. 30, 2020. [Online]. Available: https://www.indiatoday.in/world/story/nepal-police-crackdown-on-749-kathmandu-residents-for-violating-covid-19-lockdown-1661343-2020-03-30

[25] U. N. Yadav *et al.*, "Perceived fear of COVID-19 and its associated factors among Nepalese older adults in eastern Nepal: A cross-sectional study," *PLOS ONE*, vol. 16, no. 7, p. e0254825, Jul. 2021, doi: 10.1371/journal.pone.0254825.

[26] R. Cash and V. Patel, "Has COVID-19 subverted global health?," *Lancet*, vol. 395, no. 10238, pp. 1687–1688, 2020, doi: 10.1016/S0140-6736(20)31089-8.

[27] "Free Health Care in Nepal: Findings of a Rapid Assessment," GFA Consulting Group. [Online]. Available: https://files.givewell.org/files/DWDA%202009/Nyaya/en-free-health-care-nepal.pdf

[28] S. P. Kalaunee, "Is free health care possible in Nepal?," *Nepali Times*, Dec. 12, 2019. Accessed: Dec. 03, 2021. [Online]. Available: https://www.nepalitimes.com/opinion/is-free-health-care-possible-in-nepal/

[29] C. Arsenault *et al.*, "Hospital-provision of essential primary care in 56 countries: determinants and quality," *Bull World Health Organ*, vol. 98, no. 11, pp. 735-746D, Nov. 2020, doi: 10.2471/BLT.19.245563.

[30] S. Acharya, S. Ghimire, E. M. Jeffers, and N. Shrestha, "Health Care Utilization and Health Care Expenditure of Nepali Older Adults," *Front Public Health*, vol. 7, p. 24, Feb. 2019, doi: 10.3389/fpubh.2019.00024.

[31] "Responding to COVID-19: Health sector preparedness, response and lessons learnt," Government of Nepal, Ministry of Health and Population, Kathmandu, Nepal, 2021.

For peer review only - http://bmjopen.bmj.com/site/about/guidelines.xhtml

[32] D. Banta, "What is the efficacy/effectiveness of antenatal care and the financial and organizational implications?," WHO Regional Office for Europe, Copenhagen, Dec. 2003. [Online]. Available: https://www.euro.who.int/__data/assets/pdf_file/0007/74662/E82996.pdf
[33] Riley, Sully, Ahmed, and Biddlecom, "Estimates of the Potential Impact of the COVID-19 Pandemic on Sexual and Reproductive Health In Low- and Middle-Income Countries," *International Perspectives on Sexual and Reproductive Health*, vol. 46, p. 73, 2020, doi: 10.1363/46e9020.
[34] G. Weigel, A. Salganicoff, and U. Ranji, "Potential Impacts of Delaying 'Non-Essential' Reproductive Health Care," Kaiser Family Foundation, Jun. 2020.
[35] Bernadeta Dadonaite, *Pneumonia — no child should die from a disease we can prevent*, 2019. https://ourworldindata.org/child-deaths-from-pneumonia
[36] A. Rouw, A. Wexler, J. Kates, and J. Michaud, "Global COVID-19 Vaccine Access: A Snapshot of Inequality," Kaiser Family Foundation, Mar. 2021. Accessed: Dec. 03, 2021. [Online]. Available: https://www.kff.org/policy-watch/global-covid-19-vaccine-access-snapshot-of-inequality/
[37] A. Grimsrud and L. Wilkinson, "Acceleration of differentiated service delivery for HIV treatment in sub-Saharan Africa during COVID-19," *J Int AIDS Soc*, vol. 24, no. 6, p. e25704, Jun. 2021, doi: 10.1002/jia2.25704.
[38] E. Dong, H. Du, and L. Gardner, "An interactive web-based dashboard to track COVID-19 in real time," *The Lancet Infectious Diseases*, vol. 20, no. 5, pp. P533-534, May 2020, doi: 10.1016/S1473-3099(20)30120-1.

For peer review only

For peer review only - http://bmjopen.bmj.com/site/about/guidelines.xhtml

Figure 1. Total COVID cases and policy responses in Nepal from January 1, 2020, to September 16, 2020

The first recorded COVID case was reported on January 25th, 2020. The federal government implemented a nation-wide lockdown on March 24, 2020, including: stay-at-home requirements, closure of non-essential businesses, schools and all public transport, and restrictions on gathering and internal movements.[14] The National lockdown was lifted four months later on July 22, 2020, with major restrictions lifted, including stay-at-home requirements, workplace and public transport closures after which palika-specific response was allowed.
Source: COVID Data Repository by the Center for Systems Science and Engineering (CSSE) at Johns Hopkins University [38]

Figure 2. Primary care service utilization from Magh 2075 to Ashwin 2077 in Nepal (January 15, 2019 to September 16, 2020)

Months 1 to 20 are January 15, 2019 to September 16, 2020 (Magh 2075 to Bhadra 2077). For the purpose of our analysis, the national lockdown period (pre-period) includes months 15 to 18 (March 13 to July 16, 2020, Chaitra 2076 to Ashar 2077) and the post-period is Month 20 (August 17 to September 16, 2020, equivalent to Bhadra 2077). The orange lines represent average health care utilization in the control group: palikas that maintained COVID restrictions in the post period (e.g., stay-at-home requirements, business and public transport closures). The green lines represent health care utilization in the treatment group: palikas that lifted COVID restrictions in the post-period. Detailed definitions of health service indicators are in supplemental materials.

For peer review only

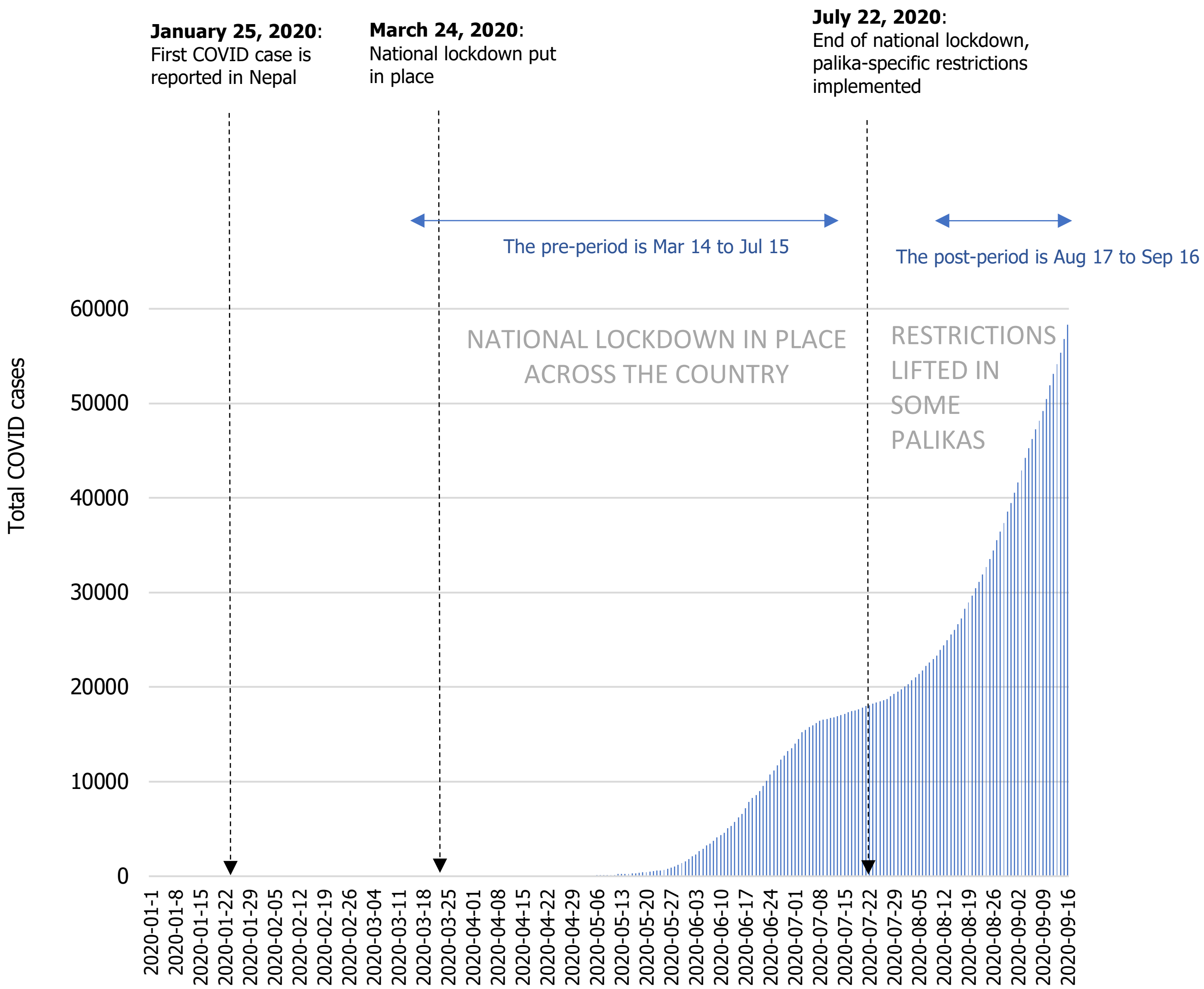

January 25, 2020: First COVID case is reported in Nepal
March 24, 2020: National lockdown put in place
July 22, 2020: End of national lockdown, palika-specific restrictions implemented
The pre-period is Mar 14 to Jul 15
The post-period is Aug 17 to Sep 16
NATIONAL LOCKDOWN IN PLACE ACROSS THE COUNTRY
RESTRICTIONS LIFTED IN SOME PALIKAS
Total COVID cases
60000
50000
40000
30000
20000
10000
0
2020-01-1
2020-09-16

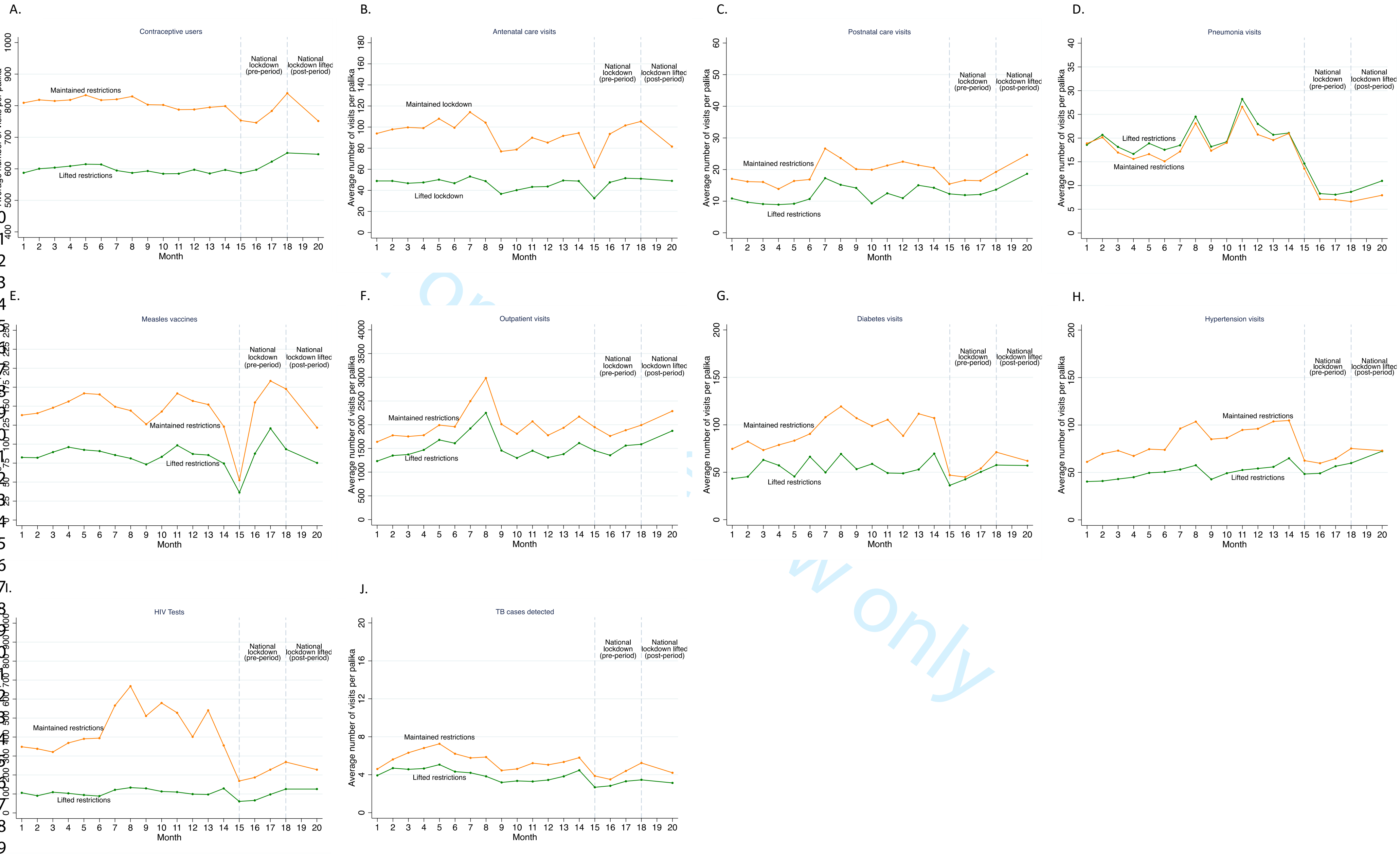
A.
Contraceptive users
Maintained restrictions
Lifted restrictions
National lockdown (pre-period)
National lockdown lifted (post-period)
Average number of visits per palika
Month
B.
Antenatal care visits
Maintained lockdown
Lifted lockdown
C.
Postnatal care visits
Maintained restrictions
Lifted restrictions
D.
Pneumonia visits
Lifted restrictions
Maintained restrictions
E.
Measles vaccines
Maintained restrictions
Lifted restrictions
F.
Outpatient visits
Maintained restrictions
Lifted restrictions
G.
Diabetes visits
Maintained restrictions
Lifted restrictions
H.
Hypertension visits
Maintained restrictions
Lifted restrictions
I.
HIV Tests
Maintained restrictions
Lifted restrictions
J.
TB cases detected
Maintained restrictions
Lifted restrictions

Title: The effect of lifting COVID-19 restrictions on utilization of primary care services in Nepal: a difference-in-differences analysis

Authors: Kapoor NR, Aryal A, Mehata S, Dulal M, Kruk ME, Bauhoff S, Arsenault C

Supplemental materials

Supplemental Table 1. Additional sources used for COVID-19 restrictions tracking

**Additional Sources:**
48 dists currently under DAO prohibitory order. The Rising Nepal. 2020; published online Aug 31. https://old.risingnepaldaily.com/main-news/48-dists-currently-under-dao-prohibitory-order.
Curfew Relaxed In Kaski, Public Buses Not Plying, Taxis And Private Vehicles Rampant. Arthik Awaj. 2020; published online Aug 28. https://arthikawaj.com/%e0%a4%aa%e0%a5%8b%e0%a4%96%e0%a4%b0%e0%a4%be%e0%a4%ae%e0%a4%be-%e0%a4%a8%e0%a4%bf%e0%a4%b6%e0%a5%87%e0%a4%a7%e0%a4%be%e0%a4%9c%e0%a5%8d%e0%a4%9e%e0%a4%be-%e0%a4%96%e0%a5%81%e0%a4%95%e0%a5%81%e0%a4%b2/.
Four local levels in Jhapa impose weeklong lockdown. Nepal Monitor. 2020; published online Aug 15. https://nepalmonitor.org/reports/view/30662.
Indefinite prohibitory order imposed in Bara. Khabarhub. 2020; published online Aug 17. https://english.khabarhub.com/2020/17/120466/.
INFOGRAPHICS: 48 districts have partial or complete lockdown. myRepublica. 2020; published online Aug 24. http://myrepublica.nagariknetwork.com/news/98460/.
INFOGRAPHICS: These 52 districts are under lockdown. myRepublica. 2020; published online Aug 25. https://myrepublica.nagariknetwork.com/news/infographics-these-52-districts-are-under-lockdown/.
Kathmandu Valley back to lockdown. Nepali Times. 2020; published online Aug 18. https://www.nepalitimes.com/banner/kathmandu-valley-back-to-lockdown/.
Local levels in Kavrepalanchok being sealed off amidst COVID-19 fear. Khabarhub. 2020; published online Aug 15. https://english.khabarhub.com/2020/15/120082/.
Lockdown enforced in Byas Municipality for a week. Nepal Monitor. 2020; published online Aug 17. https://nepalmonitor.org/reports/view/30696.
Lockdown enforced in Dasarathchand Municipality of Baitadi. Nepal Monitor. 2020; published online Aug 20. https://nepalmonitor.org/reports/view/30790.
Lockdown enforced in five local levels of Sindhuli. Nepal Monitor. 2020; published online Aug 15. https://nepalmonitor.org/reports/view/30667.
Koirala N. Lockdown extended for another week in Bhojpur. The Himalayan Times. 2020; published online Sept 10. https://thehimalayantimes.com/nepal/lockdown-extended-for-another-week-in-bhojpur.

For peer review only

For peer review only - http://bmjopen.bmj.com/site/about/guidelines.xhtml

Nepal: Lockdown extended in Kathmandu Valley until September 16. Crisis 24. 2020; published online Sept 10. https://crisis24.garda.com/alerts/2020/09/nepal-lockdown-extended-in-kathmandu-valley-until-september-16-update-24.
Rai MC. Prohibition in 3 cities including Udaipur headquarters. Nagarik News. 2020; published online Aug 12. https://nagariknews.nagariknetwork.com/social-affairs/304061-1597214771.html.
Prohibition in Dang from 14th. Online Khabar. 2020; published online Aug 12. https://www.onlinekhabar.com/2020/08/892463.
Prohibitory order in Dhankuta extended by one week. Khabarhub. 2020; published online Aug 27. https://english.khabarhub.com/2020/27/123095/.
Prohibitory orders in 39 districts to curb COVID-19. The Rising Nepal. 2020; published online Aug 17. https://old.risingnepaldaily.com/mustread/prohibitory-orders-in-39-districts-to-curb-covid-19.
Ten days long lockdown imposed in Sankhuwasabha. Nepal Monitor. 2020; published online Aug 18. https://nepalmonitor.org/reports/view/30747.
Gautam L. Week-long lockdown imposed to prevent spread of coronavirus in Phidim. The Himalayan Times. 2020; published online Aug 28. https://thehimalayantimes.com/nepal/week-long-lockdown-imposed-to-prevent-spread-of-coronavirus-in-phidim.

For peer review only

Supplemental Table 2. Nepal DHIS2 Definitions for health service outcomes

Health service	Nepal DHIS2 Definition
**Outpatient visits**	Disaggregation by Sex & Caste/Ethnicity - Outpatient Cases
**Family planning**	Family Planning Program - Temporary FP Method - Depo-Current User +<br>Family Planning Program - Temporary FP Method - Depo-New Users < 20 Years +<br>Family Planning Program - Temporary FP Method - Depo-New Users > 20 Years +<br>Family Planning Program - Temporary FP Method - Pills- Current User +<br>Family Planning Program - Temporary FP Method - Pills- < 20 Years +<br>Family Planning Program - Temporary FP Method - Pills- > 20 Years +<br>Safe Motherhood Program-Safe Abortion Service-Post Abortion FP Methods Short Term-Medical +<br>Safe Motherhood Program-Safe Abortion Service-Post Abortion FP Methods Short term-Surgical +
**Antenatal care**	Safe Motherhood Program-Antenatal Checkup-First ANC visits (any time) < 20 years +<br>Safe Motherhood Program-Antenatal Checkup-First ANC visits (any time) > 20 years
**Postnatal care**	Safe Motherhood Program- Type of Delivery - 3 PNC visits as per protocol
**Pneumonia**	CBIMCI-(2-59Months)- Classification-ARI-Pneumonia +<br>CBIMCI-(2-59Months)-ORC Classification-ARI-Severe Pneumonia/Very Severe Disease
**Measles**	Immunization program - Children Immunized - Measles/Rubella - 9-11 Months + Immunization program - Children Immunized - Measles/Rubella - 12-23 Months
**HIV tests**	Virology-HIV tests conducted
**TB detection**	Disaggregation by Sex & Caste/Ethnicity- New TB Cases
**Diabetes visits**	Outpatient Morbidity-Nutritional & Metabolic Disorder-Diabetes Mellitus (DM) Cases
**Hypertension visits**	OPD-Morbidity-Cardiovascular & Respiratory Related Problems-Hypertension

Nepal DHIS2 definitions for primary care services.
FP – family planning
ANC – antenatal care
CBIMCI - Community Based Integrated Management of Childhood Illness
HIV – Human immunodeficiency virus
TB – Tuberculosis
OPD – outpatient department

For peer review only

Supplemental Table 3. Level of COVID-19 Restrictions in place from March 14, to September 16, 2020 in Nepal

	Mar 14, 2020 - April 12, 2020 (Chatira 2076)	April 13, 2020 - May 13, 2020 (Baisakh 2077)	May 14, 2020 - June 14, 2020 (Jestha 2077)	June 15, 2020 - July 15, 2020 (Ashar 2077)	July 16, 2020 - August 16, 2020 (Shrawan 2077)	August 17, 2020 - September 16, 2020 (Bhadra 2077)
Stay-at-home required (except essentials)	National[1]	National	National	National	District or palika-specific[3]	District or palika-specific
Business/workplace closures required	National[1]	National	National	National	District or palika-specific[3]	District or palika-specific
Public transport closures	National[1]	National	National	National	District or palika-specific[3]	District or palika-specific
Restricted gatherings to <10	National[1]	National	National	National	National	National
Border closure	National[1]	National	National	National	National	National
School closures	National[1]	National	National	National	National	National
Restrictions on internal movement	National[1]	National	National	National	National	National

[1]These policies were put in place on or around March 22nd, 2020.
[2]These policies were lifted on or around July 22nd, 2020.

Sources:
Rayamajhee B, Pojhrel A, Syangtan G, *et al.* How Well the Government of Nepal Is Responding to COVID-19? An Experience From a Resource-Limited Country to Confront Unprecedented Pandemic. *Front Public Health* 2021; published online Feb 17. https://doi.org/10.3389/fpubh.2021.597808.

Thomas Hale, Noam Angrist, Rafael Goldszmidt, *et al.* A global panel database of pandemic policies policies (Oxford COVID-19 Government Response Tracker). *Nature Human Behaviour* 2021. DOI:10.1038/s41562-021-01079-8.

The Situation of Corona Virus (COVID-19) in Nepal: Daily Reports. INSECOnline. http://inseconline.org/en/covid-19/.

Pradhan TR. Nepal goes under lockdown for a week starting 6am Tuesday. The Kathmandu Post. 2020; published online March 23. https://kathmandupost.com/national/2020/03/23/nepal-goes-under-lockdown-for-a-week-starting-6am-tuesday.

Pradhan TR. Government decides to lift the four-month-long coronavirus lockdown, but with conditions. The Kathmandu Post. 2020; published online July 21. https://kathmandupost.com/national/2020/07/21/government-decides-to-lift-the-four-month-long-coronavirus-lockdown-but-with-conditions.

For peer review only - http://bmjopen.bmj.com/site/about/guidelines.xhtml

Supplemental Table 4. Joint F-tests for parallel trends assessment

	May * Restrictions lifted & June * Restrictions lifted Joint F-test (p-value)
Contraceptive Users	0.43
ANC Visits	0.16
PNC Visits	0.46
Child pneumonia visits	0.29
Measles vaccine	0.24
Outpatient visits	0.32
Diabetes visits	0.52
Hypertension visits	0.11
HIV tests	0.86
TB cases detected	0.16

P-values for joint F-test for *May * Restrictions lifted* and *June* Restrictions lifted* to test they are jointly not significantly different from zero.
May is May 14, 2020 to June 14, 2020 (Jestha 2077) and *June* is June 15, 2020 to July 15, 2020 (Ashar 2077). May and June are in the pre-period, during the national lockdown. The coefficients for *May*Restrictions lifted* and *June*Restrictions lifted* assess if trends are parallel in the pre-period, if the effect of lifting COVID-19 restrictions in palikas is significantly different from the baseline month, April 13 2020 to May 13 2020 (Baisakh 2076).

For peer review only - http://bmjopen.bmj.com/site/about/guidelines.xhtml

Supplemental Table 5. Estimated effect of lifting COVID-19 restrictions on primary care service utilization in Nepal, estimates from difference-in-differences models that exclude March 14, 2020 – April 12, 2020 (Chaitra 2076)

	Restrictions lifted	95% CI	COVID-19 cases	95% CI	N	R^2	adj. R^2
**Contraceptive users**	56.40*	[11.63,101.17]	-0.01	[-0.03,0.01]	2968	0.01	0.01
**ANC Visits**	18.32***	[8.31,28.33]	0.00	[-0.01,0.01]	2900	0.03	0.03
**PNC Visits**	-1.06	[-4.89,2.78]	0.00	[-0.00,0.00]	2076	0.07	0.07
**Child pneumonia visits**	1.45*	[0.11,2.80]	-0.00**	[-0.00,-0.00]	2220	0.03	0.03
**Measles vaccine**	17.60*	[2.69,32.50]	-0.01**	[-0.01,-0.00]	1392	0.08	0.07
**Outpatient visits**	-84.61	[-223.74,54.53]	-0.10***	[-0.13,-0.06]	2968	0.10	0.10
**Diabetes visits**	3.61	[-8.74,15.95]	0.00	[-0.01,0.02]	1332	0.02	0.02
**Hypertension visits**	11.95	[-7.68,31.58]	0.00	[-0.01,0.01]	2756	0.02	0.01
**HIV tests**	33.24	[-11.71,78.20]	0.01	[-0.01,0.03]	944	0.04	0.03
**TB cases detected**	-0.02	[-0.83,0.78]	0.00	[-0.00,0.00]	920	0.05	0.05

95% confidence intervals in brackets
* $p < 0.05$, ** $p < 0.01$, *** $p < 0.001$
The coefficient for *Restrictions lifted* is the effect of lifting COVID-19 restrictions on health service utilization. Models also included fixed effects for month and palikas.

For peer review only

Supplemental Table 6. Number of palika and volume of services in the raw vs. final dataset

		Number of palika reporting			Sum of services		
**Health service**	**Variable name**	**Raw data**	**Final data**	**% difference**	**Raw data**	**Final data**	**% difference**
Contraceptive users	fp_sa_util	753	742	-1%	2,702,347	2,685,227	-1%
Antenatal care	anc_util	752	725	-4%	276,465	271,893	-2%
Postnatal care visits	pnc_util	717	519	-28%	46,846	43,617	-7%
Child pneumonia visits	pneum_util	745	555	-26%	27,164	25,030	-8%
Outpatient visits	opd_util	753	742	-1%	6,866,933	6,828,125	-1%
TB cases detected	tbdetect_qual	679	230	-66%	6,572	4,577	-30%
Measles Vaccine	measles_qual	753	348	-54%	361,213	210,662	-42%
Hypertension visits	hyper_util	752	689	-8%	222,659	220,033	-1%
Diabetes visits	diab_util	627	333	-47%	93,261	89,390	-4%
HIV tests	hivtest_qual	520	236	-55%	227,166	213,789	-6%

For peer review only

STROBE Statement—Checklist of items that should be included in reports of ***cross-sectional studies***

	Item No	Recommendation	Page No
**Title and abstract**	1	(*a*) Indicate the study's design with a commonly used term in the title or the abstract	1, 2
		(*b*) Provide in the abstract an informative and balanced summary of what was done and what was found	2
**Introduction**			
Background/rationale	2	Explain the scientific background and rationale for the investigation being reported	4
Objectives	3	State specific objectives, including any prespecified hypotheses	4,5
**Methods**			
Study design	4	Present key elements of study design early in the paper	6
Setting	5	Describe the setting, locations, and relevant dates, including periods of recruitment, exposure, follow-up, and data collection	6
Participants	6	(*a*) Give the eligibility criteria, and the sources and methods of selection of participants	6,7
Variables	7	Clearly define all outcomes, exposures, predictors, potential confounders, and effect modifiers. Give diagnostic criteria, if applicable	7
Data sources/ measurement	8*	For each variable of interest, give sources of data and details of methods of assessment (measurement). Describe comparability of assessment methods if there is more than one group	6,7
Bias	9	Describe any efforts to address potential sources of bias	7
Study size	10	Explain how the study size was arrived at	6,7
Quantitative variables	11	Explain how quantitative variables were handled in the analyses. If applicable, describe which groupings were chosen and why	6,7
Statistical methods	12	(*a*) Describe all statistical methods, including those used to control for confounding	7
		(*b*) Describe any methods used to examine subgroups and interactions	7
		(*c*) Explain how missing data were addressed	7
		(*d*) If applicable, describe analytical methods taking account of sampling strategy	7
		(*e*) Describe any sensitivity analyses	7
**Results**			
Participants	13*	(a) Report numbers of individuals at each stage of study—eg numbers potentially eligible, examined for eligibility, confirmed eligible, included in the study, completing follow-up, and analysed	9, 17
		(b) Give reasons for non-participation at each stage	9
		(c) Consider use of a flow diagram	NA
Descriptive data	14*	(a) Give characteristics of study participants (eg demographic, clinical, social) and information on exposures and potential confounders	9, 17, 18
		(b) Indicate number of participants with missing data for each variable of interest	9,17
Outcome data	15*	Report numbers of outcome events or summary measures	9,17
Main results	16	(*a*) Give unadjusted estimates and, if applicable, confounder-adjusted estimates and their precision (eg, 95% confidence interval). Make clear which confounders were adjusted for and why they were included	9, 17, 20

For peer review only - http://bmjopen.bmj.com/site/about/guidelines.xhtml

		(*b*) Report category boundaries when continuous variables were categorized	NA
		(*c*) If relevant, consider translating estimates of relative risk into absolute risk for a meaningful time period	9
Other analyses	17	Report other analyses done—eg analyses of subgroups and interactions, and sensitivity analyses	9
**Discussion**			
Key results	18	Summarise key results with reference to study objectives	10
Limitations	19	Discuss limitations of the study, taking into account sources of potential bias or imprecision. Discuss both direction and magnitude of any potential bias	11
Interpretation	20	Give a cautious overall interpretation of results considering objectives, limitations, multiplicity of analyses, results from similar studies, and other relevant evidence	10, 11
Generalisability	21	Discuss the generalisability (external validity) of the study results	11, 12
**Other information**			
Funding	22	Give the source of funding and the role of the funders for the present study and, if applicable, for the original study on which the present article is based	12

*Give information separately for exposed and unexposed groups.

Note: An Explanation and Elaboration article discusses each checklist item and gives methodological background and published examples of transparent reporting. The STROBE checklist is best used in conjunction with this article (freely available on the Web sites of PLoS Medicine at http://www.plosmedicine.org/, Annals of Internal Medicine at http://www.annals.org/, and Epidemiology at http://www.epidem.com/). Information on the STROBE Initiative is available at www.strobe-statement.org.

For peer review only

BMJ Open

The effect of lifting COVID-19 restrictions on utilization of primary care services in Nepal: a difference-in-differences analysis

Journal:	*BMJ Open*
Manuscript ID	bmjopen-2022-061849.R2
Article Type:	Original research
Date Submitted by the Author:	19-Oct-2022

Complete List of Authors:	Kapoor, Neena ; Harvard University T H Chan School of Public Health, Department of Global Health and Population<br>Aryal, Amit; Swiss Tropical and Public Health Institute<br>Mehata, Suresh; Government of Nepal Ministry of Health and Population<br>Dulal, Mahesh; Office of Member of Federal Parliament Nepal, Gagan Kumar Thapa<br>Kruk, Margaret; Harvard University T H Chan School of Public Health, Department of Global Health and Population<br>Bauhoff, Sebastian; Harvard University T H Chan School of Public Health, Department of Global Health and Population<br>Arsenault, C; Harvard University T H Chan School of Public Health, Department of Global Health and Population

**Primary Subject Heading**:	Health services research
Secondary Subject Heading:	Health policy, Public health, Global health
Keywords:	COVID-19, Health policy < HEALTH SERVICES ADMINISTRATION & MANAGEMENT, PRIMARY CARE

SCHOLARONE™
Manuscripts

I, the Submitting Author has the right to grant and does grant on behalf of all authors of the Work (as defined in the below author licence), an exclusive licence and/or a non-exclusive licence for contributions from authors who are: i) UK Crown employees; ii) where BMJ has agreed a CC-BY licence shall apply, and/or iii) in accordance with the terms applicable for US Federal Government officers or employees acting as part of their official duties; on a worldwide, perpetual, irrevocable, royalty-free basis to BMJ Publishing Group Ltd ("BMJ") its licensees and where the relevant Journal is co-owned by BMJ to the co-owners of the Journal, to publish the Work in this journal and any other BMJ products and to exploit all rights, as set out in our licence.

The Submitting Author accepts and understands that any supply made under these terms is made by BMJ to the Submitting Author unless you are acting as an employee on behalf of your employer or a postgraduate student of an affiliated institution which is paying any applicable article publishing charge ("APC") for Open Access articles. Where the Submitting Author wishes to make the Work available on an Open Access basis (and intends to pay the relevant APC), the terms of reuse of such Open Access shall be governed by a Creative Commons licence – details of these licences and which Creative Commons licence will apply to this Work are set out in our licence referred to above.

Other than as permitted in any relevant BMJ Author's Self Archiving Policies, I confirm this Work has not been accepted for publication elsewhere, is not being considered for publication elsewhere and does not duplicate material already published. I confirm all authors consent to publication of this Work and authorise the granting of this licence.

For peer review only

The effect of lifting COVID-19 restrictions on utilization of primary care services in Nepal: a difference-in-differences analysis

Authors: Kapoor NR[1*], Aryal A[2], Mehata S[3], Dulal M[4], Kruk ME[1], Bauhoff S[1], Arsenault C[1]

1. Department of Global Health and Population, Harvard T.H. Chan School of Public Health, Boston, USA
2. Swiss Tropical and Public Health Institute, University of Basel, Basel, Switzerland
3. Ministry of Health and Population, Government of Nepal, Kathmandu, Nepal
4. Office of Member of Federal Parliament Nepal, Gagan Kumar Thapa, Kathmandu, Nepal

***Corresponding Author:**
655 Huntington Ave, Boston MA, USA
nkapoor@hsph.harvard.edu

For peer review only

Abstract

Introduction An increasing number of studies have reported disruptions in health service utilization due to the COVID pandemic and its associated restrictions. However, little is known about the effect of lifting COVID restrictions on health service utilization. The objective of this study was to estimate the effect of lifting COVID restrictions on primary care service utilization in Nepal.

Methods Data on utilization of 10 primary care services were extracted from the Health Management Information System (HMIS) across all health facilities in Nepal. We used a difference-in-differences design and linear fixed effects regressions to estimate the effect of lifting COVID restrictions. The treatment group included palikas that had lifted restrictions in place from August 17 to September 16, 2020 (Bhadra 2077) and the control group included palikas that had maintained restrictions during that period. The pre-period included the four months of national lockdown from March 24 to July 22, 2020 (Chaitra 2076-Ashar 2077). Models included month and palika fixed effects and controlled for COVID incidence.

Results We found that lifting COVID restrictions was associated with an average increase per palika of 57.5 contraceptive users (95% CI 14.6, 100.5), 15.6 antenatal care visits (95% CI 5.3, 25.9), and 1.6 child pneumonia visits (95% CI 0.2, 2.9). This corresponded to a 9.4% increase in contraceptive users, 34.2% increase in antenatal care visits, and 15.6% increase in child pneumonia visits. Utilization of most other primary care services also increased after lifting restrictions, but coefficients were not statistically significant.

Conclusions Despite the ongoing pandemic, lifting restrictions can lead to an increase in some primary care services. Our results point to a causal link between restrictions and health service utilization and call for policy makers in low- and middle-income countries to carefully consider the tradeoffs of strict lockdowns during future COVID waves or future pandemics.

For peer review only

For peer review only - http://bmjopen.bmj.com/site/about/guidelines.xhtml

Strengths and limitations of this study

- We included data on 10 wide-ranging primary care services extracted from the Nepal Health Management Information System (HMIS).
- We used a difference-in-differences (DID) design to compare service use in palikas that lifted restrictions to those that maintained them, which controls for time-fixed differences between palikas and temporal trends common to both groups.
- We controlled for new COVID cases at the district level, but other time-varying confounders could affect the two groups differently.
- HMIS data provide real-time information on patterns in service use however, despite the data cleaning conducted, data quality issues and underreporting by some facilities could bias our results.

For peer review only

Background

In a time of crisis, high quality health systems have two tasks: respond to the crisis and maintain the provision and quality of essential health services.[1] Health systems in low-income countries that may already be under-funded, under-resourced, and over-burdened, may be particularly vulnerable during the COVID pandemic. An increasing number of studies have reported disruptions in health service utilization since the start of the pandemic in low- and middle-income countries (LMICs).[2]–[10] The ongoing COVID pandemic has directly strained health care systems around the world that are struggling to meet the physical resource, human resource (numbers and skills), and service coordination demands of the pandemic. The pandemic may also have had indirect effects on primary health care utilization, as restrictions and lockdowns implemented by governments to reduce the spread of COVID may affect people's ability or willingness to visit healthcare facilities.

Nepal is a lower-middle income country of South Asia with a population of 28.6 million.[11] The country has shown significant gains in health and health care utilization over the past decade. The pandemic could reverse these hard-won gains. As of December 2021, COVID-19 had infected more than 800,000 people across Nepal and had led to a reported 11,594 deaths.[12] Despite the existence of effective vaccines, only 32.8% of the Nepali population is currently fully vaccinated against COVID.[12]

Following the declaration of the pandemic on March 11, 2020 by the World Health Organization, health care utilization declined substantially in Nepal, ranging from a 65% decline in tuberculosis (TB) case detection to a 4% decline in contraceptive use.[3] Many factors may be responsible for a decline in health service utilization during the pandemic. Declines may stem from the pandemic itself (perceived threat), the actual number of new COVID cases reported in a given period (leading to a fear of infection when visiting facilities or to overburdened health facilities treating COVID patients) or from the restrictions imposed (i.e., lockdowns) to curb the spread of COVID. The barriers imposed by COVID restrictions, such as stay-at home requirements or public transport closures, may play an important role in affecting health care utilization. In Madesh Pradesh (formerly known as Province 2 of Nepal), people reported that the national lockdown restricted accessibility to health facilities and deterred them from seeking care.[13] An increasing number of studies have described the effects of the pandemic and associated restrictions on health care utilization.[2]–[10] However, little is known about the effect of lifting COVID restrictions on health care utilization. Understanding these effects is crucial to plan for potential rebounds in demand and determining whether potentially weakened health systems can cope with surges in demand.

In the wake of the COVID pandemic, in March 2020, the Government of Nepal implemented a country-wide lockdown.[14], [15] After almost four months of strict lockdown, in July 2020, the decision was made to end the national lockdown and lift most of these restrictions at the national level.[16] However, some of Nepal's municipality governments decided to maintain restrictions to contain the spread of COVID. This contrast in removal of restrictions gave rise to a natural experiment that allowed us to estimate the causal effect of lifting COVID restrictions on health care utilization.

For peer review only - http://bmjopen.bmj.com/site/about/guidelines.xhtml

86 In this study, we used a difference-in-differences design (DID) to estimate the effect of lifting COVID restrictions on primary care service utilization in Nepal. Understanding the effect of lifting COVID restrictions on primary health care is crucial to inform policy responses during future waves of COVID or future pandemics in low- and middle-income countries.

For peer review only

For peer review only - http://bmjopen.bmj.com/site/about/guidelines.xhtml

Methods

Data sources

We used data from the Nepal Health Management Information System (HMIS) obtained through the DHIS2 platform. The HMIS in Nepal includes information from all health facilities in the country including both public and private facilities across all levels of the health system.[17] A total of 7,605 health facilities are expected to report to the DHIS2 across 753 urban and rural municipalities known as "palikas" (*palikas are a local form of government in Nepal's federal system)*.

Information on the types of COVID-19 restrictions in place was obtained from various sources including: INSECOnline, a human rights news portal in Nepal providing daily COVID updates, the Nepal COVID Crisis Management Coordination Center (CCMCC) government sites, District Administration Office (DAO) sites and additional online news sources (**supplementary material table 1**).[18]–[20]

We also included data on the total number of COVID cases at district level in Nepal (COVID case counts were not available at the palika level). Monthly COVID cases in each of the 77 districts were obtained from the Nepal Health Emergency Operation Centre, Ministry of Health and Population.[21]

Measures

Primary care service utilization

We aimed to include 12 primary care services: contraceptive users, antenatal care (ANC) visits, postnatal care (PNC) visits, visits for children under five with pneumonia, visits for children under five with diarrhea, pentavalent vaccinations, measles vaccinations, visits for diabetes, visits for hypertension, number of human immunodeficiency virus (HIV) tests conducted, number of tuberculosis (TB) cases detected and total outpatient visits. Selection of these services was based on availability in the DHIS2 and because they covered a range of health needs including sexual, reproductive, maternal, newborn, child, and adolescent health (SRMNCAH) services, infectious diseases, and non-communicable diseases. Detailed definitions are in **supplementary material table 2.**

We obtained the monthly number of each of these services provided from January 15, 2019, to January 13, 2021 (Nepali calendar Magh 2077 to Poush 2077). These data were available at the palika level.

Because DHIS2 data are self-reported by health facilities, these data may contain errors. Our data cleaning procedures entailed identifying positive outliers (greater than 3.5 standard deviations from the mean trend) and setting any outliers as missing.[22] We did not assess negative outliers since decreases in utilization were expected during the lockdown period. For each health service, we also excluded palikas that were missing any data during the five-month study period (a complete case analysis).

For peer review only - http://bmjopen.bmj.com/site/about/guidelines.xhtml

COVID-19 restrictions

From March 24 to July 22, 2020, the Federal Government of Nepal imposed a strict nation-wide lockdown in response to the pandemic. This included stay-at-home requirements except for essential services, businesses, public transport and school closures, and restrictions on large gatherings, international travel, and internal movement (see **supplementary material table 3**). On July 22, major restrictions were lifted at the national level, including stay-at-home requirements, non-essential business, and public transport closures, but some districts and palikas maintained these restrictions. Following the lifting of the national lockdown, 248 palikas lifted the restrictions while 505 palikas maintained one or more of these restrictions.

For this analysis, the treatment group includes the palikas that lifted these restrictions, while the control group includes palikas that continued at least one or more restriction. The pre-intervention period includes the four months from March 14 to July 15 (which corresponds to the Nepali months of Chaitra 2076 to Ashar 2077) and the post-intervention period is August 17 to September 16, 2020 (the Nepali month of Bhadra 2077). July 16th through August 16th, 2020 (Shrawan 2077) was excluded from the analysis since lifting of the national lockdown occurred mid-month. Our analysis used Nepal calendar months as the unit of time.

The classification of palikas into treated and control groups was done using primarily INSEConline, an online news portal that provided daily updates on the COVID-19 situation in Nepal.[18] Four of the co-authors extracted information on the types of restrictions in place in each palika from the INSEConline news reports and verified and complemented the information with CCMCC government sites, DAO sites, and additional news sources (**supplementary material table 1**).[19], [20] Any disagreements were resolved through discussion. We used a 10-day threshold as a general rule of thumb. If restrictions were in place for less than 10 days during the month, the palika was classified as having lifted the restrictions and was included in the treatment group. If the restrictions covered more than 10 days, the palika remained in the control group (maintained restrictions). However, given imprecision in some of the policy reports, it was not always possible to apply this threshold with precision in some palikas.

Figure 1 shows the timeline of COVID restrictions and cases in Nepal from January 1, 2020, to September 16, 2020. The first COVID case was reported in Nepal on January 25, 2020. Notably, the end of the national lockdown on July 22, 2020 coincided with the beginning of the first real COVID wave (**figure 1**).

Statistical analysis

The analysis was conducted at the palika level, using Nepali calendar months as the unit of time. DID analysis is often used in policy evaluations to compare outcomes before and after a policy change for a group affected by the change (treated group) to a group not affected by the change (control group).[23] We used a DID design and fixed effects ordinary least square regression models. The following model was used and repeated for each of the health services analyzed:

$$S_{pt} = \alpha + \beta[lifted\ lockdown_{pt}] + \gamma_t + \delta_p + X_{dt} + \varepsilon_{pt}$$

For peer review only - http://bmjopen.bmj.com/site/about/guidelines.xhtml

Where S_{pt} is the number of health services (number of visits or users) provided in palika p in month t, γ_t and δ_p are vectors of month and palika fixed effects, respectively, and X_{dt} is the number of new COVID cases in district d and month t. The coefficient of interest is β, which represents the difference in service utilization among palikas that lifted restrictions compared to those that maintained restrictions. The palika fixed effects controls for time-invariant differences between palikas and avoids the need to control for time-fixed confounders. For example, the palika fixed effects will control for unmeasured differences between palikas (urbanicity, population size, wealth) that can affect service utilization. The DID design also controls for all factors commonly affecting the outcomes in all palikas over time, through month fixed effects. COVID incidence would be associated with both the exposure (restrictions), and the outcome (health service utilization) and may vary between the treatment and control groups. Thus, we included monthly COVID cases in the regression models to control for potential confounding. Models also included clustered standard errors at the palika level.

A main assumption of DID models is that the outcome trend in the control group represents a good approximation of what the outcome trend would have been in the treatment group in the absence of the policy change (i.e., the counterfactual trend). Thus, to probe the assumption that the control palika trends were a good counterfactual for the treatment group (palikas that lifted restrictions), we implemented a series of tests. First, we conducted a pre-trend placebo test by comparing the difference in service utilization between the treated and control palikas in May and June 2020 (Jestha and Ashar 2077) compared to April 2020 (Baisakh 2077). We performed a joint F test of whether these coefficients were jointly zero (**supplementary materials**). Since all palikas were under the same restrictions in March to June 2020 (Chaitra 2076 to Ashar 2077), there should be no effect, providing evidence for parallel trends in the pre-period. Second, we assessed the parallel trend assumption graphically (**figure 2**). We also conducted a sensitivity analysis, excluding March 14 to April 12, 2020 (Chaitra 2076) from the analysis, since the national lockdown was put in place in the middle of this month, to see if the results differed. This research was approved by the Nepal Health Research Council (NHRC), reference number 650, and determined to be exempt from a full review by the Institutional Review Board (IRB) of the Harvard T.H. Chan School of Public Health.

Patient and public involvement

Patients will be involved in dissemination of this research. There was no patient or public involvement in the design, reporting, or interpretation of results.

For peer review only

For peer review only - http://bmjopen.bmj.com/site/about/guidelines.xhtml

Results

The average number of services provided during the national lockdown (pre-intervention period) and after the national lockdown was lifted (post-intervention period) for treated and control palikas are shown in **Table 1**. Table 1 also shows the number of palikas included in the analysis of each health service. Service utilization tended to be lower in the treatment group for most services. The palikas in the treatment group, those that lifted restrictions, had fewer COVID cases and smaller populations, on average, compared to the palikas in the control group. These differences are accounted for by the palika fixed effects in the DID design.

Table 1: Number and characteristics of palikas by treatment group and period

	**Treatment group (lifted restrictions)**		**Control group (maintained restrictions)**	
	**Pre-period**	**Post-period**	**Pre-period**	**Post-period**
	Average per month (N)	Average per month (N)	Average per month (N)	Average per month (N)
**Contraceptive users**	614.09 (245)	646.10 (245)	780.52 (497)	751.41 (497)
**Antenatal care visits**	45.66 (235)	48.97 (235)	90.57 (490)	81.52 (490)
**Postnatal care visits**	12.49 (182)	18.65 (182)	16.93 (337)	24.63 (337)
**Child pneumonia visits**	9.92 (186)	10.98 (186)	8.59 (369)	7.95 (369)
**Measles Vaccine**	84.47 (102)	75.28 (102)	140.83 (246)	121.74 (246)
**Outpatient visits**	1489.23 (243)	1870.75 (243)	1896.25 (499)	2286.77 (499)
**Diabetes visits**	46.66 (100)	57.04 (100)	54.27 (233)	62.00 (233)
**Hypertension visits**	53.43 (220)	72.44 (220)	65.50 (469)	72.93 (469)
**HIV tests**	87.55 (68)	125.88 (68)	212.92 (168)	228.17 (168)
**TB cases detected**	3.07 (52)	3.13 (52)	4.25 (178)	4.20 (178)
**Average number of new COVID cases over the study period per palika**	287		1,004	
**Average palika population size**	23,649		46,007	

The average number of health visits per palika and month. The treatment group includes the palikas that lifted restrictions in August 17 to September 16, 2020 (Bhadra 2077). The control group includes those that maintained restrictions during that month. The (N) is the number of palikas reporting each month in the period for that service. National lockdown period (pre-period) includes March 13 to July 16, 2020 (Chaitra 2076 to Ashar 2077). The post-period includes August 17 to September 16, 2020 (Bhadra 2077). The average number of new COVID cases in each period is COVID cases at the district level since these data were not available at the palika level. The palika population size data was obtained from the Preliminary Data of National Population and Housing Census 2021.[24]

Figure 2 shows the trend in primary care service utilization from January 2019 to September 2020 (equivalent to Magh 2075 to Bhadra 2077) and reveals parallel trends before and during the national lockdown period (our pre-period) for all services included. A sharp decrease in utilization is observed in both groups of palikas at the start of the pandemic when the national lockdown was put in place (months 14 to 15 in **figure 2**). For most services, this decline was

For peer review only - http://bmjopen.bmj.com/site/about/guidelines.xhtml

followed by a gradual resumption in the pre-period. Given these similar trends in both groups, the control palikas appear to provide appropriate counterfactual trends in the post-period. The joint F-test (**supplementary material table 4**) also did not reject the null hypothesis that the outcomes evolved differently in the treated versus the control palikas in the pre-period for the 10 services included. The parallel trend assumption was violated for two health services: visits for children under five with diarrhea and pentavalent vaccinations, which were excluded from the analysis.

Table 2. Estimated effect of lifting COVID restrictions on primary care service utilization in Nepal, estimates from difference-in-differences models

	Restrictions lifted	95% CI	COVID cases	95% CI	*N*	R^2	adj. R^2
**Contraceptive users**	57.51**	[14.55,100.48]	-0.01	[-0.02,0.01]	3710	0.01	0.01
**ANC visits**	15.60**	[5.34,25.86]	0.01	[-0.01,0.02]	3625	0.08	0.07
**PNC visits**	-1.50	[-4.94,1.94]	0.00	[-0.00,0.00]	2595	0.07	0.07
**Child pneumonia visits**	1.55*	[0.24,2.86]	-0.00**	[-0.00,-0.00]	2775	0.19	0.19
**Measles vaccine**	7.35	[-6.49,21.19]	0.00	[-0.01,0.00]	1740	0.20	0.20
**Outpatient visits**	-56.81	[-193.77,80.16]	-0.10***	[-0.13,-0.06]	3710	0.09	0.09
**Diabetes visits**	5.01	[-8.12,18.14]	0.00	[-0.01,0.02]	1665	0.03	0.02
**Hypertension visits**	12.70	[-6.74,32.14]	0.00	[-0.01,0.01]	3445	0.02	0.02
**HIV tests**	34.83	[-12.57,82.22]	0.02	[-0.02,0.05]	1180	0.04	0.04
**TB cases detected**	0.06	[-0.73,0.85]	0.00	[-0.00,0.00]	1150	0.04	0.04

95% confidence intervals in brackets
* $p < 0.05$, ** $p < 0.01$, *** $p < 0.001$
The coefficient for *Restrictions lifted* is the effect of lifting COVID restrictions on health service utilization. Models also included fixed effects for month and palika.

Estimates from DID regressions are reported in **Table 2** for the 10 health services. The coefficient for *restrictions lifted* is the DID estimate and can be interpreted as the difference in adjusted service utilization between the treatment (lifted restrictions) and control (maintained restrictions) palikas in the post-period.

Lifting COVID restrictions led to a positive increase in all services except total outpatient visits and postnatal care visits. These effects were statistically significant for three services. Lifting restrictions led to an average increase per palika of 57.5 contraceptive users (95% Confidence Interval (CI) 14.6-100.5), 15.6 antenatal care visits (95% CI 5.3-25.9), and 1.6 child pneumonia visits (95% CI 0.2-2.9). Compared to the pre-COVID average utilization, this represented a 9.4% increase in contraceptive use, 34.2% increase in antenatal care visits and a 15.6% increase in child pneumonia visits.

Similarly, although not statistically significant, lifting restrictions led to 7.4 more children vaccinated against measles (95% CI -6.5-21.2), 5.0 more diabetes visits (95% CI -8.1-18.1), 12.7 more hypertension visits (95% CI -6.7-32.1), 34.8 additional HIV tests (95% CI -12.6-82.2) and

For peer review only - http://bmjopen.bmj.com/site/about/guidelines.xhtml

0.1 additional TB cases detected (95% -0.7-0.9) on average per palika. These increases were equivalent to increases of 8.7% for measles vaccinations, 10.7% for diabetes visits, 23.8% for hypertension visits, 39.8% for HIV tests, and 2.0% for TB case detection in palikas that lifted restrictions compared to those that maintained them. In contrast, the coefficient for PNC and total outpatient visits were negative but these were not statistically significant. They were equivalent to declines of 12.0% fewer PNC visits and 3.8% fewer outpatient visits in palikas that lifted restrictions compared to those that did not. Results from the sensitivity analysis that excluded March 14 to April 12, 2020 (Chaitra 2076) were largely consistent with the main model, with the exception of measles vaccinations which had a statistically significant increase in palikas that lifted restrictions (see **supplementary material table 5**).

For peer review only

Discussion

In this analysis, we used HMIS data and a difference-in-differences design to estimate the effect of lifting COVID restrictions on primary care health service utilization in Nepal. We found that lifting restrictions increased contraceptive use, antenatal care, and sick child visits by 9.4% to 34.2% on average across palikas. Utilization of most other primary care services also increased by 2.0% to 39.8% but were not statistically significant. These results provide evidence that COVID restrictions are linked to primary care service utilization in Nepal and that lifting these restrictions can lead to an increase in service uptake. To our knowledge, this is the first paper to estimate the effect of lifting COVID restrictions on health service utilization using a quasi-experimental method.

There are many mechanisms through which COVID-related restrictions (stay-at-home requirements, business/workplace closures and public transport closures) might affect primary health care utilization. People in Nepal have reported that public transport closures during the national lockdown prevented them from reaching healthcare facilities.[13] In addition, stay-at-home requirements meant that people were only permitted to leave their home for essential services. Although essential services included healthcare, stay-at-home requirements were firmly enforced by law enforcement officials, and individuals were arrested and jailed or fined if they defied them.[14], [25] Knowing this risk, people may have been deterred from seeking healthcare, despite being allowed to do so. It is also possible that many people did not know that visiting health facilities was allowed during the lockdown. The strict lockdown may have also increased anxieties around COVID and deterred people from seeking care.[13], [26] Once COVID restrictions were lifted, these barriers would be subdued, and an increase in utilization would be expected.

Lockdown policies, primarily stay-at-home requirements and business or workplace closures, can also have detrimental economic effects, potentially pushing low-income individuals and families further into poverty.[14], [27] Most of the Nepali population works in the informal sector, including a large number in the tourism industry, which was severely impacted by the pandemic. Although the government developed economic support packages, informal sector workers or other marginalized groups often did not benefit from these.[14] Essential health services are supposed to be free of charge in public facilities in Nepal.[28] However, Nepalis often incur costs at point of service, with more than half of health expenditures being made out-of-pocket.[28], [29] In addition, between 20% and 61% of people use private sector facilities for primary care in Nepal, depending on the type of service.[28], [30], [31] Lifting COVID restrictions could have had an immediate effect on people's ability to generate income, especially for those working in the informal sector, and may have allowed them to pay for health care costs and thus seek health services again.

In the present study, we found statistically significant effects only for reproductive, maternal and child health services (RMNCH): contraceptive visits, antenatal care, and child pneumonia visits. Both historically and during the pandemic, the government of Nepal has emphasized the promotion and improvement of RMNCH services. During COVID, RMNCH services were carefully monitored and mapped by Nepal's government to detect and address potential declines

For peer review only - http://bmjopen.bmj.com/site/about/guidelines.xhtml

in coverage. The Country Preparedness and Response Plan focused heavily on maintaining RMNCH services and interim guidelines for RMNCH were also endorsed.[32] To our knowledge, no similar guidelines were issued for other primary care services. These important RMNCH-focused efforts might explain why effects were only statistically significant for these services and not for other, less promoted, services like non-communicable diseases.[32] For example, policymakers suspended Measles-Rubella vaccinations campaigns, but aware of the risk of outbreaks, decided to continue the campaign after only one month. Nonetheless, we found no impact of lifting COVID restrictions on facility-based PNC visits. This could be due to a large program for PNC outreach (home visits) which was launched shortly before the pandemic and continued during the lockdown in some districts. This could explain why there was no difference in facility-based PNC between palikas that lifted restrictions and those that maintained them.

Other studies have shown that the declaration of the pandemic and the implementation of restrictions led to important declines in health service utilization of varying magnitude and duration in many countries.[2]–[10] Studies from Nepal showed declines in primary care and hospital-based care following the implementation of COVID restrictions including fewer deliveries and a potential increase in neonatal mortality and institutional stillbirths.[3], [8] In contrast, our study assessed the effect of lifting these restrictions, and the resulting increase in service use using a DID design. DID designs compare trends between a treatment and comparison group and compare each group to itself, estimating an average of the counterfactual DID contrasts.

Nonetheless, our study has limitations. The DID design controls for time fixed differences between palikas (such as population size) and for secular trends affecting all groups. However, it is possible that remaining time-varying confounders affected the two groups differently. For example, although we adjusted for COVID caseloads at the district level, it is possible that palika-specific outbreaks influenced the decision to maintain restrictions. Another limitation relates to the potential for measurement error for both the restrictions and the health service utilization outcomes. The exposure variable may have been misclassified due to missing information on the restrictions in place in palikas. Although multiple sources were reviewed to collect and confirm the implementation of these restrictions, these sources sometimes lacked precision. Any misclassification due to missing information would have likely resulted in the palika mistakenly included in the treatment group (as having lifted the restrictions). This would bias the results towards the null. DHIS2 data are self-reported by facilities and may also contain errors, and reporting quality may have been affected by the pandemic. However, positive outliers were removed and only facilities that reported each indicator each month during the study period were included. It is unclear whether DHIS2 data quality issues would affect our analysis since misreporting should be similar in both the treatment and control groups. The estimates for TB case detection and measles vaccination must also be interpreted with caution as an important number of observations were excluded by the complete case analysis (see **supplementary material table 6**). In addition, our study was limited by the type of data available in the Nepal HMIS. For example, other important primary care services are not collected in the HMIS, such as mental health visits, which might have been affected by the pandemic. The Nepal HMIS also does not include data on home-visits by community health volunteers, which may be why we did not detect an increase in postnatal care visits, as described earlier. In addition, the Nepal HMIS only contains information aggregated at the health facility level, and we are unable to describe

For peer review only - http://bmjopen.bmj.com/site/about/guidelines.xhtml

patient characteristics and demographic information. Finally, the outcome data was only available monthly, and the beginning and end of restrictions did not always match DHIS2 months precisely. Thus, policy dates and outcomes were not perfectly matched. In our dataset, the pre-period began 10 days before the national lockdown. However, sensitivity analyses that excluded the first month of the lockdown showed similar results (**supplementary material table 5**).

Our results have important implications for policy. We found that despite the ongoing COVID pandemic, lifting restrictions can lead to an increase in RMNCH service utilization. Universal utilization of these services is crucial to improve health outcomes. Antenatal care visits are essential to identify conditions that might threaten the mother or newborn's health.[33] It is estimated that a 10% decrease in coverage of pregnancy related and newborn health care during COVID-19 could result in an additional 28,000 maternal deaths and 168,000 neonatal deaths globally.[34] In addition, reduced contraceptive use could results in an increase in unintended pregnancies which can also place both the pregnant person and child at risk.[35] Delayed care for respiratory illnesses during COVID restrictions could increase the incidence of pneumonia. Pneumonia is one of the leading causes of death for children under five, and missed care could further exacerbate this burden.[36] Nonetheless, it is important to note that an increase in child pneumonia visits after restrictions were lifted could be linked to an increase in needs from further spread of respiratory illnesses (including COVID and non-COVID) rather than from pent up demand.

Our study contributes to the literature on the indirect effects of COVID-19 restrictions on health systems. Although effective vaccines are now available, few people in LMICs are fully immunized against COVID due to widespread inequities in access to vaccines.[37] Future waves of COVID infections and emerging variants are likely to push governments to consider re-implementing temporary restrictions and lockdowns. At the start of the pandemic, many countries took a one-size-fits-all approach with COVID containment policies, as there was understandably much uncertainty surrounding COVID and its effects. As we gain insight into the indirect effects of these restrictions, it is important that policy-makers tailor these policies to their own demographic, disease, and sociocultural contexts, and prepare health systems to respond accordingly.[27] Policy makers should consider strategies to promote and maintain all types of primary care services during future waves of COVID and future pandemics. Such strategies may include better risk communication on the importance of essential health care and alternative service delivery modes such as telemedicine or differentiated service delivery strategies.[38] Health facilities should also be prepared to face potential increases in demand for health care when restrictions are eased. Strengthening community health centers and public primary care services in Nepal is needed, including improving quality of care, and promoting better resilience during shocks.

Contribution statement

NRK, CA, SB and MEK designed the study. NRK, CA, SM, AA, and MD compiled and verified the data. NRK and CA led the data analysis. NRK wrote the first draft and all other co-authors, contributed to the interpretation of findings, and read, improved and approved the final manuscript.

For peer review only - http://bmjopen.bmj.com/site/about/guidelines.xhtml

Funding

This work is part of the Grand Challenges ICODA pilot initiative, delivered by Health Data Research UK and funded by the Bill & Melinda Gates Foundation and the Minderoo Foundation (reference number 2021.0091). We also acknowledge funding from the Bill and Melinda Gates Foundation (grant INV-005254).

Competing interest

There are no competing interests for any authors.

Ethics approval

This study does not involve human participants.

Data availability

Data may be obtained from a third party and are not publicly available.

For peer review only

References:

[1] Kruk, M. E. *et al.*, "High-quality health systems in the Sustainable Development Goals era: time for a revolution," *Lancet Glob Health*, vol. 6, no. 11, pp. e1196–e1252, Nov. 2018, doi: 10.1016/S2214-109X(18)30386-3.

[2] Moynihan, R *et al.*, "Impact of COVID-19 pandemic on utilisation of healthcare services: a systematic review," *BMJ Open*, vol. 11, no. 3, Mar. 2021, doi: 10.1136/bmjopen-2020-045343.

[3] C. Arsenault *et al.*, "COVID-19 and resilience of healthcare systems in 10 countries," *Nat Med*, vol. 28, pp. 1314–1324, Mar. 2022, doi: 10.1038/s41591-022-01750-1.

[4] Hategeka, C *et al.*, "Impact of the COVID-19 pandemic and response on the utilisation of health services in public facilities during the first wave in Kinshasa, the Democratic Republic of the Congo," *BMJ Glob Health*, vol. 6, no. 7, Jul. 2021, [Online]. Available: https://www.ncbi.nlm.nih.gov/pubmed/34315776

[5] Doubova, SV, Leslie, HH, Kruk, ME, Perez-Cuevas, R, and Arsenault, C, "Disruption in essential health services in Mexico during COVID-19: an interrupted time series analysis of health information system data," *BMJ Glob Health*, vol. 6, no. 9, doi: 10.1136/bmjgh-2021-006204.

[6] Burt, JF *et al.*, "Indirect effects of COVID-19 on maternal, neonatal, child, sexual and reproductive health services in Kampala, Uganda," *BMJ Glob Health*, vol. 6, no. 8, Aug. 2021, doi: 10.1136/bmjgh-2021-006102.

[7] Z. Aranda *et al.*, "Disruptions in maternal health service use during the COVID-19 pandemic in 2020: experiences from 37 health facilities in low-income and middle-income countries," *BMJ Global Health*, vol. 7, no. 1, p. e007247, Jan. 2022, doi: 10.1136/bmjgh-2021-007247.

[8] A. KC *et al.*, "Effect of the COVID-19 pandemic response on intrapartum care, stillbirth, and neonatal mortality outcomes in Nepal: a prospective observational study," *The Lancet Global Health*, vol. 8, no. 10, pp. e1273–e1281, Oct. 2020, doi: 10.1016/S2214-109X(20)30345-4.

[9] G. Shapira *et al.*, "Disruptions in maternal and child health service utilization during COVID-19: analysis from eight sub-Saharan African countries," *Health Policy Plan*, vol. 36, no. 7, pp. 1140–1151, Aug. 2021, doi: 10.1093/heapol/czab064.

[10] T. Ahmed *et al.*, "Indirect Effects on Maternal and Child Mortality from the COVID-19 Pandemic: Evidence from Disruptions in Healthcare Utilization in 18 Low- and Middle-Income Countries." Rochester, NY, Sep. 09, 2021. doi: 10.2139/ssrn.3916767.

[11] "The World Bank Data," 2019. [Online]. Available: https://data.worldbank.org/country/nepal

[12] "Our World in Data," 2021. [Online]. Available: https://ourworldindata.org/coronavirus/

[13] Singh, DR *et al.*, "Impact of COVID-19 on health services utilization in Province-2 of Nepal: a qualitative study among community members and stakeholders," *BMC Health Serv Res*, vol. 21, no. 1, p. 174, Feb. 2021, doi: 10.1186/s12913-021-06176-y.

[14] B. Rayamajhee *et al.*, "How Well the Government of Nepal Is Responding to COVID-19? An Experience From a Resource-Limited Country to Confront Unprecedented Pandemic," *Front. Public Health*, Feb. 2021, [Online]. Available: https://doi.org/10.3389/fpubh.2021.597808

[15] T. R. Pradhan, "Nepal goes under lockdown for a week starting 6am Tuesday," *The Kathmandu Post*, Mar. 23, 2020. [Online]. Available:

For peer review only - http://bmjopen.bmj.com/site/about/guidelines.xhtml

https://kathmandupost.com/national/2020/03/23/nepal-goes-under-lockdown-for-a-week-starting-6am-tuesday

[16] T. R. Pradhan, “Government decides to lift the four-month-long coronavirus lockdown, but with conditions,” *The Kathmandu Post*, Jul. 21, 2020. [Online]. Available: https://kathmandupost.com/national/2020/07/21/government-decides-to-lift-the-four-month-long-coronavirus-lockdown-but-with-conditions

[17] “Annual report 2076/77 (2019/20),” Department of Health Services, Ministry of Health and Population, Government of Nepal, Jul. 2021. [Online]. Available: https://dohs.gov.np/annual-report-2076-77-2019-20/

[18] “The Situation of Corona Virus (COVID-19) in Nepal: Daily Reports,” *INSECOnline*. [Online]. Available: http://inseconline.org/en/covid-19/

[19] “Government of Nepal, Office of the Prime Minister and Council of Ministers, Covid-19 Crisis Management Coordination Center (CCMCC),” *CCMCC*. https://ccmc.gov.np/

[20] “Government of Nepal, Ministry of Home Affairs,” *Offices*. https://www.moha.gov.np/pro_admin/api/offices (accessed Aug. 03, 2022).

[21] “CoVid-19 Dasboard,” Government of Nepal, Ministry of Health and Population. Accessed: Feb. 09, 2021. [Online]. Available: https://covid19.mohp.gov.np/

[22] “Module 2: Desk review of data quality,” World Health Organization, 2, 2017.

[23] J. Angrist and J.-S. Pischke, “Parallel worlds: fixed effects, difference-in-differences, and panel data,” in *Mostly Harmless Econometrics: An Empiricist’s Companion*, Princeton, NJ: Princeton University Press, 2008, pp. 221–47.

[24] “Preliminary Data of National Population and Housing Census 2021,” Government of Nepal, National Planning Commission Central Bureau of Statistics, Jan. 2022. [Online]. Available: https://opendatanepal.com/dataset/preliminary-data-of-national-population-and-housing-census-2021

[25] “Nepal Police crack down on 749 Kathmandu residents for violating Covid-19 lockdown,” *India Today*, Mar. 30, 2020. [Online]. Available: https://www.indiatoday.in/world/story/nepal-police-crackdown-on-749-kathmandu-residents-for-violating-covid-19-lockdown-1661343-2020-03-30

[26] U. N. Yadav *et al.*, “Perceived fear of COVID-19 and its associated factors among Nepalese older adults in eastern Nepal: A cross-sectional study,” *PLOS ONE*, vol. 16, no. 7, p. e0254825, Jul. 2021, doi: 10.1371/journal.pone.0254825.

[27] R. Cash and V. Patel, “Has COVID-19 subverted global health?,” *Lancet*, vol. 395, no. 10238, pp. 1687–1688, 2020, doi: 10.1016/S0140-6736(20)31089-8.

[28] “Free Health Care in Nepal: Findings of a Rapid Assessment,” GFA Consulting Group. [Online]. Available: https://files.givewell.org/files/DWDA%202009/Nyaya/en-free-health-care-nepal.pdf

[29] S. P. Kalaunee, “Is free health care possible in Nepal?,” *Nepali Times*, Dec. 12, 2019. Accessed: Dec. 03, 2021. [Online]. Available: https://www.nepalitimes.com/opinion/is-free-health-care-possible-in-nepal/

[30] C. Arsenault *et al.*, “Hospital-provision of essential primary care in 56 countries: determinants and quality,” *Bull World Health Organ*, vol. 98, no. 11, pp. 735-746D, Nov. 2020, doi: 10.2471/BLT.19.245563.

[31] S. Acharya, S. Ghimire, E. M. Jeffers, and N. Shrestha, “Health Care Utilization and Health Care Expenditure of Nepali Older Adults,” *Front Public Health*, vol. 7, p. 24, Feb. 2019, doi: 10.3389/fpubh.2019.00024.

For peer review only - http://bmjopen.bmj.com/site/about/guidelines.xhtml

[32] "Responding to COVID-19: Health sector preparedness, response and lessons learnt," Government of Nepal, Ministry of Health and Population, Kathmandu, Nepal, 2021.
[33] D. Banta, "What is the efficacy/effectiveness of antenatal care and the financial and organizational implications?," WHO Regional Office for Europe, Copenhagen, Dec. 2003. [Online]. Available: https://www.euro.who.int/__data/assets/pdf_file/0007/74662/E82996.pdf
[34] Riley, Sully, Ahmed, and Biddlecom, "Estimates of the Potential Impact of the COVID-19 Pandemic on Sexual and Reproductive Health In Low- and Middle-Income Countries," *International Perspectives on Sexual and Reproductive Health*, vol. 46, p. 73, 2020, doi: 10.1363/46e9020.
[35] G. Weigel, A. Salganicoff, and U. Ranji, "Potential Impacts of Delaying 'Non-Essential' Reproductive Health Care," Kaiser Family Foundation, Jun. 2020.
[36] Bernadeta Dadonaite, *Pneumonia — no child should die from a disease we can prevent*, 2019. https://ourworldindata.org/child-deaths-from-pneumonia
[37] A. Rouw, A. Wexler, J. Kates, and J. Michaud, "Global COVID-19 Vaccine Access: A Snapshot of Inequality," Kaiser Family Foundation, Mar. 2021. Accessed: Dec. 03, 2021. [Online]. Available: https://www.kff.org/policy-watch/global-covid-19-vaccine-access-snapshot-of-inequality/
[38] A. Grimsrud and L. Wilkinson, "Acceleration of differentiated service delivery for HIV treatment in sub-Saharan Africa during COVID-19," *J Int AIDS Soc*, vol. 24, no. 6, p. e25704, Jun. 2021, doi: 10.1002/jia2.25704.
[39] E. Dong, H. Du, and L. Gardner, "An interactive web-based dashboard to track COVID-19 in real time," *The Lancet Infectious Diseases*, vol. 20, no. 5, pp. P533-534, May 2020, doi: 10.1016/S1473-3099(20)30120-1.

For peer review only - http://bmjopen.bmj.com/site/about/guidelines.xhtml

Figure 1. Total COVID cases and policy responses in Nepal from January 1, 2020, to September 16, 2020

The first recorded COVID case was reported on January 25th, 2020. The federal government implemented a nation-wide lockdown on March 24, 2020, including: stay-at-home requirements, closure of non-essential businesses, schools and all public transport, and restrictions on gathering and internal movements.[14] The National lockdown was lifted four months later on July 22, 2020, with major restrictions lifted, including stay-at-home requirements, workplace and public transport closures after which palika-specific response was allowed.
Source: COVID Data Repository by the Center for Systems Science and Engineering (CSSE) at Johns Hopkins University [39]

Figure 2. Primary care service utilization from Magh 2075 to Ashwin 2077 in Nepal (January 15, 2019 to September 16, 2020)

Months 1 to 20 are January 15, 2019 to September 16, 2020 (Magh 2075 to Bhadra 2077). For the purpose of our analysis, the national lockdown period (pre-period) includes months 15 to 18 (March 13 to July 16, 2020, Chaitra 2076 to Ashar 2077) and the post-period is Month 20 (August 17 to September 16, 2020, equivalent to Bhadra 2077). The orange lines represent average health care utilization in the control group: palikas that maintained COVID restrictions in the post period (e.g., stay-at-home requirements, business and public transport closures). The green lines represent health care utilization in the treatment group: palikas that lifted COVID restrictions in the post-period. Detailed definitions of health service indicators are in supplemental materials.

For peer review only

For peer review only - http://bmjopen.bmj.com/site/about/guidelines.xhtml

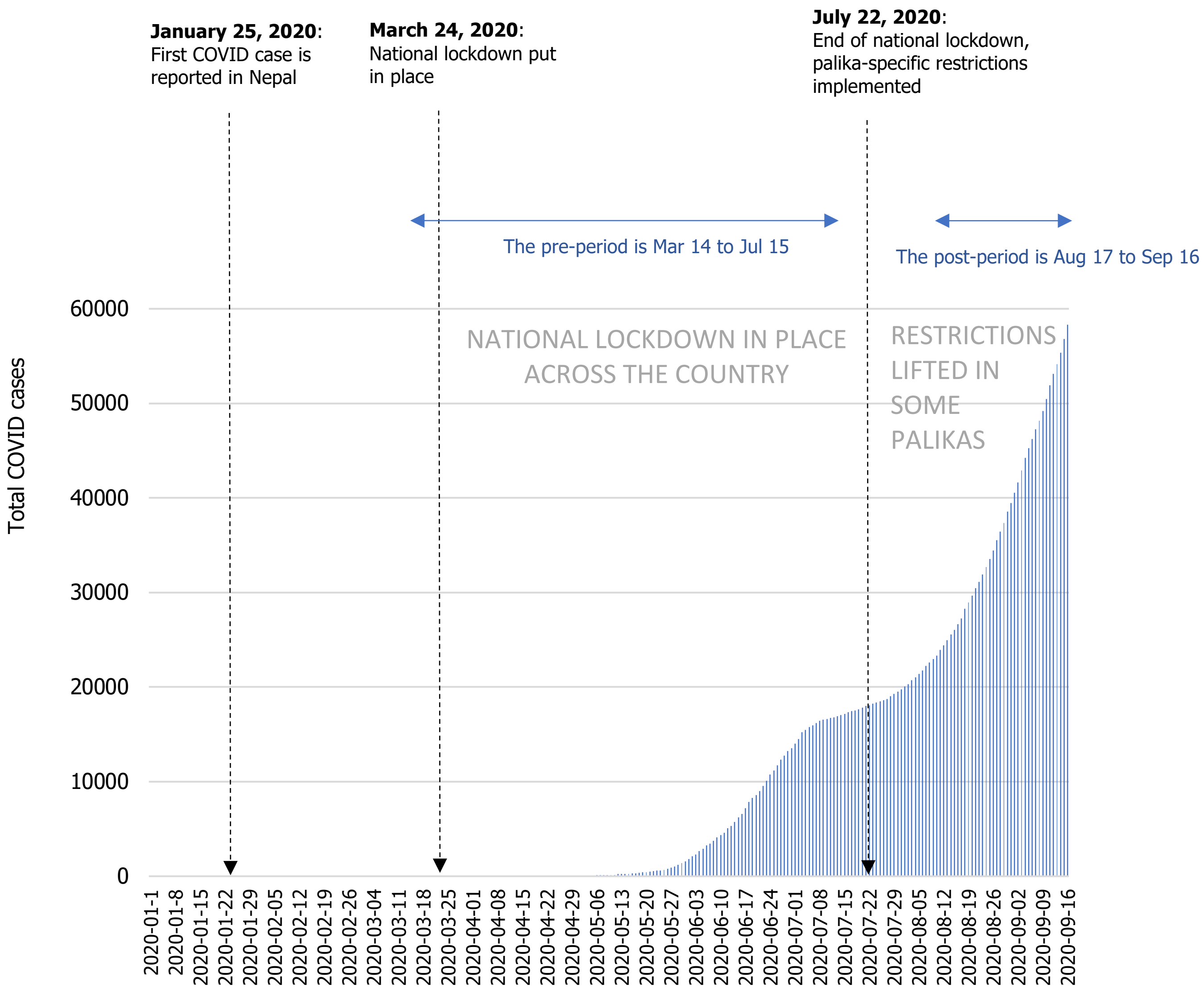
January 25, 2020: First COVID case is reported in Nepal
March 24, 2020: National lockdown put in place
July 22, 2020: End of national lockdown, palika-specific restrictions implemented
The pre-period is Mar 14 to Jul 15
The post-period is Aug 17 to Sep 16
NATIONAL LOCKDOWN IN PLACE ACROSS THE COUNTRY
RESTRICTIONS LIFTED IN SOME PALIKAS
Total COVID cases
60000
50000
40000
30000
20000
10000
0
2020-01-1
2020-01-8
2020-01-15
2020-01-22
2020-01-29
2020-02-05
2020-02-12
2020-02-19
2020-02-26
2020-03-04
2020-03-11
2020-03-18
2020-03-25
2020-04-01
2020-04-08
2020-04-15
2020-04-22
2020-04-29
2020-05-06
2020-05-13
2020-05-20
2020-05-27
2020-06-03
2020-06-10
2020-06-17
2020-06-24
2020-07-01
2020-07-08
2020-07-15
2020-07-22
2020-07-29
2020-08-05
2020-08-12
2020-08-19
2020-08-26
2020-09-02
2020-09-09
2020-09-16

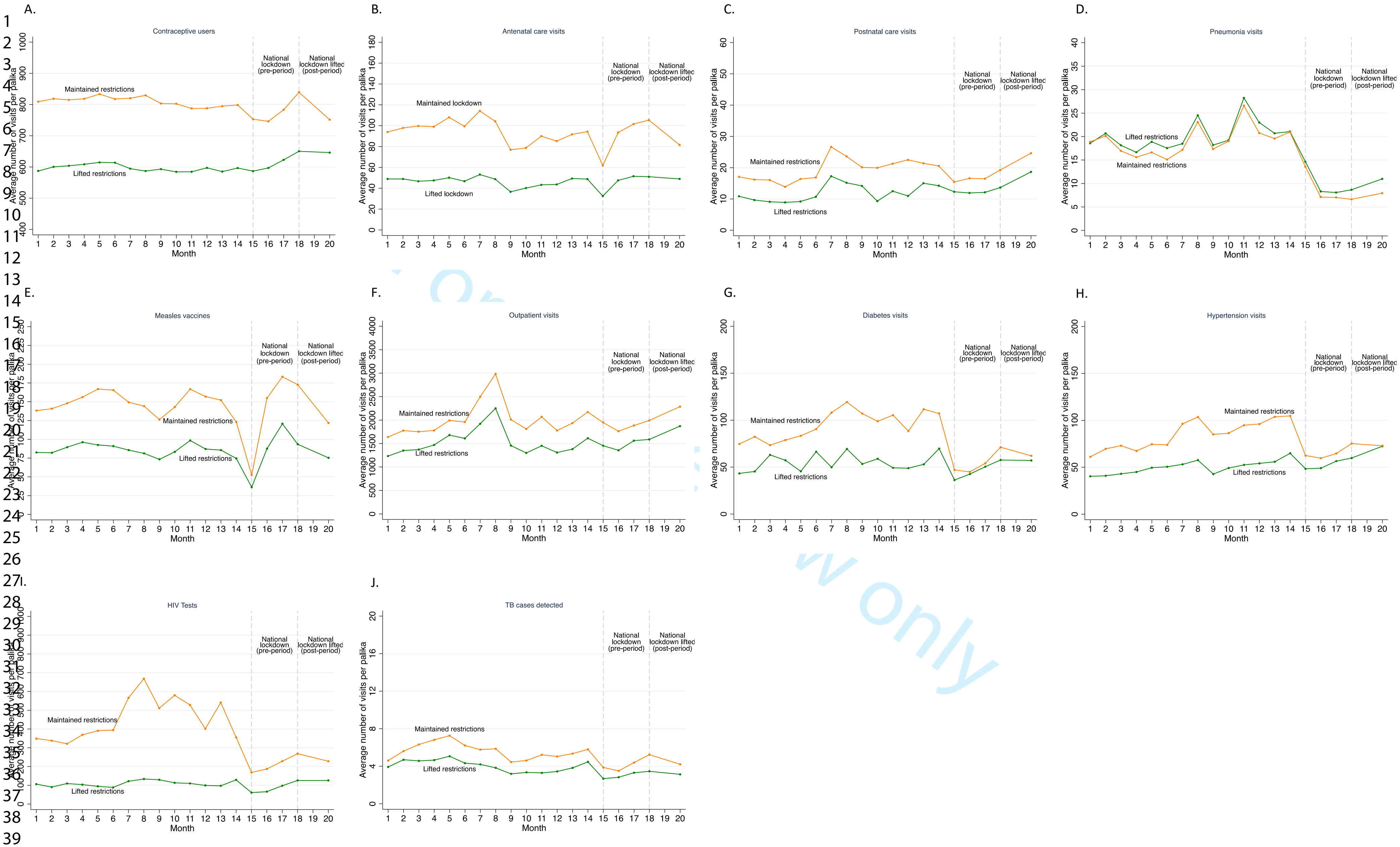
A.
Contraceptive users
Maintained restrictions
Lifted restrictions
B.
Antenatal care visits
Maintained lockdown
Lifted lockdown
C.
Postnatal care visits
Maintained restrictions
Lifted restrictions
D.
Pneumonia visits
Lifted restrictions
Maintained restrictions
E.
Measles vaccines
Maintained restrictions
Lifted restrictions
F.
Outpatient visits
Maintained restrictions
Lifted restrictions
G.
Diabetes visits
Maintained restrictions
Lifted restrictions
H.
Hypertension visits
Maintained restrictions
Lifted restrictions
I.
HIV Tests
Maintained restrictions
Lifted restrictions
J.
TB cases detected
Maintained restrictions
Lifted restrictions
National lockdown (pre-period)
National lockdown lifted (post-period)
Average number of visits per palika
Month

Title: The effect of lifting COVID-19 restrictions on utilization of primary care services in Nepal: a difference-in-differences analysis

Authors: Kapoor NR, Aryal A, Mehata S, Dulal M, Kruk ME, Bauhoff S, Arsenault C

Supplemental materials

Supplemental Table 1. Additional sources used for COVID-19 restrictions tracking

**Additional Sources:**
48 dists currently under DAO prohibitory order. The Rising Nepal. 2020; published online Aug 31. https://old.risingnepaldaily.com/main-news/48-dists-currently-under-dao-prohibitory-order.
Curfew Relaxed In Kaski, Public Buses Not Plying, Taxis And Private Vehicles Rampant. Arthik Awaj. 2020; published online Aug 28. https://arthikawaj.com/%e0%a4%aa%e0%a5%8b%e0%a4%96%e0%a4%b0%e0%a4%be%e0%a4%ae%e0%a4%be-%e0%a4%a8%e0%a4%bf%e0%a4%b6%e0%a5%87%e0%a4%a7%e0%a4%be%e0%a4%9c%e0%a5%8d%e0%a4%9e%e0%a4%be-%e0%a4%96%e0%a5%81%e0%a4%95%e0%a5%81%e0%a4%b2/.
Four local levels in Jhapa impose weeklong lockdown. Nepal Monitor. 2020; published online Aug 15. https://nepalmonitor.org/reports/view/30662.
Indefinite prohibitory order imposed in Bara. Khabarhub. 2020; published online Aug 17. https://english.khabarhub.com/2020/17/120466/.
INFOGRAPHICS: 48 districts have partial or complete lockdown. myRepublica. 2020; published online Aug 24. http://myrepublica.nagariknetwork.com/news/98460/.
INFOGRAPHICS: These 52 districts are under lockdown. myRepublica. 2020; published online Aug 25. https://myrepublica.nagariknetwork.com/news/infographics-these-52-districts-are-under-lockdown/.
Kathmandu Valley back to lockdown. Nepali Times. 2020; published online Aug 18. https://www.nepalitimes.com/banner/kathmandu-valley-back-to-lockdown/.
Local levels in Kavrepalanchok being sealed off amidst COVID-19 fear. Khabarhub. 2020; published online Aug 15. https://english.khabarhub.com/2020/15/120082/.
Lockdown enforced in Byas Municipality for a week. Nepal Monitor. 2020; published online Aug 17. https://nepalmonitor.org/reports/view/30696.
Lockdown enforced in Dasarathchand Municipality of Baitadi. Nepal Monitor. 2020; published online Aug 20. https://nepalmonitor.org/reports/view/30790.
Lockdown enforced in five local levels of Sindhuli. Nepal Monitor. 2020; published online Aug 15. https://nepalmonitor.org/reports/view/30667.
Koirala N. Lockdown extended for another week in Bhojpur. The Himalayan Times. 2020; published online Sept 10. https://thehimalayantimes.com/nepal/lockdown-extended-for-another-week-in-bhojpur.

For peer review only - http://bmjopen.bmj.com/site/about/guidelines.xhtml

Nepal: Lockdown extended in Kathmandu Valley until September 16. Crisis 24. 2020; published online Sept 10. https://crisis24.garda.com/alerts/2020/09/nepal-lockdown-extended-in-kathmandu-valley-until-september-16-update-24.
Rai MC. Prohibition in 3 cities including Udaipur headquarters. Nagarik News. 2020; published online Aug 12. https://nagariknews.nagariknetwork.com/social-affairs/304061-1597214771.html.
Prohibition in Dang from 14th. Online Khabar. 2020; published online Aug 12. https://www.onlinekhabar.com/2020/08/892463.
Prohibitory order in Dhankuta extended by one week. Khabarhub. 2020; published online Aug 27. https://english.khabarhub.com/2020/27/123095/.
Prohibitory orders in 39 districts to curb COVID-19. The Rising Nepal. 2020; published online Aug 17. https://old.risingnepaldaily.com/mustread/prohibitory-orders-in-39-districts-to-curb-covid-19.
Ten days long lockdown imposed in Sankhuwasabha. Nepal Monitor. 2020; published online Aug 18. https://nepalmonitor.org/reports/view/30747.
Gautam L. Week-long lockdown imposed to prevent spread of coronavirus in Phidim. The Himalayan Times. 2020; published online Aug 28. https://thehimalayantimes.com/nepal/week-long-lockdown-imposed-to-prevent-spread-of-coronavirus-in-phidim.

For peer review only

Supplemental Table 2. Nepal DHIS2 Definitions for health service outcomes

Health service	Nepal DHIS2 Definition
**Outpatient visits**	Disaggregation by Sex & Caste/Ethnicity - Outpatient Cases
**Family planning**	Family Planning Program - Temporary FP Method - Depo-Current User +<br>Family Planning Program - Temporary FP Method - Depo-New Users < 20 Years +<br>Family Planning Program - Temporary FP Method - Depo-New Users > 20 Years +<br>Family Planning Program - Temporary FP Method - Pills- Current User +<br>Family Planning Program - Temporary FP Method - Pills- < 20 Years +<br>Family Planning Program - Temporary FP Method - Pills- > 20 Years +<br>Safe Motherhood Program-Safe Abortion Service-Post Abortion FP Methods Short Term-Medical +<br>Safe Motherhood Program-Safe Abortion Service-Post Abortion FP Methods Short term-Surgical +
**Antenatal care**	Safe Motherhood Program-Antenatal Checkup-First ANC visits (any time) < 20 years +<br>Safe Motherhood Program-Antenatal Checkup-First ANC visits (any time) > 20 years
**Postnatal care**	Safe Motherhood Program- Type of Delivery - 3 PNC visits as per protocol
**Pneumonia**	CBIMCI-(2-59Months)- Classification-ARI-Pneumonia +<br>CBIMCI-(2-59Months)-ORC Classification-ARI-Severe Pneumonia/Very Severe Disease
**Measles**	Immunization program - Children Immunized - Measles/Rubella - 9-11 Months + Immunization program - Children Immunized - Measles/Rubella - 12-23 Months
**HIV tests**	Virology-HIV tests conducted
**TB detection**	Disaggregation by Sex & Caste/Ethnicity- New TB Cases
**Diabetes visits**	Outpatient Morbidity-Nutritional & Metabolic Disorder-Diabetes Mellitus (DM) Cases
**Hypertension visits**	OPD-Morbidity-Cardiovascular & Respiratory Related Problems-Hypertension

Nepal DHIS2 definitions for primary care services.
FP – family planning
ANC – antenatal care
CBIMCI - Community Based Integrated Management of Childhood Illness
HIV – Human immunodeficiency virus
TB – Tuberculosis
OPD – outpatient department

For peer review only

Supplemental Table 3. Level of COVID-19 Restrictions in place from March 14, to September 16, 2020 in Nepal

	Mar 14, 2020 - April 12, 2020 (Chatira 2076)	April 13, 2020 - May 13, 2020 (Baisakh 2077)	May 14, 2020 - June 14, 2020 (Jestha 2077)	June 15, 2020 - July 15, 2020 (Ashar 2077)	July 16, 2020 - August 16, 2020 (Shrawan 2077)	August 17, 2020 - September 16, 2020 (Bhadra 2077)
Stay-at-home required (except essentials)	National[1]	National	National	National	District or palika-specific[3]	District or palika-specific
Business/workplace closures required	National[1]	National	National	National	District or palika-specific[3]	District or palika-specific
Public transport closures	National[1]	National	National	National	District or palika-specific[3]	District or palika-specific
Restricted gatherings to <10	National[1]	National	National	National	National	National
Border closure	National[1]	National	National	National	National	National
School closures	National[1]	National	National	National	National	National
Restrictions on internal movement	National[1]	National	National	National	National	National

[1]These policies were put in place on or around March 22nd, 2020.
[2]These policies were lifted on or around July 22nd, 2020.

Sources:
Rayamajhee B, Pojhrel A, Syangtan G, *et al.* How Well the Government of Nepal Is Responding to COVID-19? An Experience From a Resource-Limited Country to Confront Unprecedented Pandemic. *Front Public Health* 2021; published online Feb 17. https://doi.org/10.3389/fpubh.2021.597808.

Thomas Hale, Noam Angrist, Rafael Goldszmidt, *et al.* A global panel database of pandemic policies policies (Oxford COVID-19 Government Response Tracker). *Nature Human Behaviour* 2021. DOI:10.1038/s41562-021-01079-8.

The Situation of Corona Virus (COVID-19) in Nepal: Daily Reports. INSECOnline. http://inseconline.org/en/covid-19/.

Pradhan TR. Nepal goes under lockdown for a week starting 6am Tuesday. The Kathmandu Post. 2020; published online March 23. https://kathmandupost.com/national/2020/03/23/nepal-goes-under-lockdown-for-a-week-starting-6am-tuesday.

Pradhan TR. Government decides to lift the four-month-long coronavirus lockdown, but with conditions. The Kathmandu Post. 2020; published online July 21. https://kathmandupost.com/national/2020/07/21/government-decides-to-lift-the-four-month-long-coronavirus-lockdown-but-with-conditions.

For peer review only

Supplemental Table 4. Joint F-tests for parallel trends assessment

	May * Restrictions lifted & June * Restrictions lifted Joint F-test (p-value)
Contraceptive Users	0.43
ANC Visits	0.16
PNC Visits	0.46
Child pneumonia visits	0.29
Measles vaccine	0.24
Outpatient visits	0.32
Diabetes visits	0.52
Hypertension visits	0.11
HIV tests	0.86
TB cases detected	0.16

P-values for joint F-test for *May * Restrictions lifted* and *June* Restrictions lifted* to test they are jointly not significantly different from zero.
May is May 14, 2020 to June 14, 2020 (Jestha 2077) and *June* is June 15, 2020 to July 15, 2020 (Ashar 2077). May and June are in the pre-period, during the national lockdown. The coefficients for *May*Restrictions lifted* and *June*Restrictions lifted* assess if trends are parallel in the pre-period, if the effect of lifting COVID-19 restrictions in palikas is significantly different from the baseline month, April 13 2020 to May 13 2020 (Baisakh 2076).

For peer review only

Supplemental Table 5. Estimated effect of lifting COVID-19 restrictions on primary care service utilization in Nepal, estimates from difference-in-differences models that exclude March 14, 2020 – April 12, 2020 (Chaitra 2076)

	Restrictions lifted	95% CI	COVID-19 cases	95% CI	N	R^2	adj. R^2
**Contraceptive users**	56.40*	[11.63,101.17]	-0.01	[-0.03,0.01]	2968	0.01	0.01
**ANC Visits**	18.32***	[8.31,28.33]	0.00	[-0.01,0.01]	2900	0.03	0.03
**PNC Visits**	-1.06	[-4.89,2.78]	0.00	[-0.00,0.00]	2076	0.07	0.07
**Child pneumonia visits**	1.45*	[0.11,2.80]	-0.00**	[-0.00,-0.00]	2220	0.03	0.03
**Measles vaccine**	17.60*	[2.69,32.50]	-0.01**	[-0.01,-0.00]	1392	0.08	0.07
**Outpatient visits**	-84.61	[-223.74,54.53]	-0.10***	[-0.13,-0.06]	2968	0.10	0.10
**Diabetes visits**	3.61	[-8.74,15.95]	0.00	[-0.01,0.02]	1332	0.02	0.02
**Hypertension visits**	11.95	[-7.68,31.58]	0.00	[-0.01,0.01]	2756	0.02	0.01
**HIV tests**	33.24	[-11.71,78.20]	0.01	[-0.01,0.03]	944	0.04	0.03
**TB cases detected**	-0.02	[-0.83,0.78]	0.00	[-0.00,0.00]	920	0.05	0.05

95% confidence intervals in brackets
* $p < 0.05$, ** $p < 0.01$, *** $p < 0.001$
The coefficient for *Restrictions lifted* is the effect of lifting COVID-19 restrictions on health service utilization. Models also included fixed effects for month and palikas.

For peer review only

Supplemental Table 6. Number of palika and volume of services in the raw vs. final dataset

		Number of palika reporting			Sum of services		
**Health service**	**Variable name**	**Raw data**	**Final data**	**% difference**	**Raw data**	**Final data**	**% difference**
Contraceptive users	fp_sa_util	753	742	-1%	2,702,347	2,685,227	-1%
Antenatal care	anc_util	752	725	-4%	276,465	271,893	-2%
Postnatal care visits	pnc_util	717	519	-28%	46,846	43,617	-7%
Child pneumonia visits	pneum_util	745	555	-26%	27,164	25,030	-8%
Outpatient visits	opd_util	753	742	-1%	6,866,933	6,828,125	-1%
TB cases detected	tbdetect_qual	679	230	-66%	6,572	4,577	-30%
Measles Vaccine	measles_qual	753	348	-54%	361,213	210,662	-42%
Hypertension visits	hyper_util	752	689	-8%	222,659	220,033	-1%
Diabetes visits	diab_util	627	333	-47%	93,261	89,390	-4%
HIV tests	hivtest_qual	520	236	-55%	227,166	213,789	-6%

For peer review only

For peer review only - http://bmjopen.bmj.com/site/about/guidelines.xhtml

STROBE Statement—Checklist of items that should be included in reports of ***cross-sectional studies***

	Item No	Recommendation	Page No
**Title and abstract**	1	(*a*) Indicate the study's design with a commonly used term in the title or the abstract	1, 2
		(*b*) Provide in the abstract an informative and balanced summary of what was done and what was found	2
**Introduction**			
Background/rationale	2	Explain the scientific background and rationale for the investigation being reported	4
Objectives	3	State specific objectives, including any prespecified hypotheses	4,5
**Methods**			
Study design	4	Present key elements of study design early in the paper	6
Setting	5	Describe the setting, locations, and relevant dates, including periods of recruitment, exposure, follow-up, and data collection	6
Participants	6	(*a*) Give the eligibility criteria, and the sources and methods of selection of participants	6,7
Variables	7	Clearly define all outcomes, exposures, predictors, potential confounders, and effect modifiers. Give diagnostic criteria, if applicable	7
Data sources/ measurement	8*	For each variable of interest, give sources of data and details of methods of assessment (measurement). Describe comparability of assessment methods if there is more than one group	6,7
Bias	9	Describe any efforts to address potential sources of bias	7
Study size	10	Explain how the study size was arrived at	6,7
Quantitative variables	11	Explain how quantitative variables were handled in the analyses. If applicable, describe which groupings were chosen and why	6,7
Statistical methods	12	(*a*) Describe all statistical methods, including those used to control for confounding	7
		(*b*) Describe any methods used to examine subgroups and interactions	7
		(*c*) Explain how missing data were addressed	7
		(*d*) If applicable, describe analytical methods taking account of sampling strategy	7
		(*e*) Describe any sensitivity analyses	7
**Results**			
Participants	13*	(a) Report numbers of individuals at each stage of study—eg numbers potentially eligible, examined for eligibility, confirmed eligible, included in the study, completing follow-up, and analysed	9, 17
		(b) Give reasons for non-participation at each stage	9
		(c) Consider use of a flow diagram	NA
Descriptive data	14*	(a) Give characteristics of study participants (eg demographic, clinical, social) and information on exposures and potential confounders	9, 17, 18
		(b) Indicate number of participants with missing data for each variable of interest	9,17
Outcome data	15*	Report numbers of outcome events or summary measures	9,17
Main results	16	(*a*) Give unadjusted estimates and, if applicable, confounder-adjusted estimates and their precision (eg, 95% confidence interval). Make clear which confounders were adjusted for and why they were included	9, 17, 20

For peer review only - http://bmjopen.bmj.com/site/about/guidelines.xhtml

		(*b*) Report category boundaries when continuous variables were categorized	NA
		(*c*) If relevant, consider translating estimates of relative risk into absolute risk for a meaningful time period	9
Other analyses	17	Report other analyses done—eg analyses of subgroups and interactions, and sensitivity analyses	9
**Discussion**			
Key results	18	Summarise key results with reference to study objectives	10
Limitations	19	Discuss limitations of the study, taking into account sources of potential bias or imprecision. Discuss both direction and magnitude of any potential bias	11
Interpretation	20	Give a cautious overall interpretation of results considering objectives, limitations, multiplicity of analyses, results from similar studies, and other relevant evidence	10, 11
Generalisability	21	Discuss the generalisability (external validity) of the study results	11, 12
**Other information**			
Funding	22	Give the source of funding and the role of the funders for the present study and, if applicable, for the original study on which the present article is based	12

*Give information separately for exposed and unexposed groups.

Note: An Explanation and Elaboration article discusses each checklist item and gives methodological background and published examples of transparent reporting. The STROBE checklist is best used in conjunction with this article (freely available on the Web sites of PLoS Medicine at http://www.plosmedicine.org/, Annals of Internal Medicine at http://www.annals.org/, and Epidemiology at http://www.epidem.com/). Information on the STROBE Initiative is available at www.strobe-statement.org.

For peer review only
